# Supplementary material for: A guanosine tetraphosphate (ppGpp) mediated brake on photosynthesis is required for acclimation to nitrogen limitation in Arabidopsis
Source: eLife. 2022 Feb 14;11:e75041. doi: 10.7554/eLife.75041 (PMC8887892; doi:10.7554/eLife.75041)
Supplement: Figure 1—source data 1. [file elife-75041-fig1-data1.zip › Fig 1 source data/Fig_1C_data_statistics.html]

Proportion test and CI calculation


# Proportion test and CI calculation

####

## 1. Introduction

The analysis reported here is part of the manuscript Romand et al. 2021. The script performs statistical analysis on categorical/nominal data: comparison of luminescent seedling counts between different plant lines grown under N limiting conditions and imaged for peroxidated lipids.

The analysis requires the rstatix package and the rcompanion package.

#### Setup R packages

```
if (!require(rstatix)) { install.packages("rstatix", repos = "http://cran.us.r-project.org") }
if (!require(rcompanion)) { install.packages("rcompanion", repos = "http://cran.us.r-project.org") }
if (!require(knitr)) { install.packages("knitr", repos = "http://cran.us.r-project.org") }
if (!require(tidyverse)) { install.packages("tidyverse", repos = "http://cran.us.r-project.org") }
if (!require(devtools)) { install.packages("devtools", repos = "http://cran.us.r-project.org") }


library(rstatix)
library(rcompanion)
library(knitr)
library(tidyverse)
library(devtools)
```

## 2. Data

Data is inputted manually into a table. Luminiscent is for plants showing luminesence, and all is the total number of plants evaluated.

```
# Create data matrix for analysis

xtab <- matrix(c(5, 100, 25, 100, 8, 100, 0, 100, 27, 100 ), nrow = 5, ncol = 2, byrow = TRUE,
               dimnames = list(c("Col", "OXRSH1", "qrt", "rsh1-1","QM"),
                               c("luminescent", "all")))

kable(xtab)
```

|  | luminescent | all |
| --- | --- | --- |
| Col | 5 | 100 |
| OXRSH1 | 25 | 100 |
| qrt | 8 | 100 |
| rsh1-1 | 0 | 100 |
| QM | 27 | 100 |

## 3. Data analysis

A proportion test is performed using the proportion test (prop\_test) from the rstatix package.

```
table<-prop_test(xtab, conf.level = 0.95, detailed=TRUE, correct=TRUE)
kable(table)
```

| n | n1 | n2 | n3 | n4 | n5 | estimate1 | estimate2 | estimate3 | estimate4 | estimate5 | statistic | p | df | method | alternative | p.signif |
| --- | --- | --- | --- | --- | --- | --- | --- | --- | --- | --- | --- | --- | --- | --- | --- | --- |
| 565 | 105 | 125 | 108 | 100 | 127 | 0.047619 | 0.2 | 0.0740741 | 0 | 0.2125984 | 40.2024 | 0 | 4 | Prop test | two.sided | \*\*\*\* |

Now a post-hoc pairwaise Fisher test is performed to identify which differences are signficant using the rcompanion package. See `https://rcompanion.org/handbook/H_04.html` for more information.

```
PT = pairwiseNominalIndependence(xtab,
                                 compare = "row",
                                 fisher  = TRUE,
                                 gtest   = FALSE,
                                 chisq   = FALSE,
                                 method  = "fdr",  # False Discovery Rate, see ?p.adjust for options
                                 digits  = 3)

# Add a significance column

PT<-PT %>%
  mutate(Significance = case_when(
    p.adj.Fisher <=0.01 ~ "**",
    p.adj.Fisher <=0.05 ~ "*",
    p.adj.Fisher >=0.05 ~ "ns",
    ))

kable(PT)
```

| Comparison | p.Fisher | p.adj.Fisher | Significance |
| --- | --- | --- | --- |
| Col : OXRSH1 | 6.48e-04 | 1.62e-03 | \*\* |
| Col : qrt | 5.69e-01 | 6.32e-01 | ns |
| Col : rsh1-1 | 5.98e-02 | 7.48e-02 | ns |
| Col : QM | 2.24e-04 | 7.47e-04 | \*\* |
| OXRSH1 : qrt | 7.73e-03 | 1.10e-02 | \* |
| OXRSH1 : rsh1-1 | 1.00e-07 | 7.00e-07 | \*\* |
| OXRSH1 : QM | 8.77e-01 | 8.77e-01 | ns |
| qrt : rsh1-1 | 7.10e-03 | 1.10e-02 | \* |
| qrt : QM | 3.11e-03 | 6.22e-03 | \*\* |
| rsh1-1 : QM | 1.00e-07 | 7.00e-07 | \*\* |

Next confidence intervals are calculated using the binomial test.

```
data<-as.data.frame(xtab)

# Add columns to show line names and proportion luminescent 

data$Lines <- row.names(data)

data$luminescent_proportion = data$luminescent / data$all

data<-data[,c(3,1,2,4)]

# Perform binom test and extract confidence intervals

data<-data %>% 
    rowwise() %>% 
    mutate(CI= list(enframe(binom.test(luminescent, all, alternative =  "two.sided", conf.level = 0.95)$conf.int))) %>% 
    unnest(CI) %>% 
    spread(name, value) %>% 
    rename("LCB" = "1", "UCB" = "2")


kable(data, digits= 3)
```

| Lines | luminescent | all | luminescent\_proportion | LCB | UCB |
| --- | --- | --- | --- | --- | --- |
| Col | 5 | 100 | 0.05 | 0.016 | 0.113 |
| OXRSH1 | 25 | 100 | 0.25 | 0.169 | 0.347 |
| QM | 27 | 100 | 0.27 | 0.186 | 0.368 |
| qrt | 8 | 100 | 0.08 | 0.035 | 0.152 |
| rsh1-1 | 0 | 100 | 0.00 | 0.000 | 0.036 |

## 4. R session

```
InfoSession <- devtools::session_info()
print(InfoSession)
```

```
## - Session info ---------------------------------------------------------------
##  setting  value                       
##  version  R version 4.1.0 (2021-05-18)
##  os       Windows 10 x64              
##  system   x86_64, mingw32             
##  ui       RTerm                       
##  language (EN)                        
##  collate  English_United States.1252  
##  ctype    English_United States.1252  
##  tz       Europe/Paris                
##  date     2021-11-02                  
## 
## - Packages -------------------------------------------------------------------
##  package      * version date       lib source        
##  abind          1.4-5   2016-07-21 [1] CRAN (R 4.1.0)
##  assertthat     0.2.1   2019-03-21 [1] CRAN (R 4.1.0)
##  backports      1.2.1   2020-12-09 [1] CRAN (R 4.1.0)
##  boot           1.3-28  2021-05-03 [2] CRAN (R 4.1.0)
##  broom          0.7.8   2021-06-24 [1] CRAN (R 4.1.0)
##  cachem         1.0.5   2021-05-15 [1] CRAN (R 4.1.0)
##  callr          3.7.0   2021-04-20 [1] CRAN (R 4.1.0)
##  car            3.0-11  2021-06-27 [1] CRAN (R 4.1.0)
##  carData        3.0-4   2020-05-22 [1] CRAN (R 4.1.0)
##  cellranger     1.1.0   2016-07-27 [1] CRAN (R 4.1.0)
##  class          7.3-19  2021-05-03 [2] CRAN (R 4.1.0)
##  cli            2.5.0   2021-04-26 [1] CRAN (R 4.1.0)
##  codetools      0.2-18  2020-11-04 [2] CRAN (R 4.1.0)
##  coin           1.4-1   2021-02-08 [1] CRAN (R 4.1.0)
##  colorspace     2.0-2   2021-06-24 [1] CRAN (R 4.1.0)
##  crayon         1.4.1   2021-02-08 [1] CRAN (R 4.1.0)
##  curl           4.3.1   2021-04-30 [1] CRAN (R 4.1.0)
##  data.table     1.14.0  2021-02-21 [1] CRAN (R 4.1.0)
##  DBI            1.1.1   2021-01-15 [1] CRAN (R 4.1.0)
##  dbplyr         2.1.1   2021-04-06 [1] CRAN (R 4.1.0)
##  desc           1.3.0   2021-03-05 [1] CRAN (R 4.1.0)
##  DescTools      0.99.42 2021-06-17 [1] CRAN (R 4.1.0)
##  devtools     * 2.4.2   2021-06-07 [1] CRAN (R 4.1.0)
##  digest         0.6.27  2020-10-24 [1] CRAN (R 4.1.0)
##  dplyr        * 1.0.7   2021-06-18 [1] CRAN (R 4.1.0)
##  e1071          1.7-7   2021-05-23 [1] CRAN (R 4.1.0)
##  ellipsis       0.3.2   2021-04-29 [1] CRAN (R 4.1.0)
##  evaluate       0.14    2019-05-28 [1] CRAN (R 4.1.0)
##  Exact          2.1     2020-10-02 [1] CRAN (R 4.1.0)
##  expm           0.999-6 2021-01-13 [1] CRAN (R 4.1.0)
##  fansi          0.5.0   2021-05-25 [1] CRAN (R 4.1.0)
##  fastmap        1.1.0   2021-01-25 [1] CRAN (R 4.1.0)
##  forcats      * 0.5.1   2021-01-27 [1] CRAN (R 4.1.0)
##  foreign        0.8-81  2020-12-22 [2] CRAN (R 4.1.0)
##  fs             1.5.0   2020-07-31 [1] CRAN (R 4.1.0)
##  generics       0.1.0   2020-10-31 [1] CRAN (R 4.1.0)
##  ggplot2      * 3.3.5   2021-06-25 [1] CRAN (R 4.1.0)
##  gld            2.6.2   2020-01-08 [1] CRAN (R 4.1.0)
##  glue           1.4.2   2020-08-27 [1] CRAN (R 4.1.0)
##  gtable         0.3.0   2019-03-25 [1] CRAN (R 4.1.0)
##  haven          2.4.1   2021-04-23 [1] CRAN (R 4.1.0)
##  highr          0.9     2021-04-16 [1] CRAN (R 4.1.0)
##  hms            1.1.0   2021-05-17 [1] CRAN (R 4.1.0)
##  htmltools      0.5.1.1 2021-01-22 [1] CRAN (R 4.1.0)
##  httr           1.4.2   2020-07-20 [1] CRAN (R 4.1.0)
##  jsonlite       1.7.2   2020-12-09 [1] CRAN (R 4.1.0)
##  knitr        * 1.33    2021-04-24 [1] CRAN (R 4.1.0)
##  lattice        0.20-44 2021-05-02 [2] CRAN (R 4.1.0)
##  libcoin        1.0-8   2021-02-08 [1] CRAN (R 4.1.0)
##  lifecycle      1.0.0   2021-02-15 [1] CRAN (R 4.1.0)
##  lmom           2.8     2019-03-12 [1] CRAN (R 4.1.0)
##  lmtest         0.9-38  2020-09-09 [1] CRAN (R 4.1.0)
##  lubridate      1.7.10  2021-02-26 [1] CRAN (R 4.1.0)
##  magrittr       2.0.1   2020-11-17 [1] CRAN (R 4.1.0)
##  MASS           7.3-54  2021-05-03 [2] CRAN (R 4.1.0)
##  Matrix         1.3-3   2021-05-04 [2] CRAN (R 4.1.0)
##  matrixStats    0.59.0  2021-06-01 [1] CRAN (R 4.1.0)
##  memoise        2.0.0   2021-01-26 [1] CRAN (R 4.1.0)
##  modelr         0.1.8   2020-05-19 [1] CRAN (R 4.1.0)
##  modeltools     0.2-23  2020-03-05 [1] CRAN (R 4.1.0)
##  multcomp       1.4-17  2021-04-29 [1] CRAN (R 4.1.0)
##  multcompView   0.1-8   2019-12-19 [1] CRAN (R 4.1.1)
##  munsell        0.5.0   2018-06-12 [1] CRAN (R 4.1.0)
##  mvtnorm        1.1-2   2021-06-07 [1] CRAN (R 4.1.0)
##  nortest        1.0-4   2015-07-30 [1] CRAN (R 4.1.0)
##  openxlsx       4.2.4   2021-06-16 [1] CRAN (R 4.1.0)
##  pillar         1.6.1   2021-05-16 [1] CRAN (R 4.1.0)
##  pkgbuild       1.2.0   2020-12-15 [1] CRAN (R 4.1.0)
##  pkgconfig      2.0.3   2019-09-22 [1] CRAN (R 4.1.0)
##  pkgload        1.2.1   2021-04-06 [1] CRAN (R 4.1.0)
##  plyr           1.8.6   2020-03-03 [1] CRAN (R 4.1.0)
##  prettyunits    1.1.1   2020-01-24 [1] CRAN (R 4.1.0)
##  processx       3.5.2   2021-04-30 [1] CRAN (R 4.1.0)
##  proxy          0.4-26  2021-06-07 [1] CRAN (R 4.1.0)
##  ps             1.6.0   2021-02-28 [1] CRAN (R 4.1.0)
##  purrr        * 0.3.4   2020-04-17 [1] CRAN (R 4.1.0)
##  R6             2.5.0   2020-10-28 [1] CRAN (R 4.1.0)
##  rcompanion   * 2.4.1   2021-05-18 [1] CRAN (R 4.1.0)
##  Rcpp           1.0.6   2021-01-15 [1] CRAN (R 4.1.0)
##  readr        * 1.4.0   2020-10-05 [1] CRAN (R 4.1.0)
##  readxl         1.3.1   2019-03-13 [1] CRAN (R 4.1.0)
##  remotes        2.4.0   2021-06-02 [1] CRAN (R 4.1.0)
##  reprex         2.0.0   2021-04-02 [1] CRAN (R 4.1.0)
##  rio            0.5.27  2021-06-21 [1] CRAN (R 4.1.0)
##  rlang          0.4.11  2021-04-30 [1] CRAN (R 4.1.0)
##  rmarkdown      2.9     2021-06-15 [1] CRAN (R 4.1.0)
##  rootSolve      1.8.2.1 2020-04-27 [1] CRAN (R 4.1.0)
##  rprojroot      2.0.2   2020-11-15 [1] CRAN (R 4.1.0)
##  rstatix      * 0.7.0   2021-02-13 [1] CRAN (R 4.1.0)
##  rstudioapi     0.13    2020-11-12 [1] CRAN (R 4.1.0)
##  rvest          1.0.0   2021-03-09 [1] CRAN (R 4.1.0)
##  sandwich       3.0-1   2021-05-18 [1] CRAN (R 4.1.0)
##  scales         1.1.1   2020-05-11 [1] CRAN (R 4.1.0)
##  sessioninfo    1.1.1   2018-11-05 [1] CRAN (R 4.1.0)
##  stringi        1.6.1   2021-05-10 [1] CRAN (R 4.1.0)
##  stringr      * 1.4.0   2019-02-10 [1] CRAN (R 4.1.0)
##  survival       3.2-11  2021-04-26 [2] CRAN (R 4.1.0)
##  testthat       3.0.3   2021-06-16 [1] CRAN (R 4.1.0)
##  TH.data        1.0-10  2019-01-21 [1] CRAN (R 4.1.0)
##  tibble       * 3.1.2   2021-05-16 [1] CRAN (R 4.1.0)
##  tidyr        * 1.1.3   2021-03-03 [1] CRAN (R 4.1.0)
##  tidyselect     1.1.1   2021-04-30 [1] CRAN (R 4.1.0)
##  tidyverse    * 1.3.1   2021-04-15 [1] CRAN (R 4.1.0)
##  usethis      * 2.0.1   2021-02-10 [1] CRAN (R 4.1.0)
##  utf8           1.2.1   2021-03-12 [1] CRAN (R 4.1.0)
##  vctrs          0.3.8   2021-04-29 [1] CRAN (R 4.1.0)
##  withr          2.4.2   2021-04-18 [1] CRAN (R 4.1.0)
##  xfun           0.24    2021-06-15 [1] CRAN (R 4.1.0)
##  xml2           1.3.2   2020-04-23 [1] CRAN (R 4.1.0)
##  yaml           2.2.1   2020-02-01 [1] CRAN (R 4.1.0)
##  zip            2.2.0   2021-05-31 [1] CRAN (R 4.1.0)
##  zoo            1.8-9   2021-03-09 [1] CRAN (R 4.1.0)
## 
## [1] C:/Users/Ben Field/Documents/R/win-library/4.1
## [2] C:/Program Files/R/R-4.1.0/library
```

## 5. Citations

1. Kassambara, Alboukadel. 2021. Rstatix: Pipe-Friendly Framework for Basic Statistical Tests. https://CRAN.R-project.org/package=rstatix.
2. Mangiafico, Salvatore. 2021. Rcompanion: Functions to Support Extension Education Program Evaluation. https://CRAN.R-project.org/package=rcompanion.
3. R Core Team. 2020. R: A Language and Environment for Statistical Computing. Vienna, Austria: R Foundation for Statistical Computing. https://www.R-project.org/.
4. Wickham et al., (2019). Welcome to the tidyverse. Journal of Open Source Software, 4(43), 1686, https://doi.org/10.21105/joss.01686
5. Wickham, Hadley, Hester, Jim and Chang, Winston (2021). devtools: Tools to Make Developing R Packages Easier. R package version 2.4.2. https://CRAN.R-project.org/package=devtools
6. Yihui Xie (2021). knitr: A General-Purpose Package for Dynamic Report Generation in R. R package version 1.33.
