# Supplementary figures and images for: A guanosine tetraphosphate (ppGpp) mediated brake on photosynthesis is required for acclimation to nitrogen limitation in Arabidopsis
Source: eLife. 2022 Feb 14;11:e75041. doi: 10.7554/eLife.75041 (PMC8887892; doi:10.7554/eLife.75041)

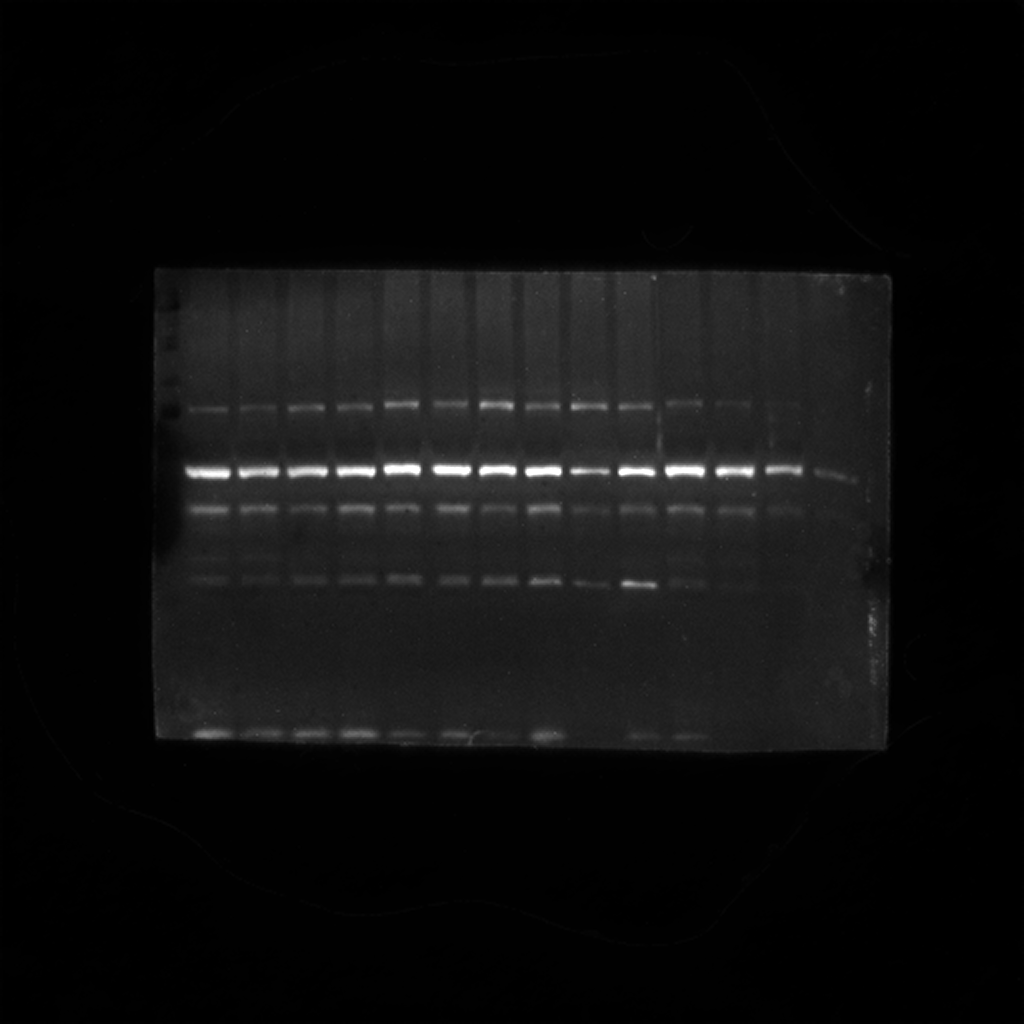

Supplement: Figure 3—source data 1. [file elife-75041-fig3-data1.zip › Fig 3 source data 1/ptox.Tif]

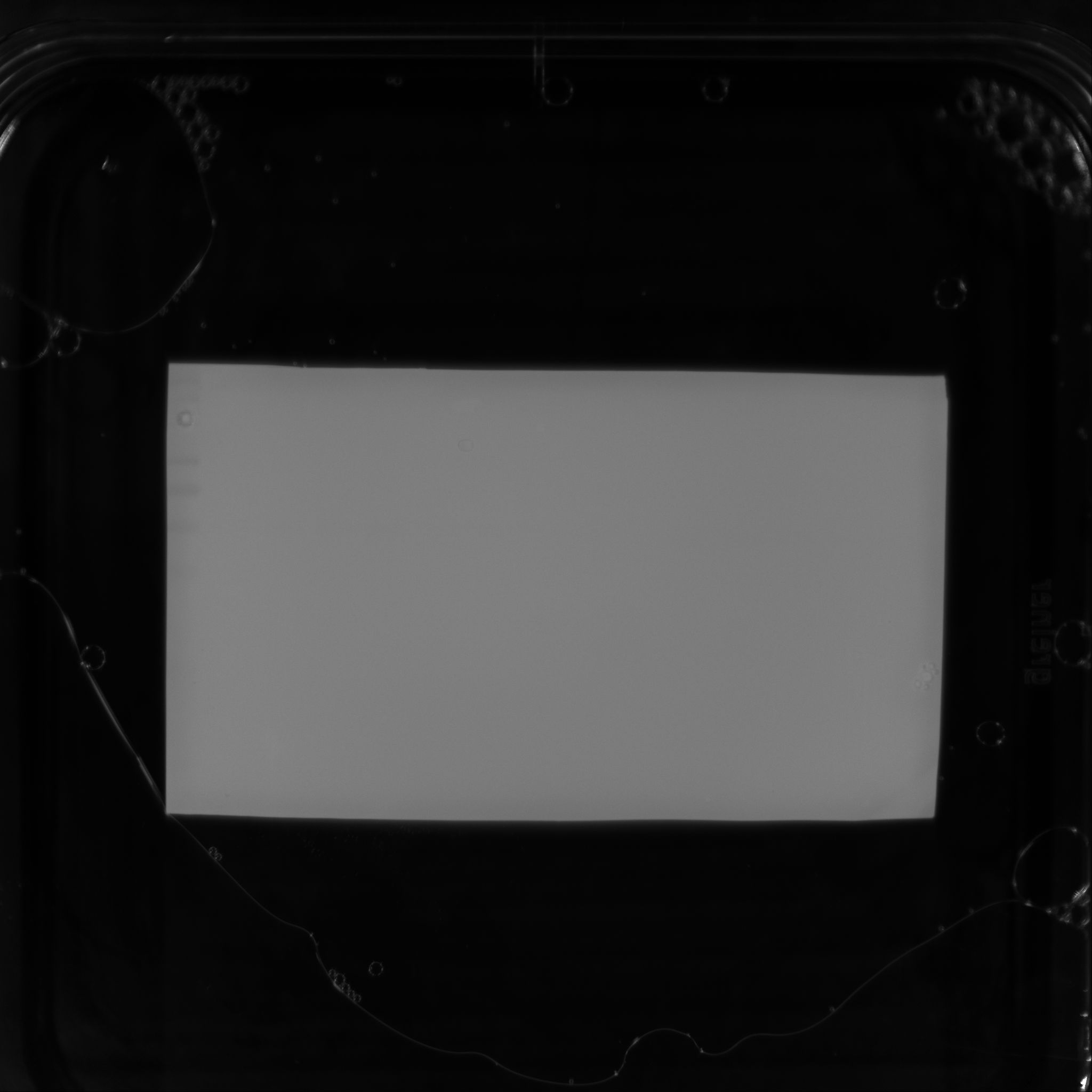

Supplement: Figure 3—source data 1. [file elife-75041-fig3-data1.zip › Fig 3 source data 1/PBA1_epi_light.jpg]

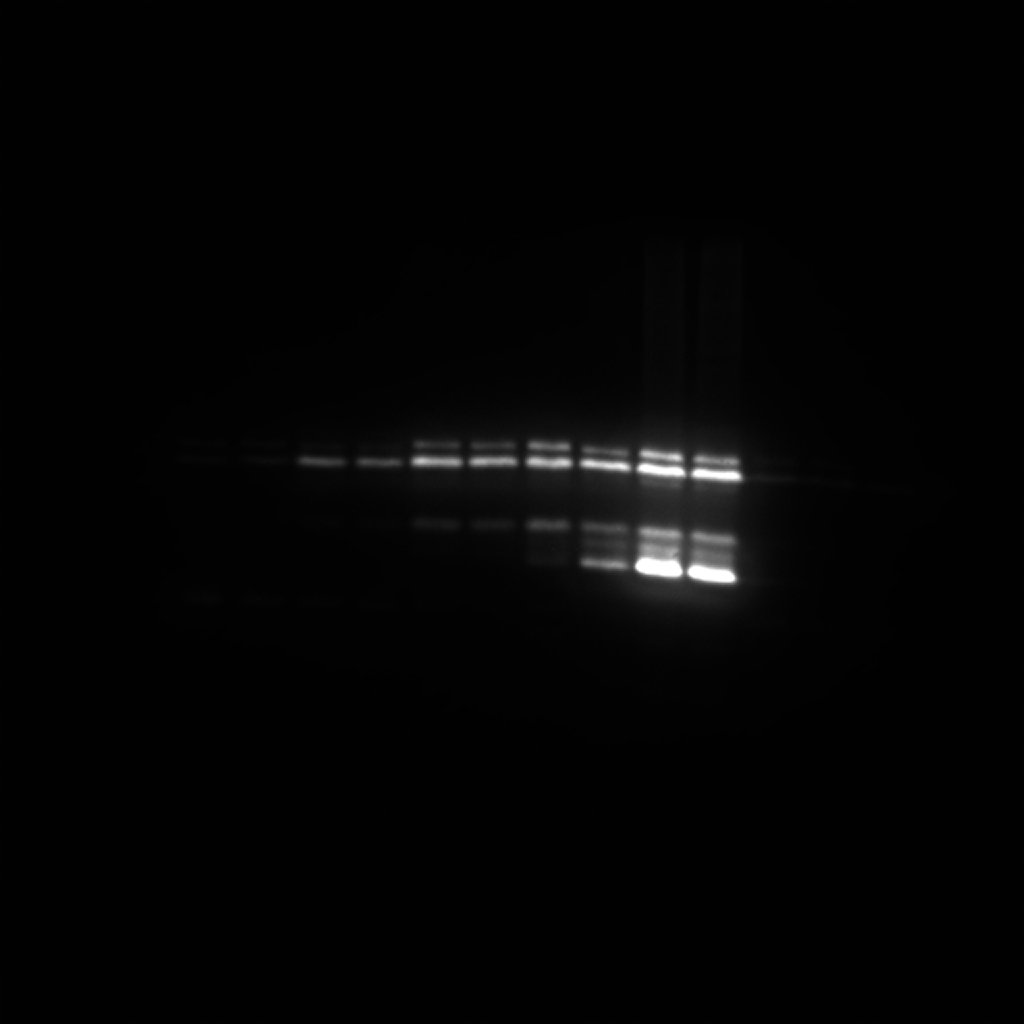

Supplement: Figure 3—source data 1. [file elife-75041-fig3-data1.zip › Fig 3 source data 1/SAG12.Tif]

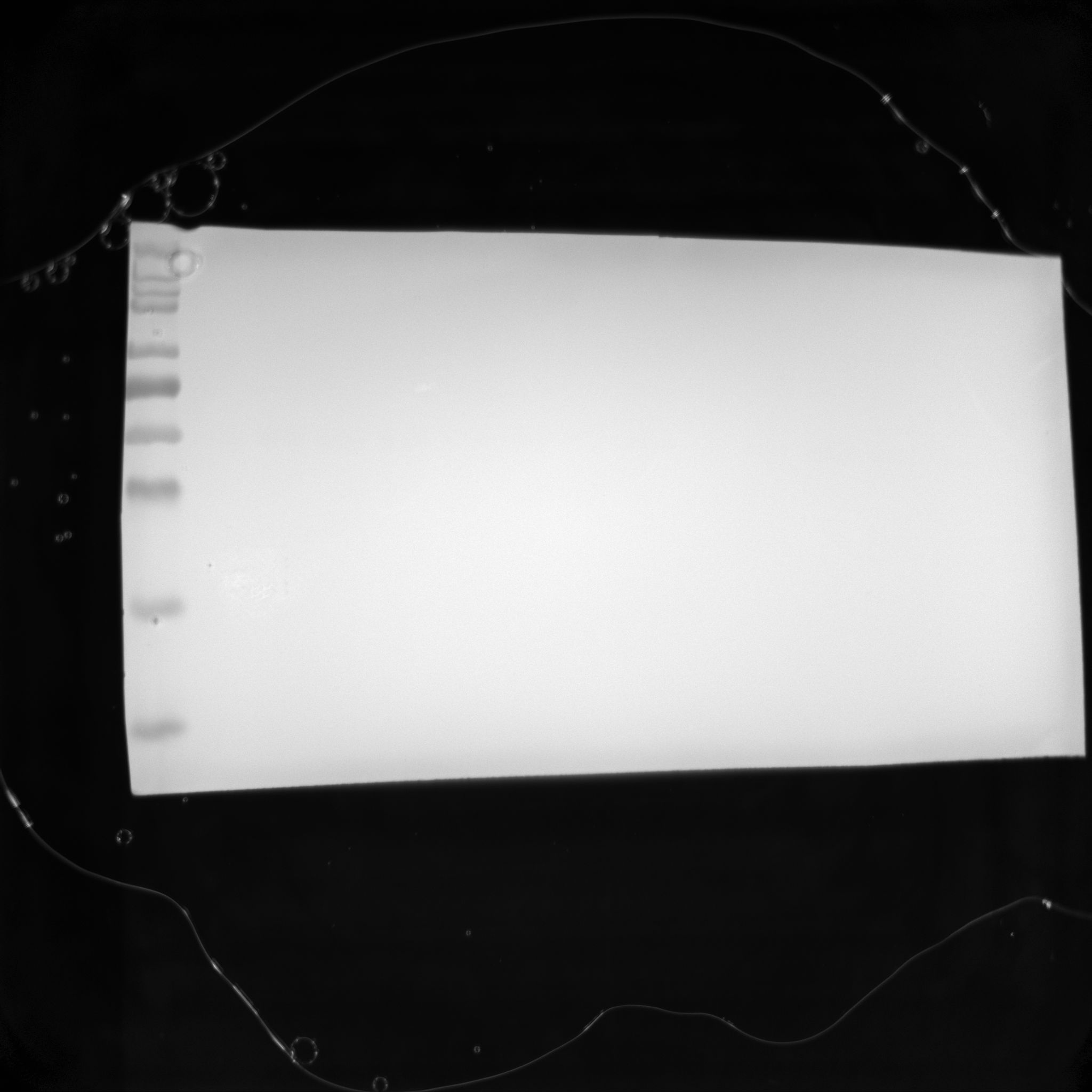

Supplement: Figure 3—source data 1. [file elife-75041-fig3-data1.zip › Fig 3 source data 1/SAG12_epi_light.jpg]

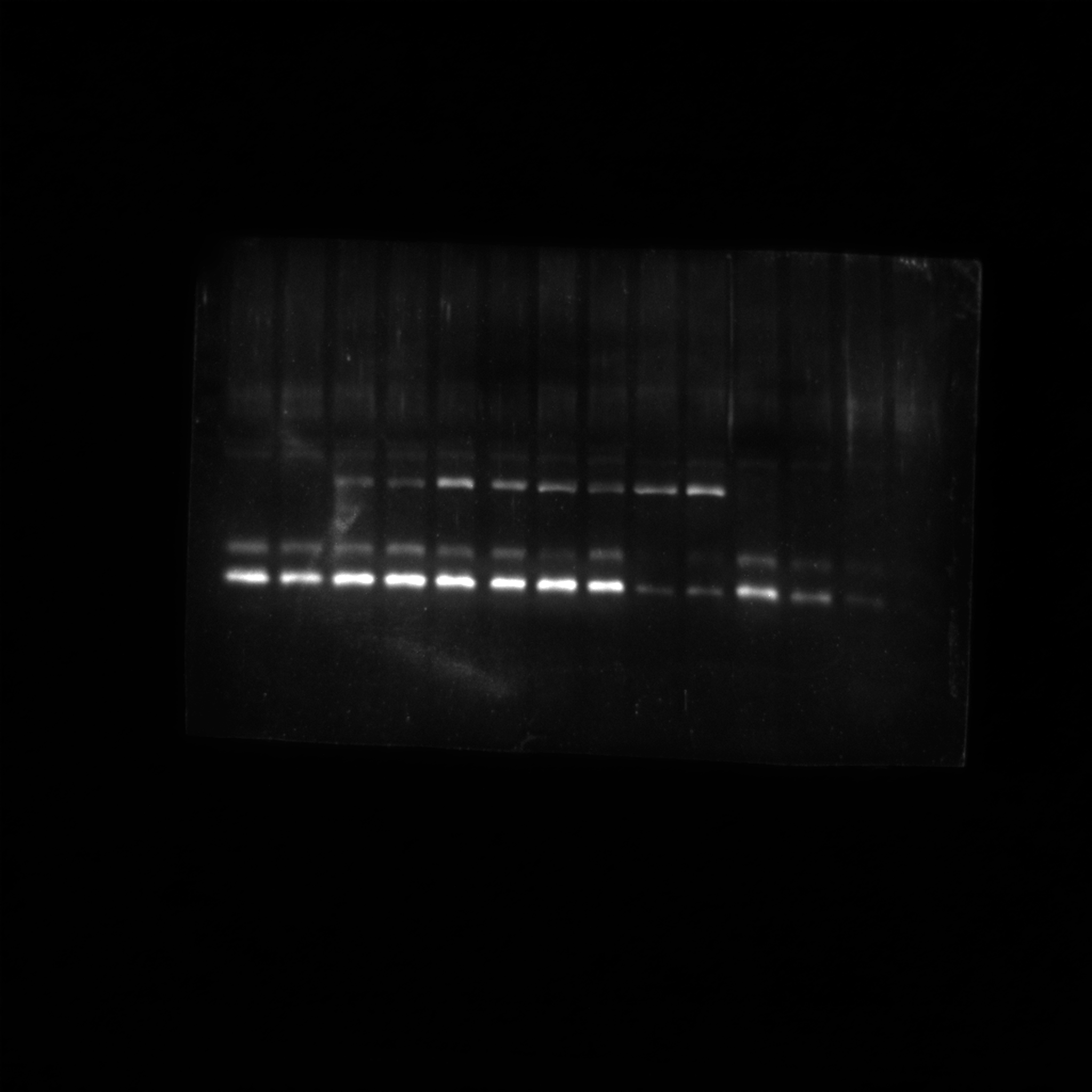

Supplement: Figure 3—source data 1. [file elife-75041-fig3-data1.zip › Fig 3 source data 1/lhcb1.Tif]

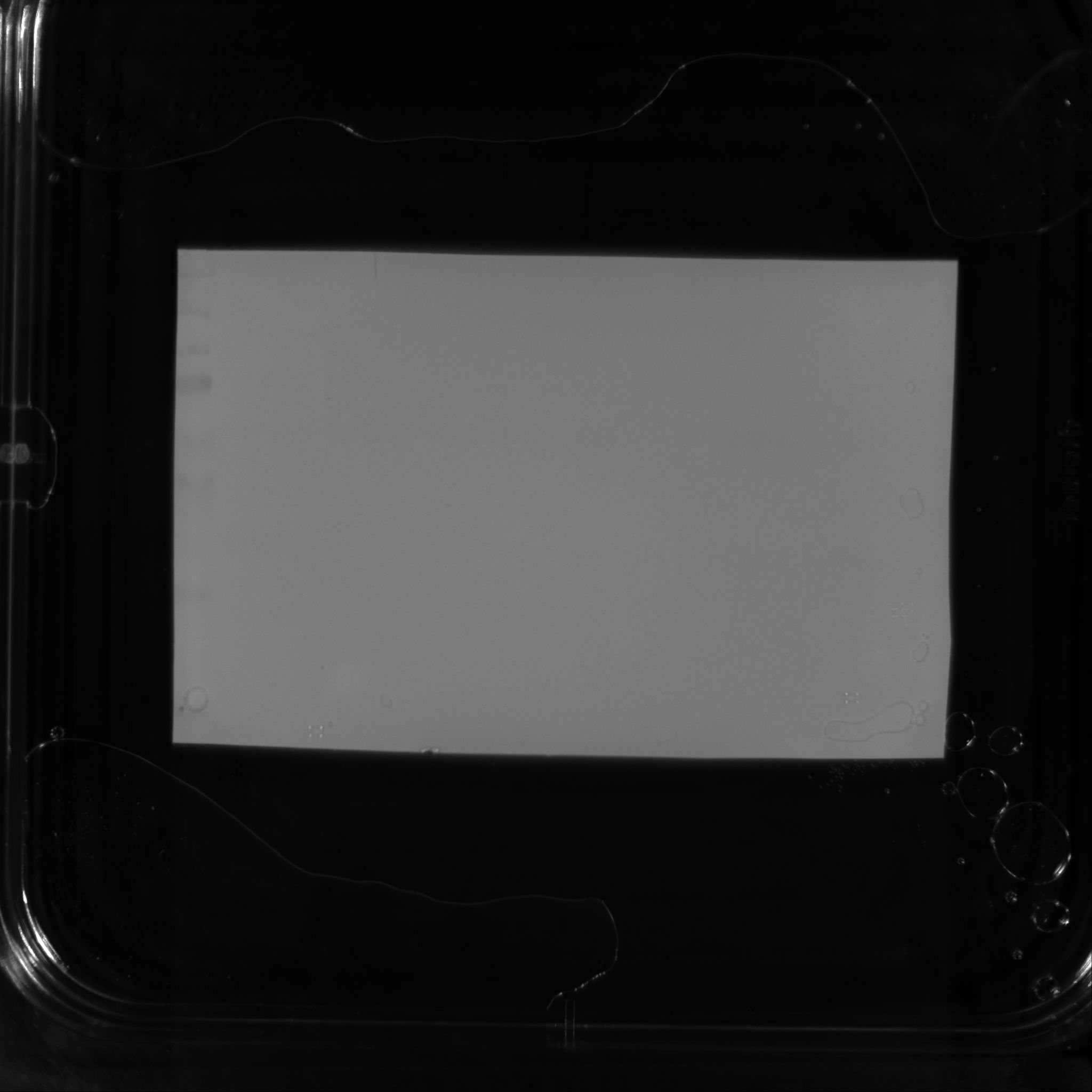

Supplement: Figure 3—source data 1. [file elife-75041-fig3-data1.zip › Fig 3 source data 1/psbO_epi_light.jpg]

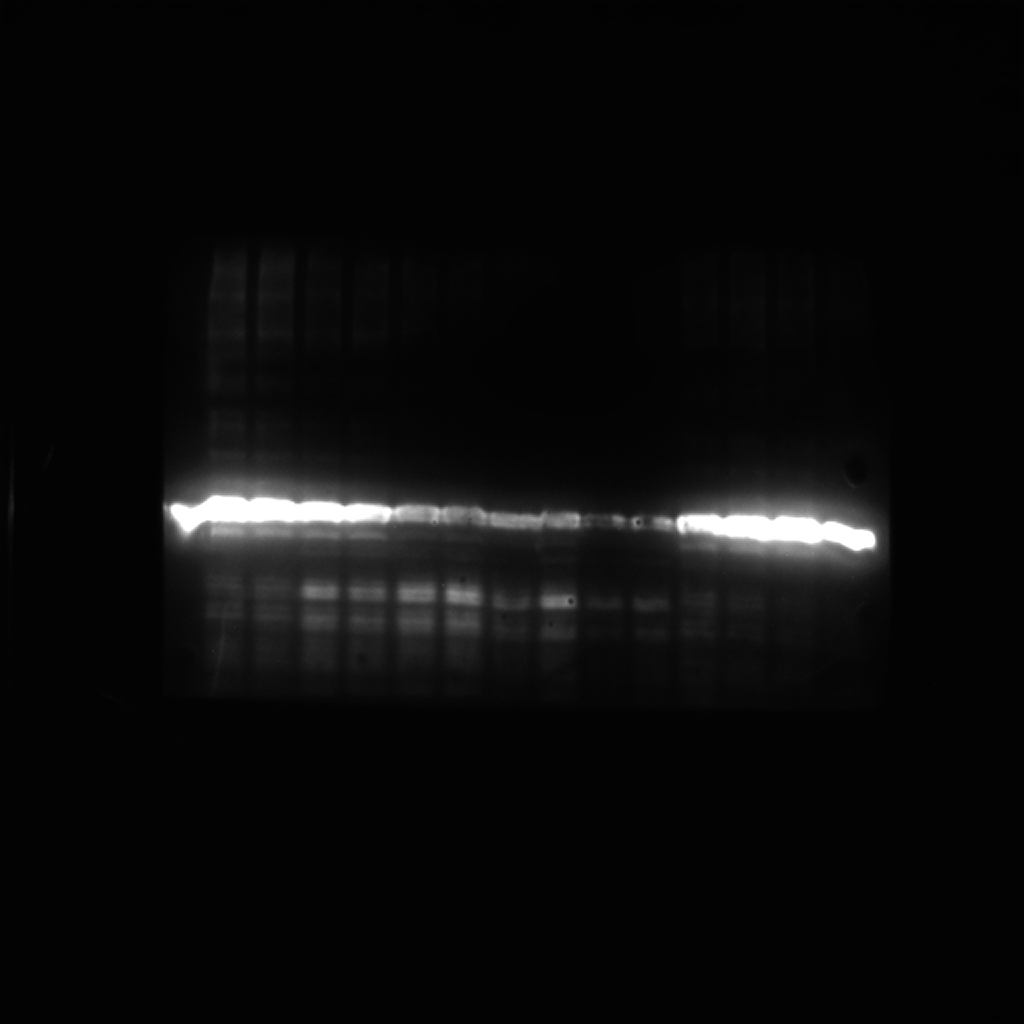

Supplement: Figure 3—source data 1. [file elife-75041-fig3-data1.zip › Fig 3 source data 1/psbO.Tif]

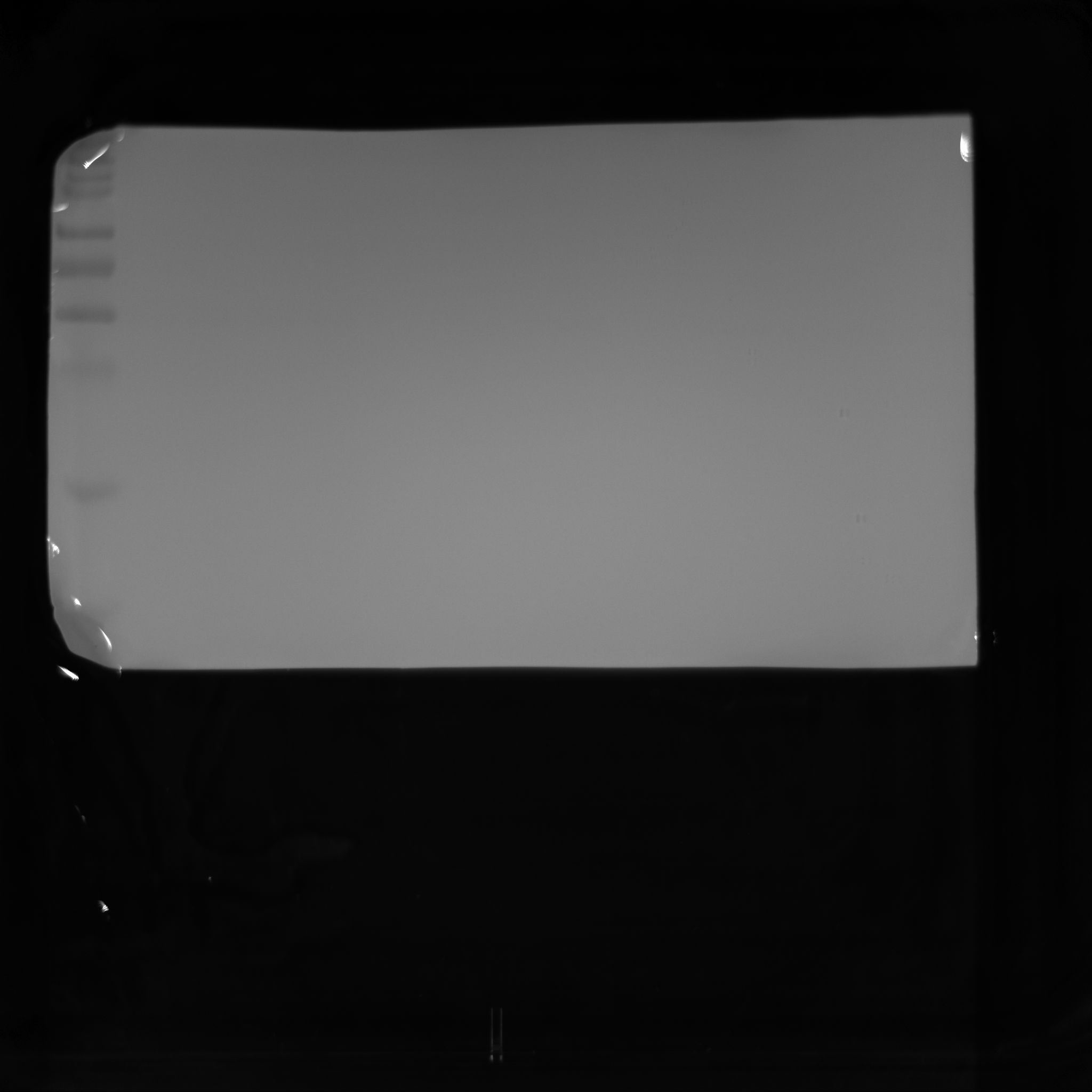

Supplement: Figure 3—source data 1. [file elife-75041-fig3-data1.zip › Fig 3 source data 1/psbA_epi_light.jpg]

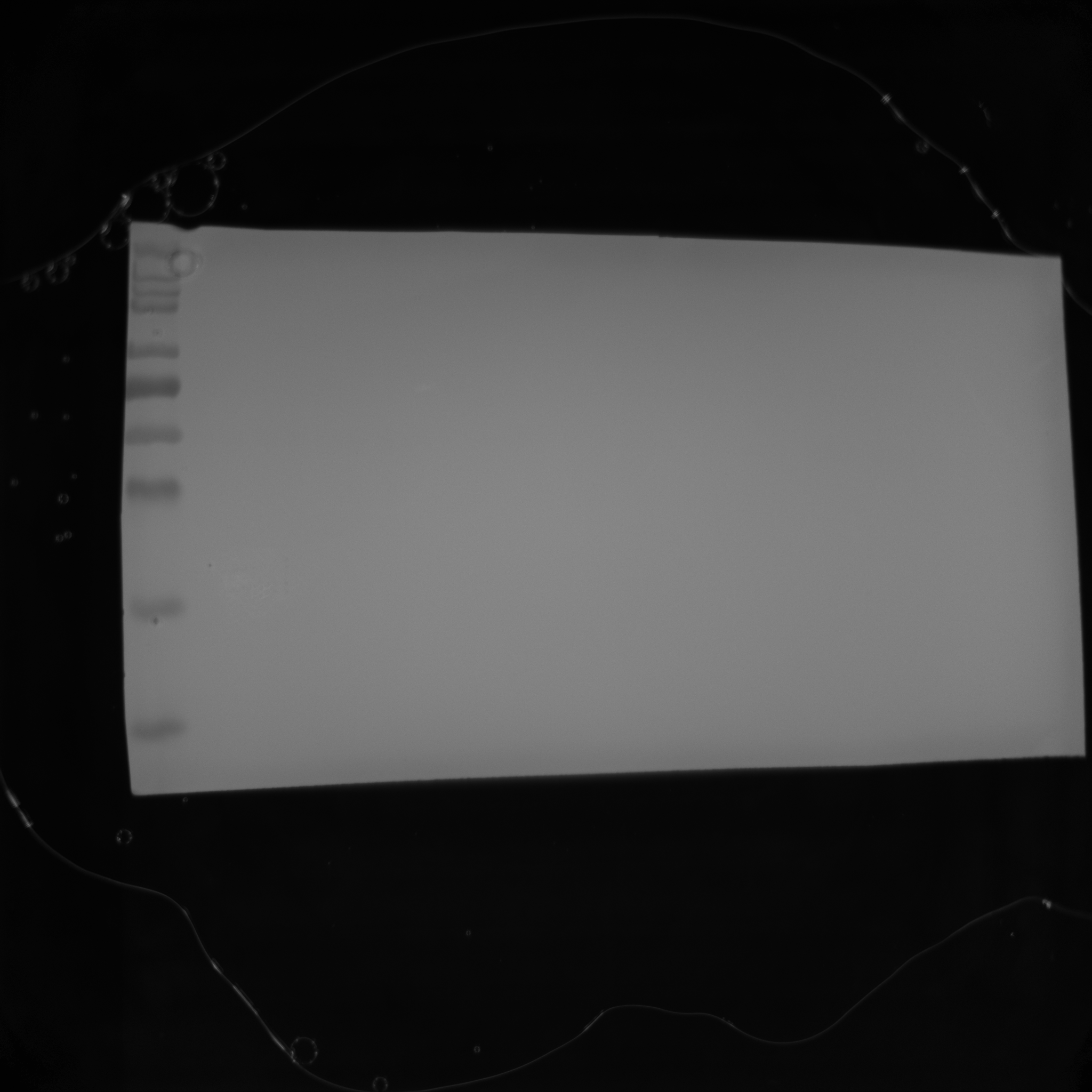

Supplement: Figure 3—source data 1. [file elife-75041-fig3-data1.zip › Fig 3 source data 1/SAG12_epi_light.Tif]

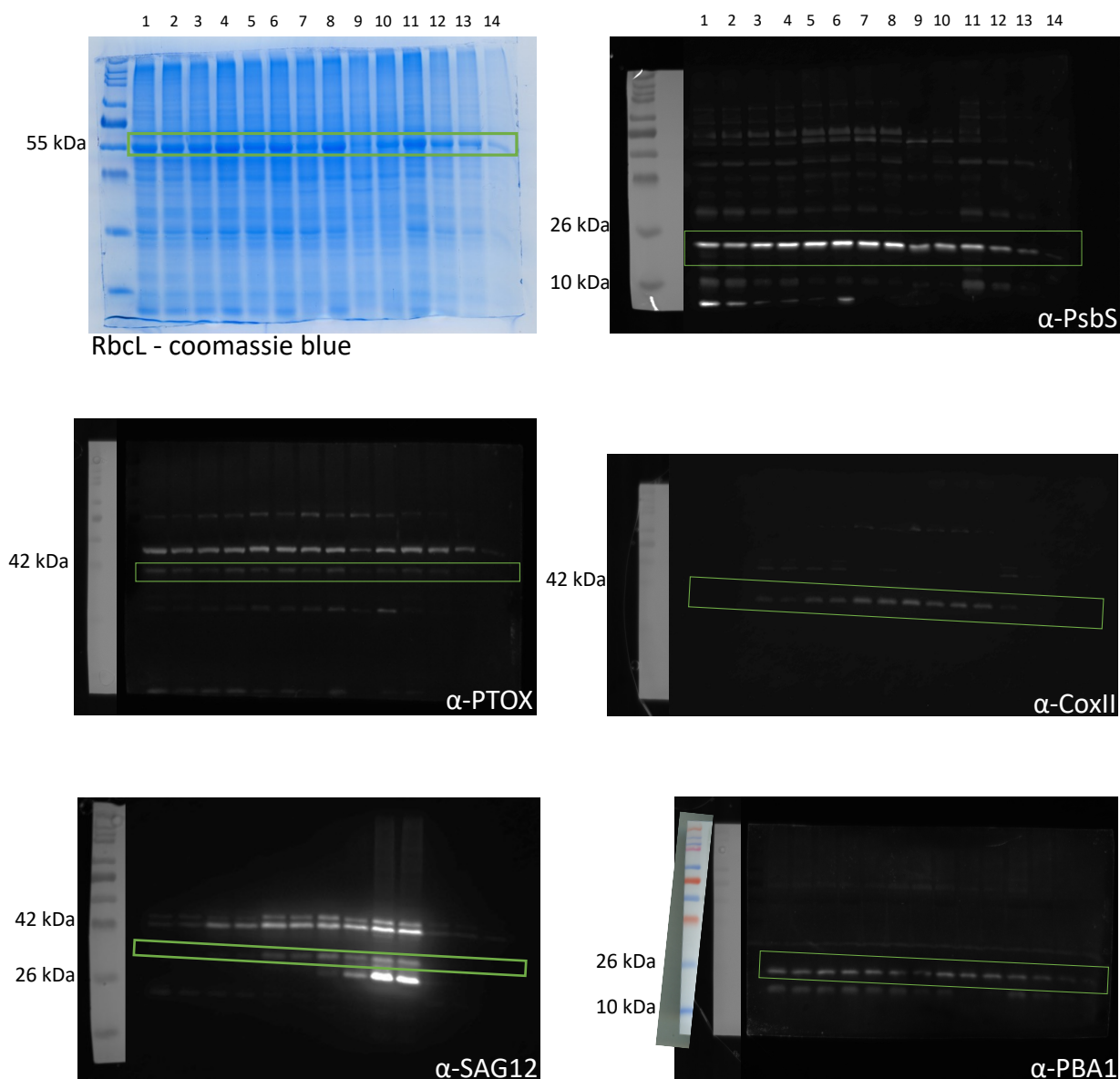

Supplement: Figure 3—source data 1. [file elife-75041-fig3-data1.zip › Fig 3 source data 1/fig3A_source_data_summary.pdf]

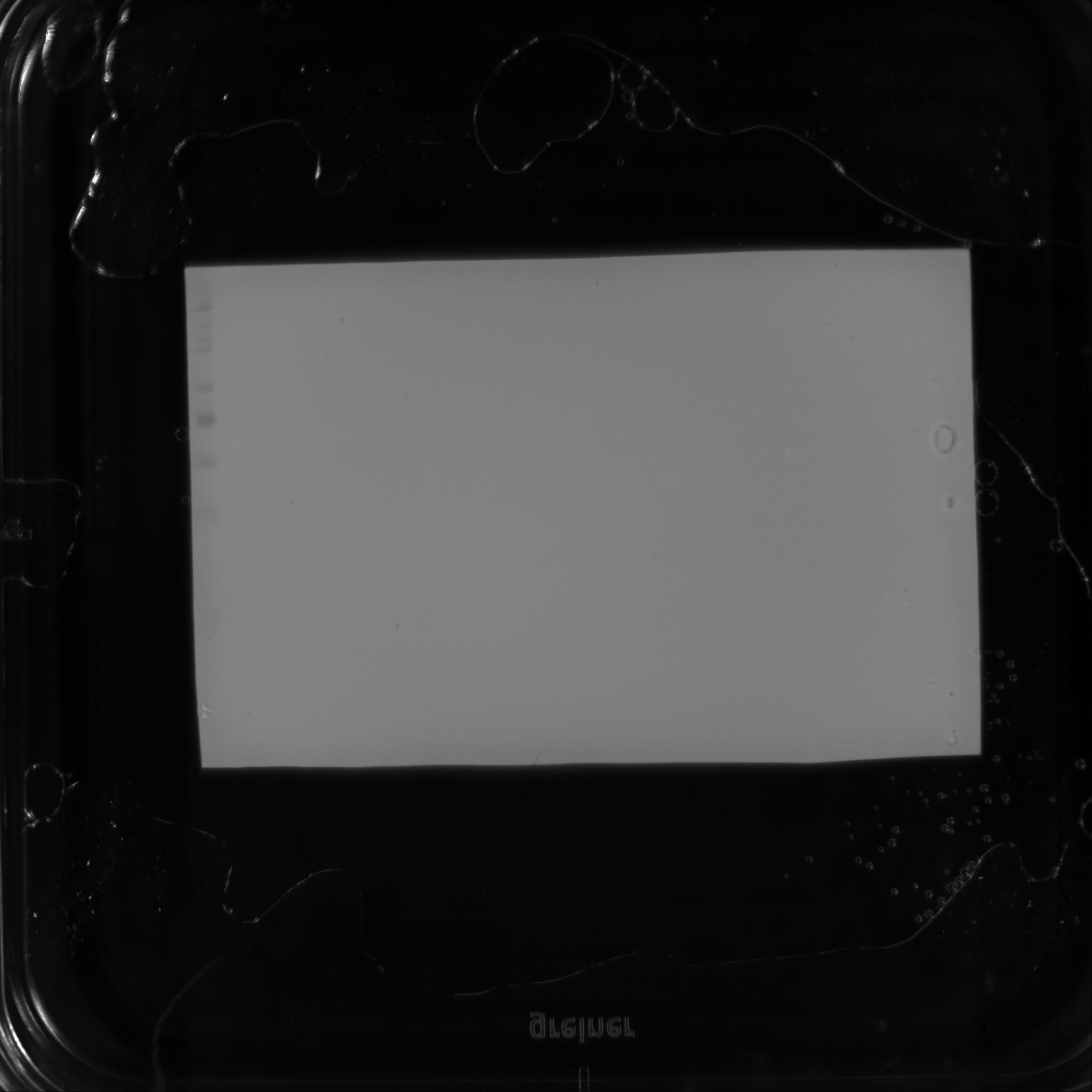

Supplement: Figure 3—source data 1. [file elife-75041-fig3-data1.zip › Fig 3 source data 1/petA_epi_light.jpg]

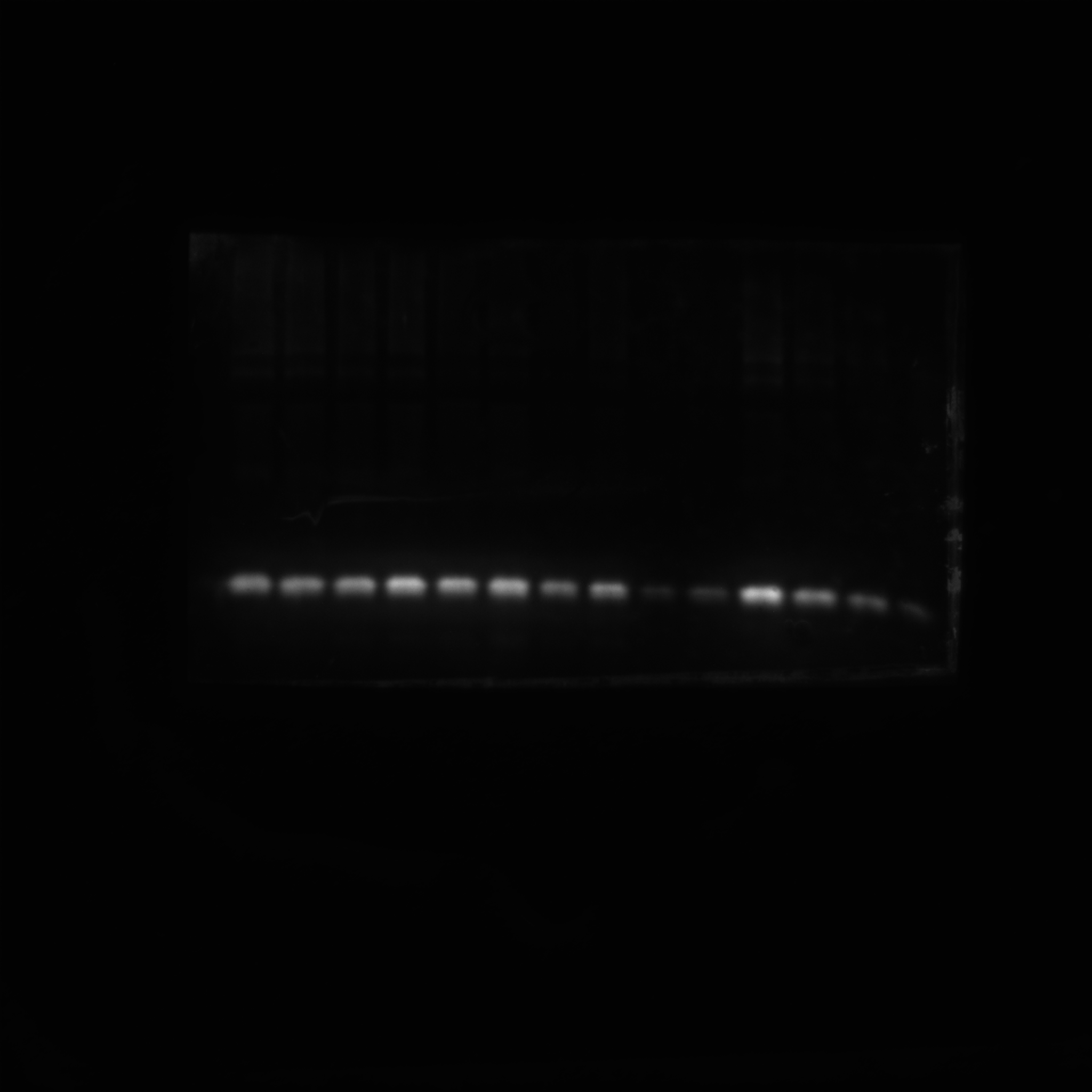

Supplement: Figure 3—source data 1. [file elife-75041-fig3-data1.zip › Fig 3 source data 1/psaD.Tif]

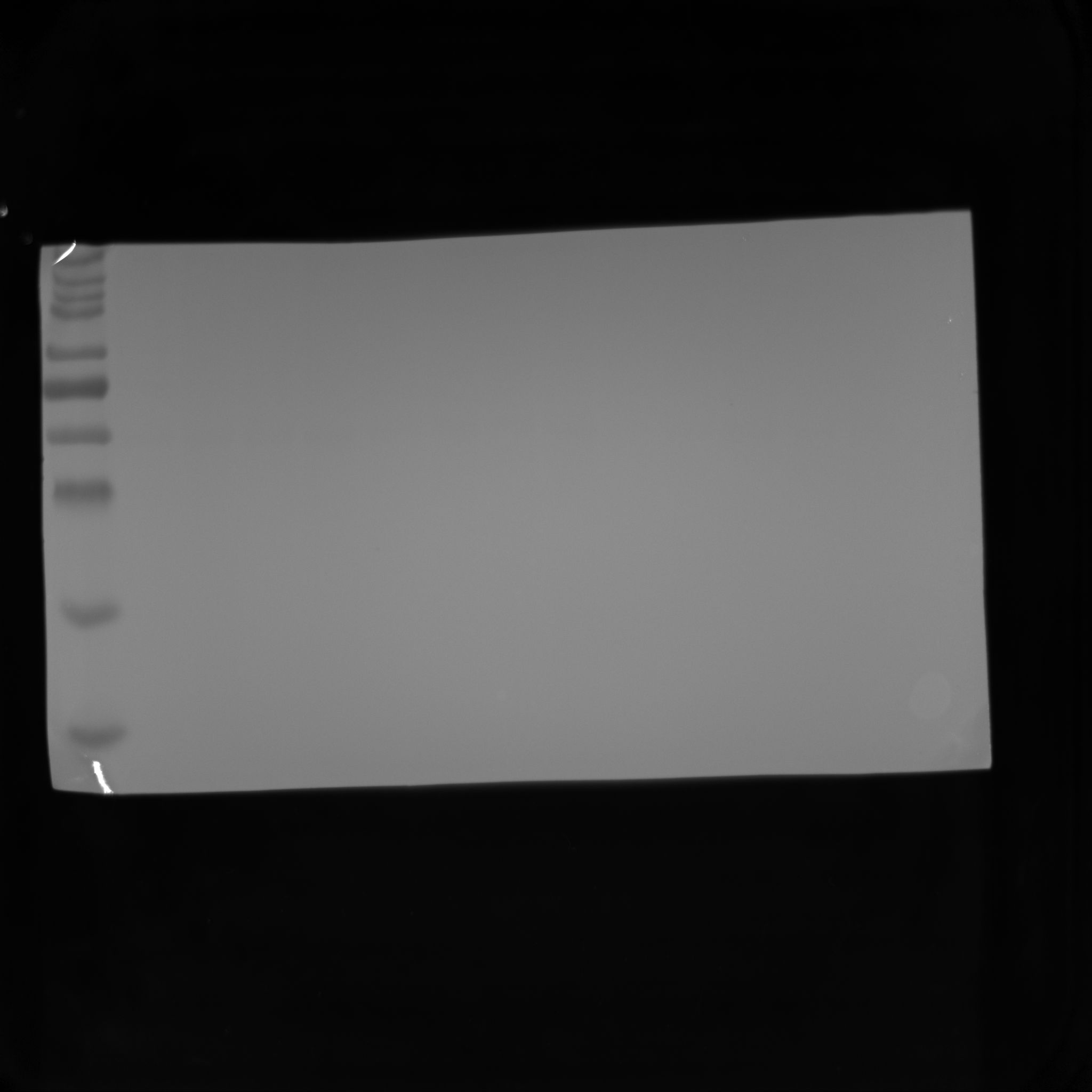

Supplement: Figure 3—source data 1. [file elife-75041-fig3-data1.zip › Fig 3 source data 1/psbS_epi_light.jpg]

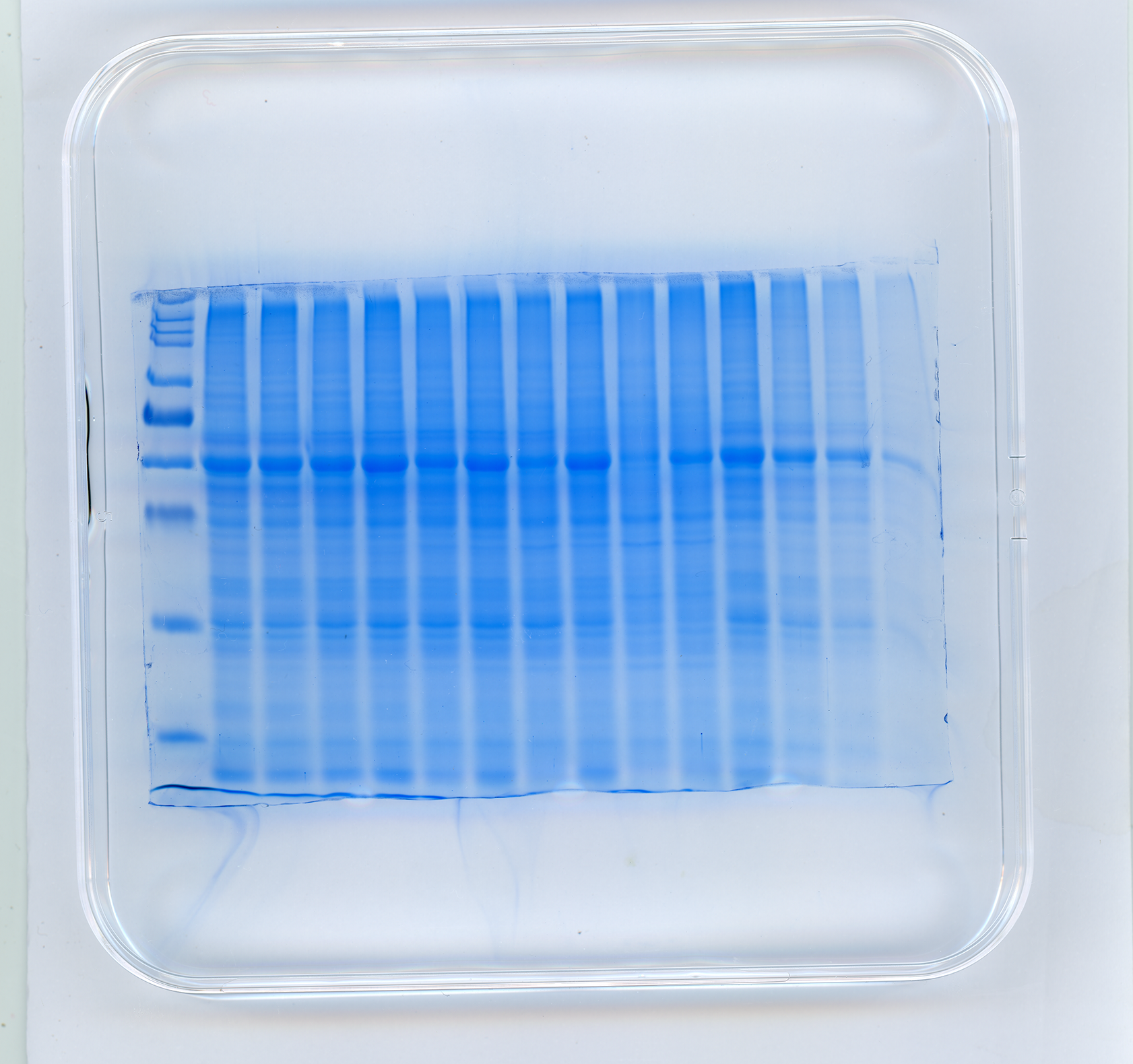

Supplement: Figure 3—source data 1. [file elife-75041-fig3-data1.zip › Fig 3 source data 1/coomassie_blue_timecourse.tif]

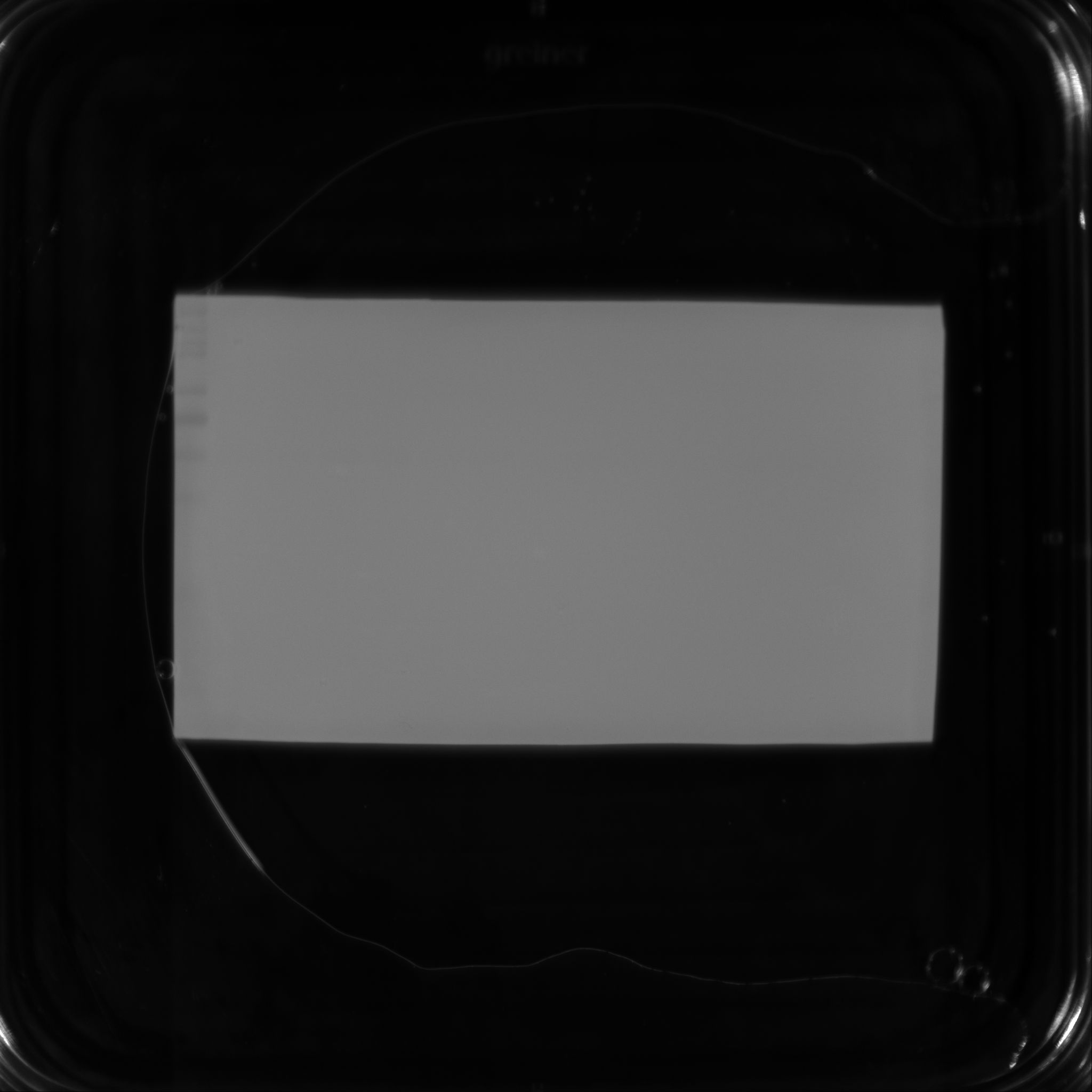

Supplement: Figure 3—source data 1. [file elife-75041-fig3-data1.zip › Fig 3 source data 1/coxII_epi_light.jpg]

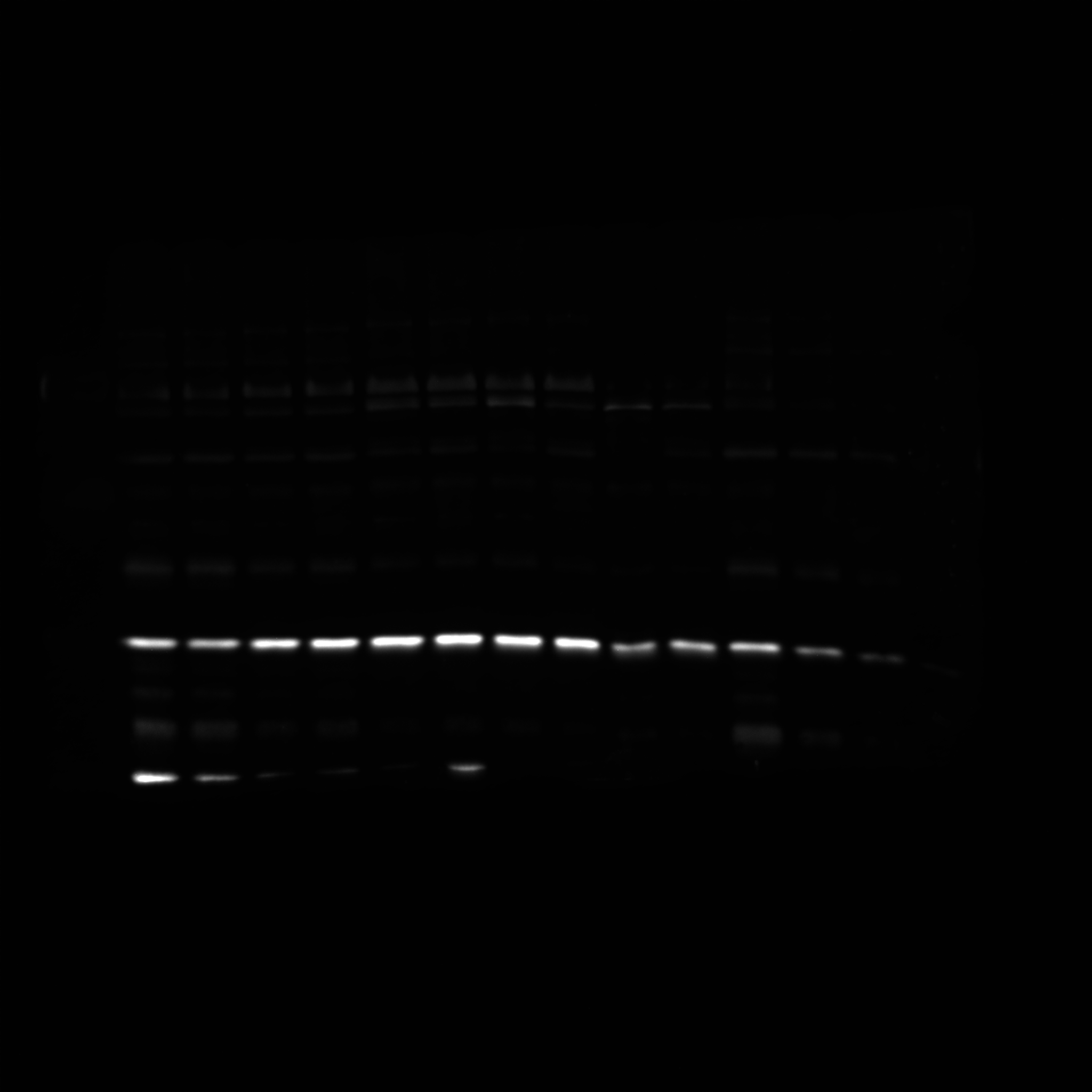

Supplement: Figure 3—source data 1. [file elife-75041-fig3-data1.zip › Fig 3 source data 1/psbS.Tif]

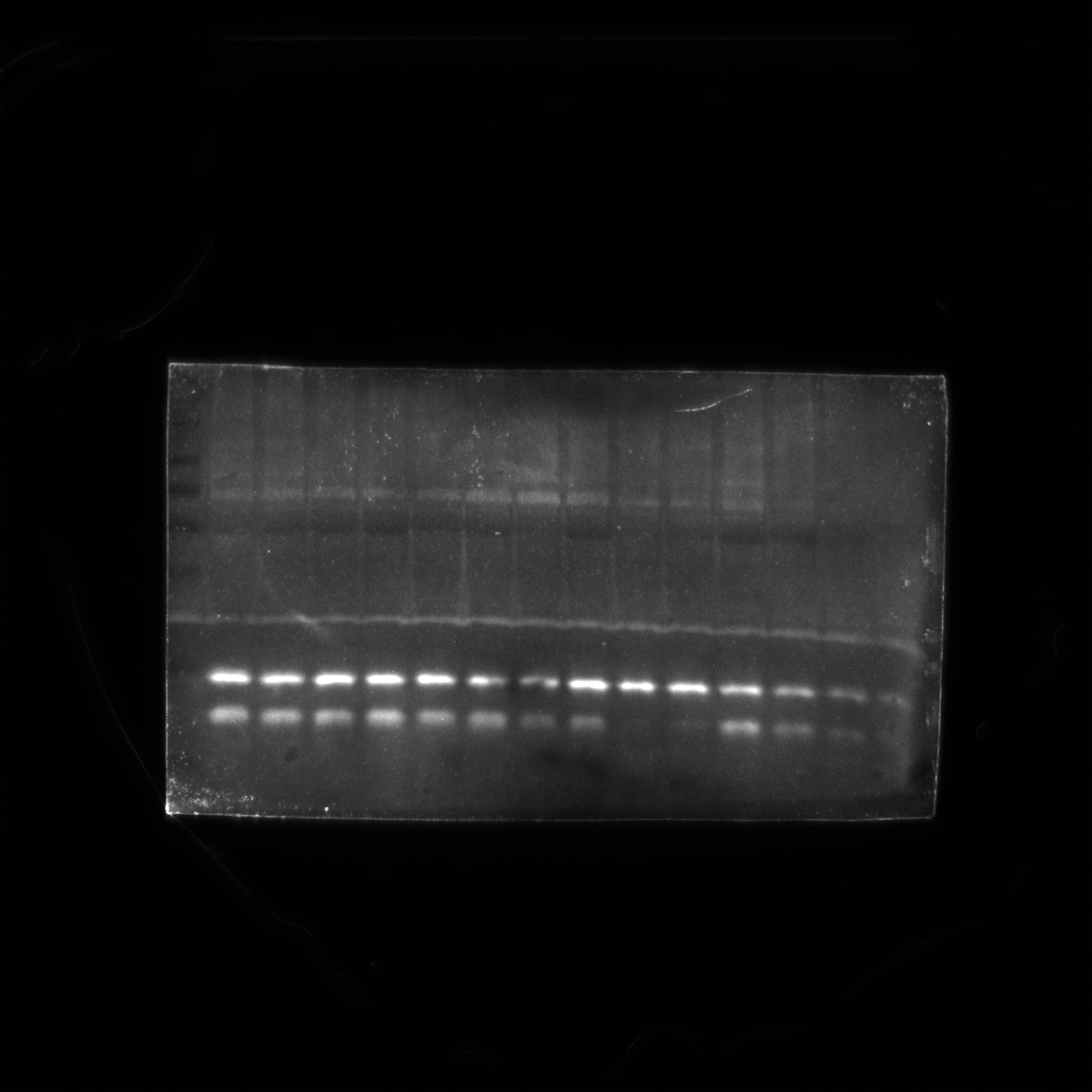

Supplement: Figure 3—source data 1. [file elife-75041-fig3-data1.zip › Fig 3 source data 1/PBA1.Tif]

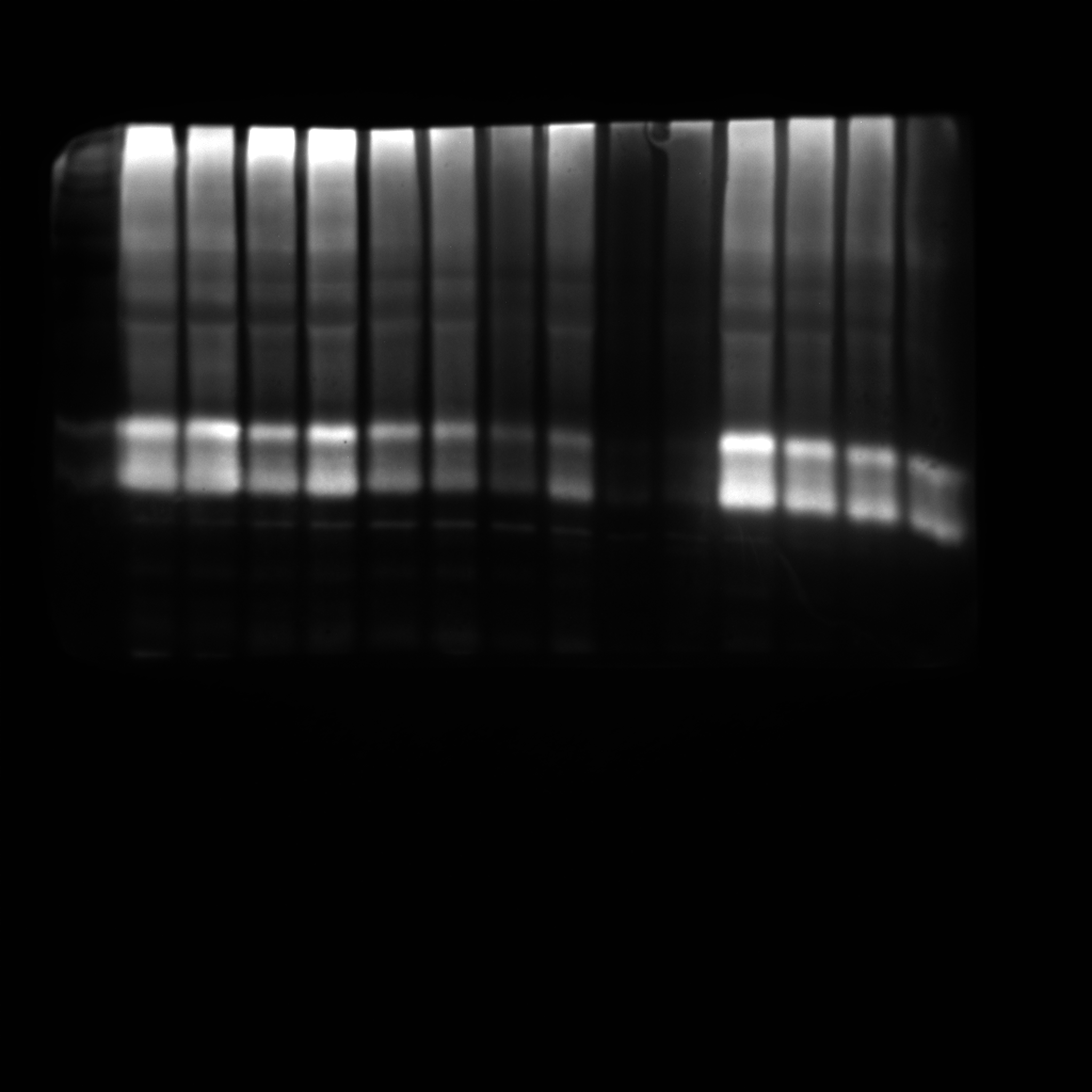

Supplement: Figure 3—source data 1. [file elife-75041-fig3-data1.zip › Fig 3 source data 1/psbA.Tif]

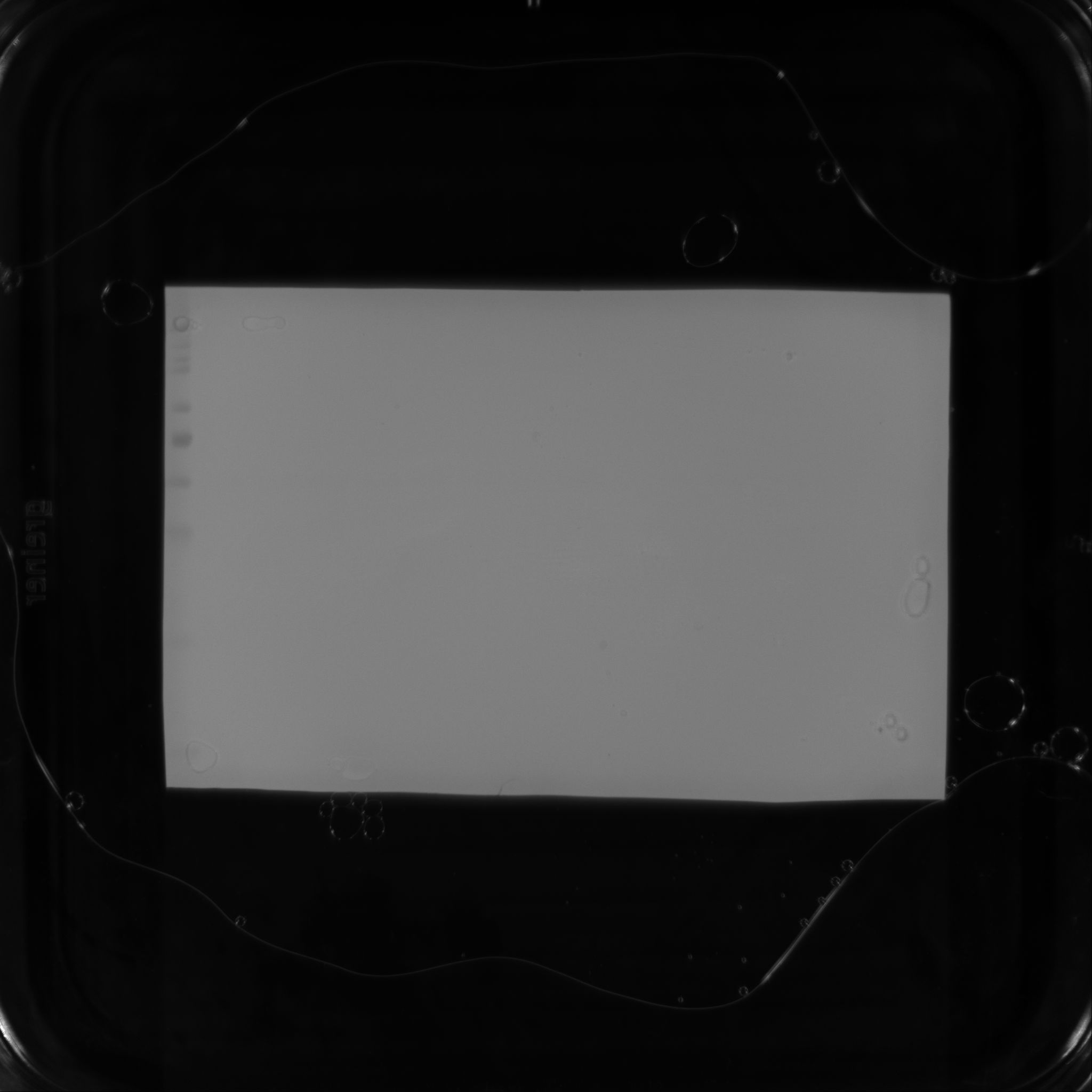

Supplement: Figure 3—source data 1. [file elife-75041-fig3-data1.zip › Fig 3 source data 1/ptox_epi_light.jpg]

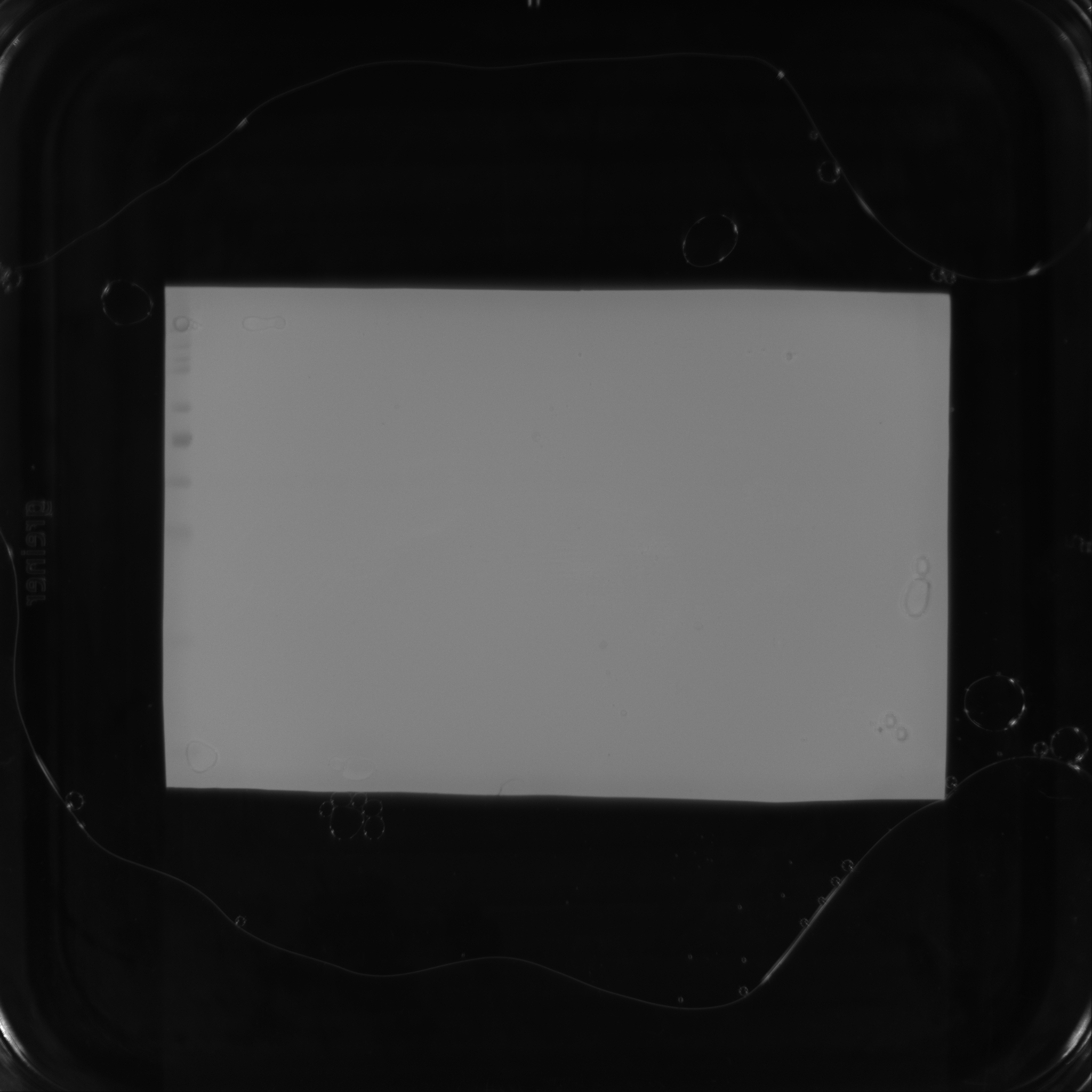

Supplement: Figure 3—source data 1. [file elife-75041-fig3-data1.zip › Fig 3 source data 1/ptox_epi_light.Tif]

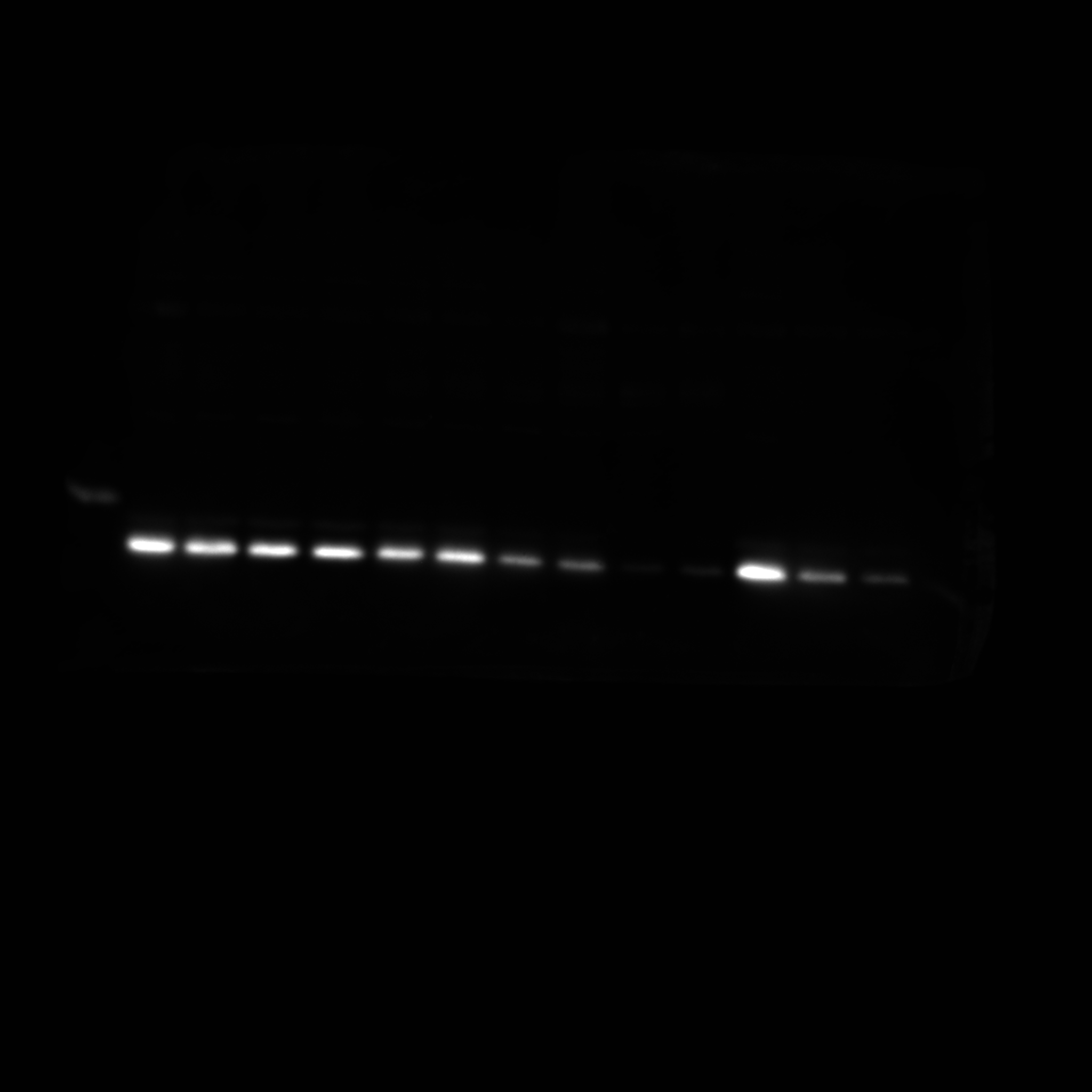

Supplement: Figure 3—source data 1. [file elife-75041-fig3-data1.zip › Fig 3 source data 1/lhca1.Tif]

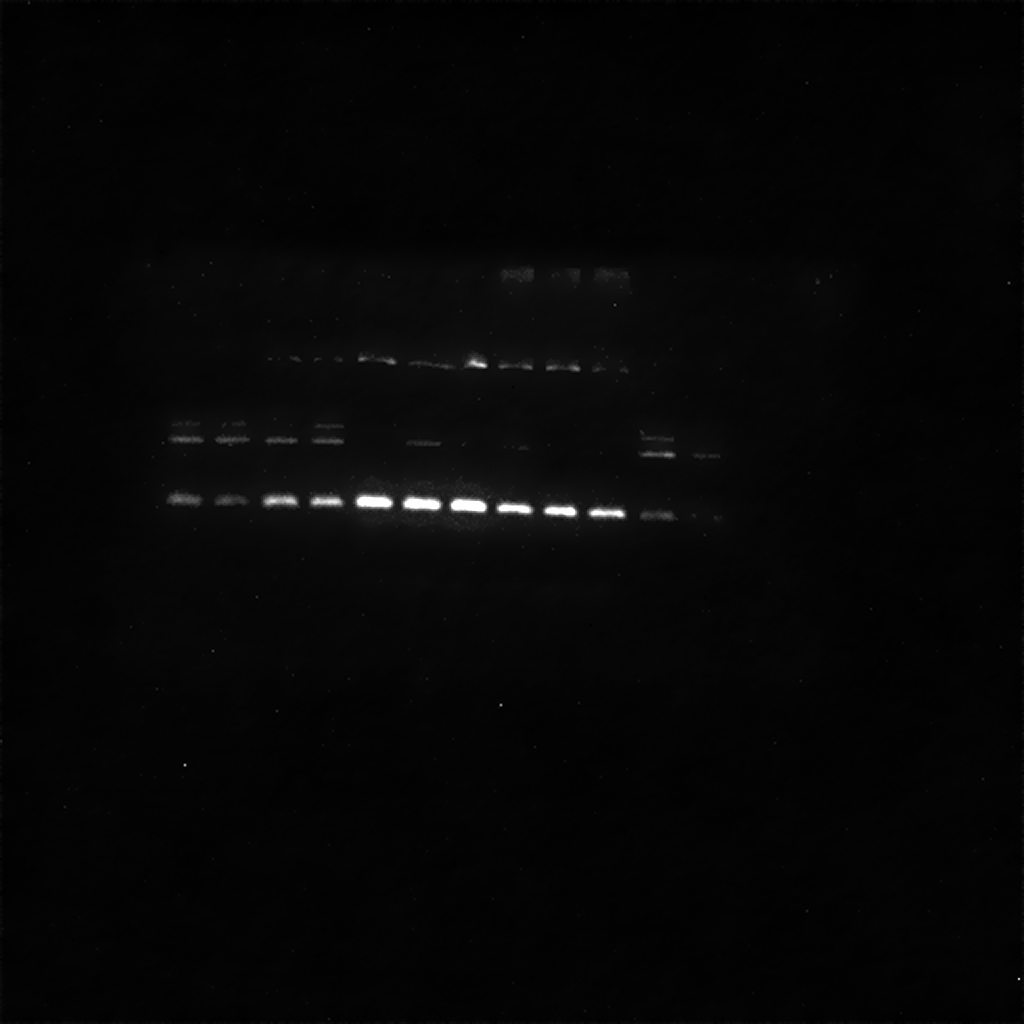

Supplement: Figure 3—source data 1. [file elife-75041-fig3-data1.zip › Fig 3 source data 1/coxII.Tif]

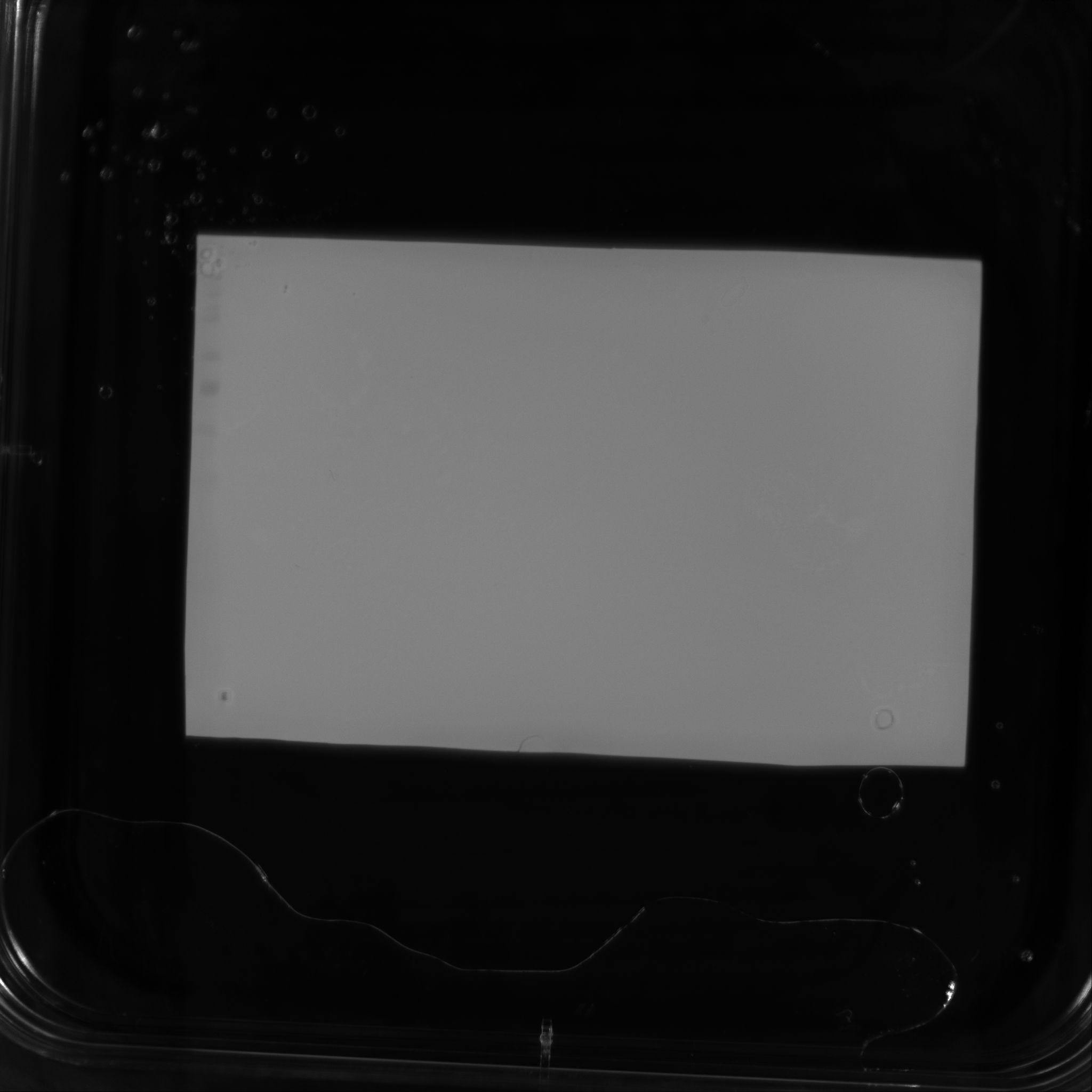

Supplement: Figure 3—source data 1. [file elife-75041-fig3-data1.zip › Fig 3 source data 1/lhcb1_epi_light.jpg]

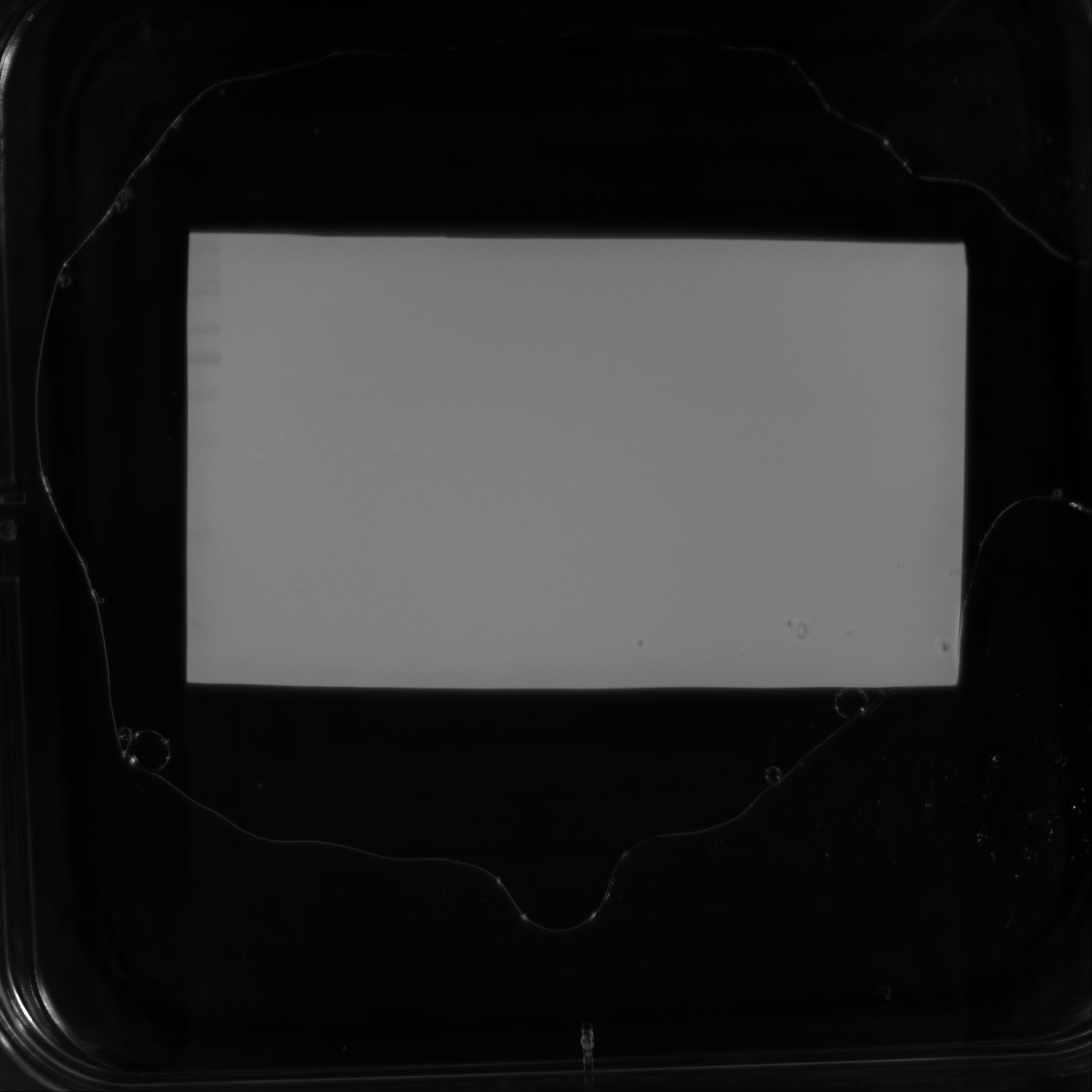

Supplement: Figure 3—source data 1. [file elife-75041-fig3-data1.zip › Fig 3 source data 1/psaD_epi_light.jpg]

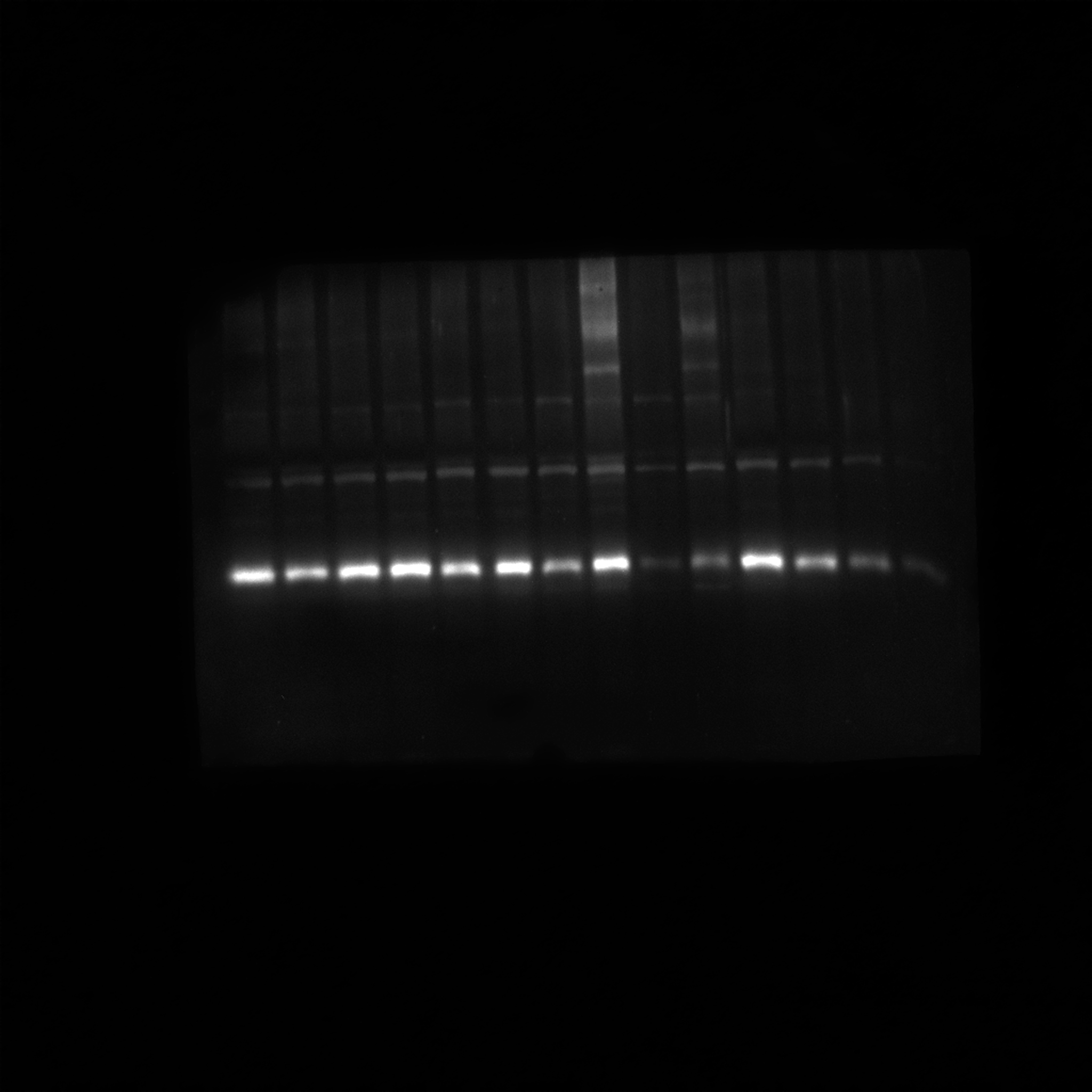

Supplement: Figure 3—source data 1. [file elife-75041-fig3-data1.zip › Fig 3 source data 1/petA.Tif]

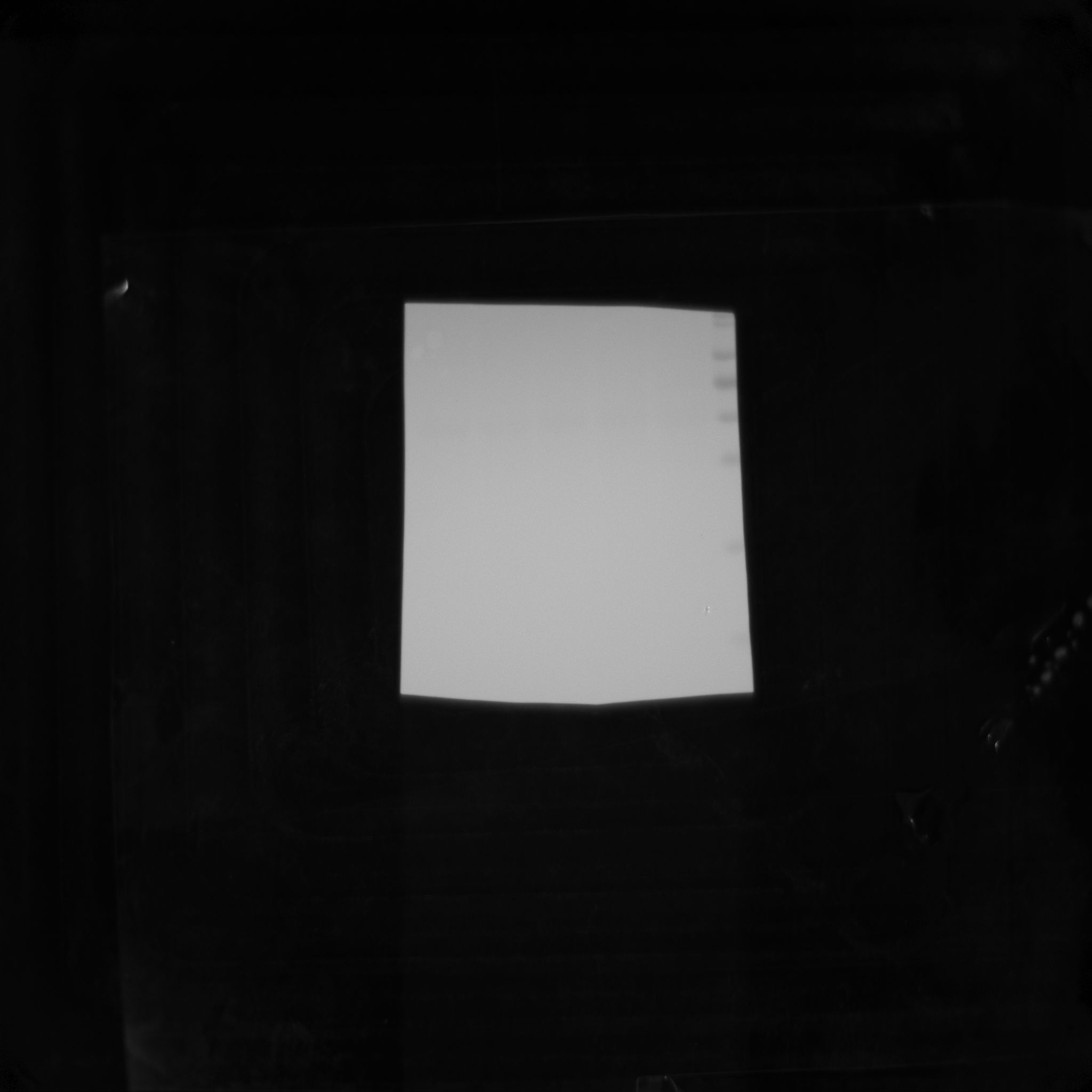

Supplement: Figure 3—source data 2. [file elife-75041-fig3-data2.zip › Fig 3 source data 2/lhcb1_with_N_epi_light.jpg]

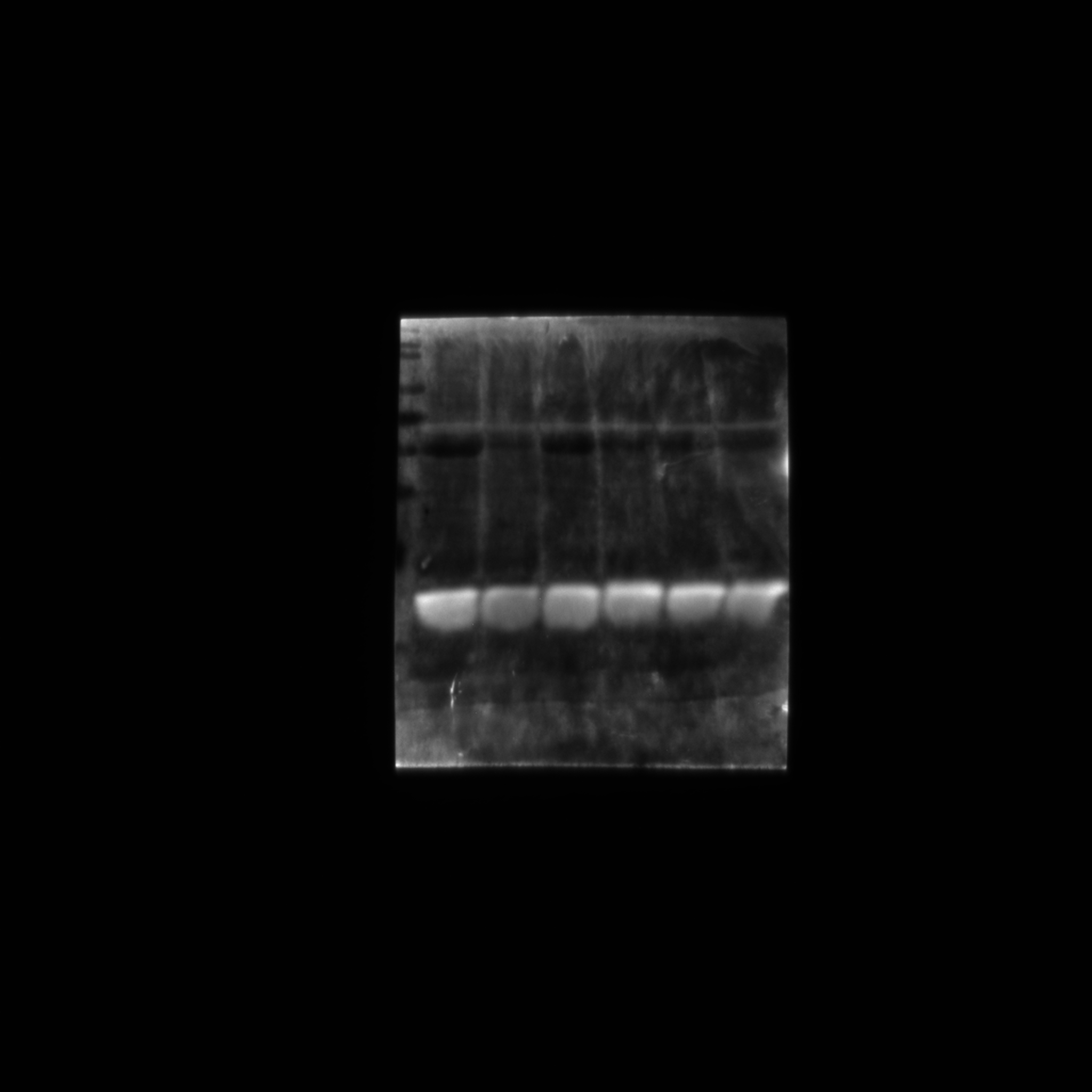

Supplement: Figure 3—source data 2. [file elife-75041-fig3-data2.zip › Fig 3 source data 2/lhca1_without_N.Tif]

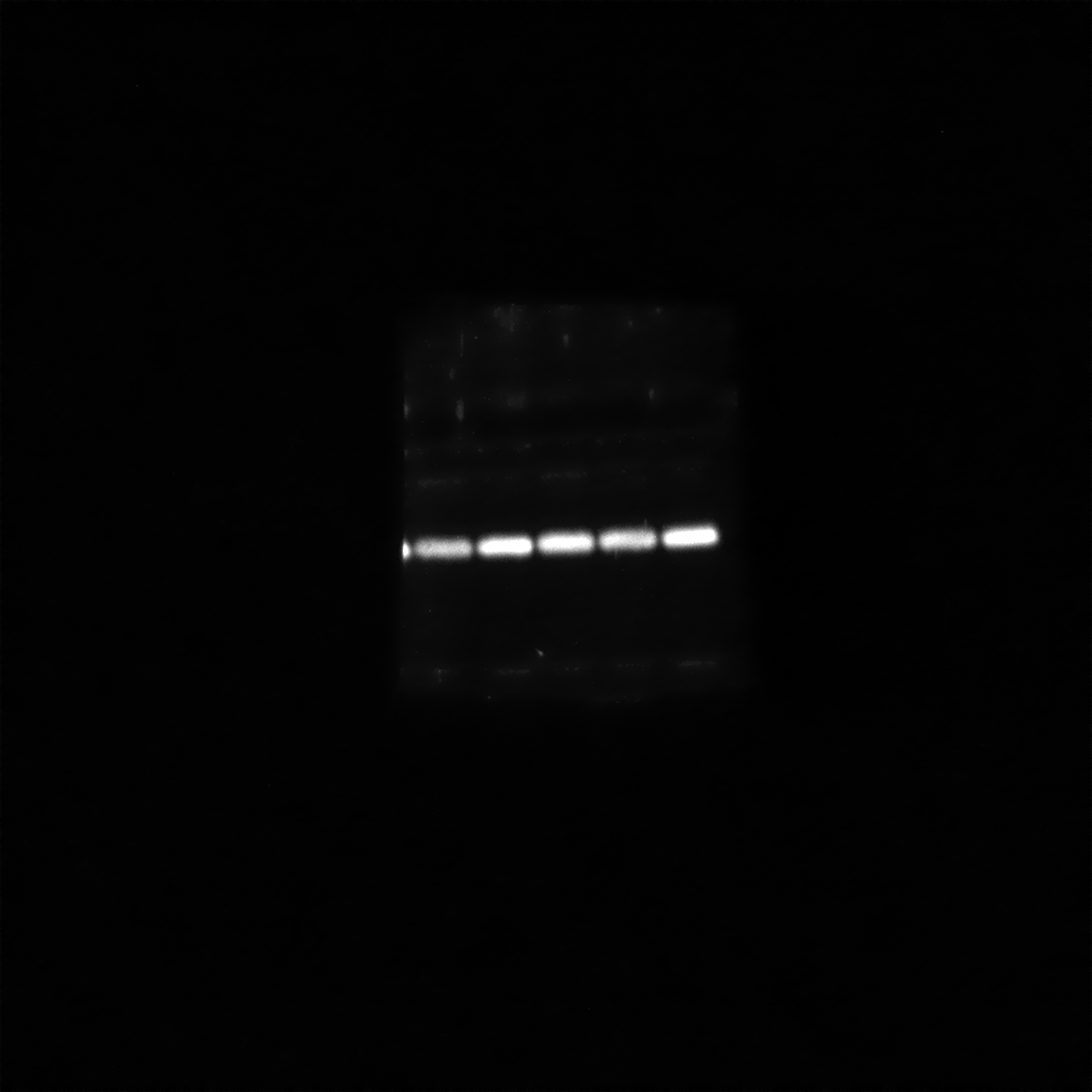

Supplement: Figure 3—source data 2. [file elife-75041-fig3-data2.zip › Fig 3 source data 2/lhcb1_with_N.Tif]

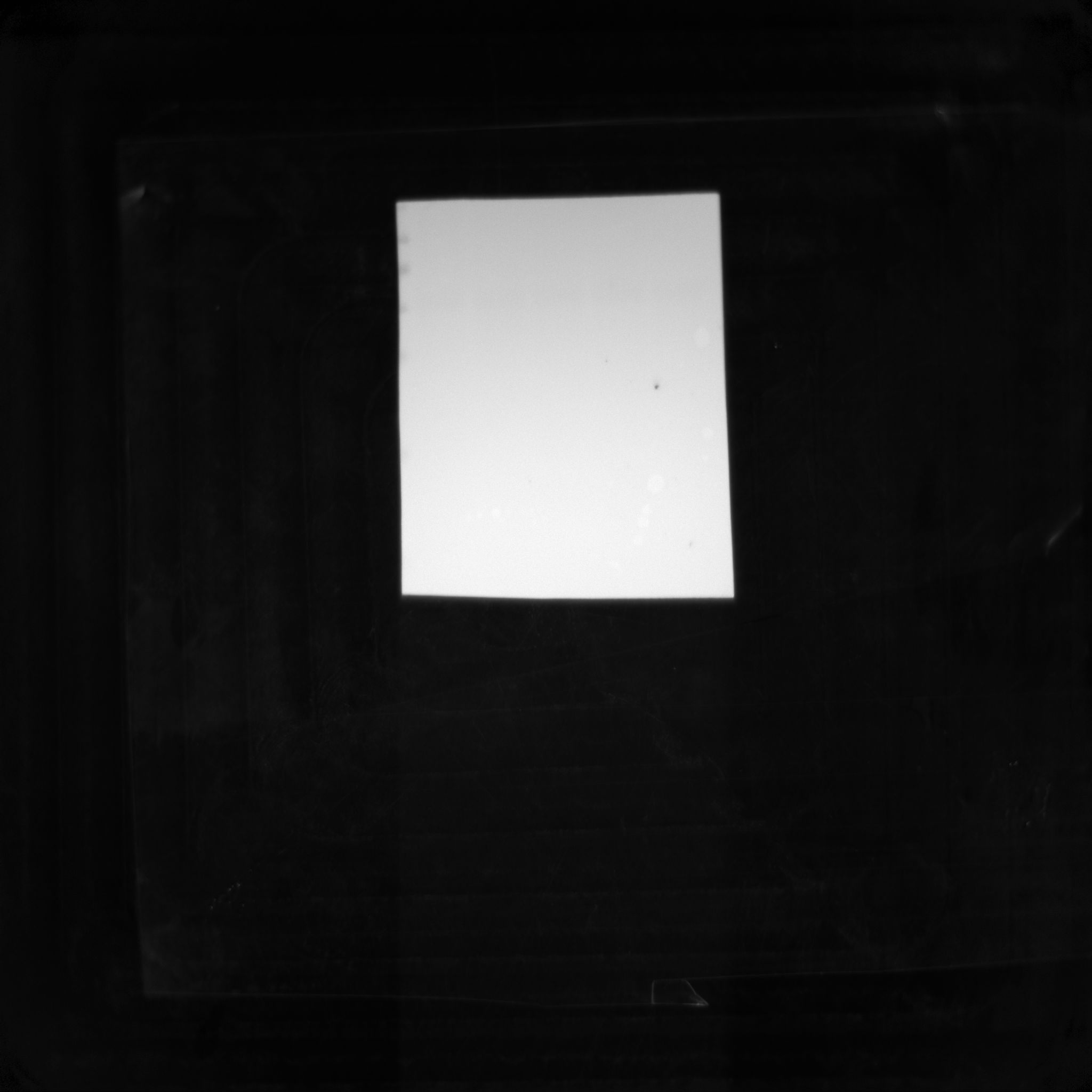

Supplement: Figure 3—source data 2. [file elife-75041-fig3-data2.zip › Fig 3 source data 2/PsaD_with_N_epi_light.jpg]

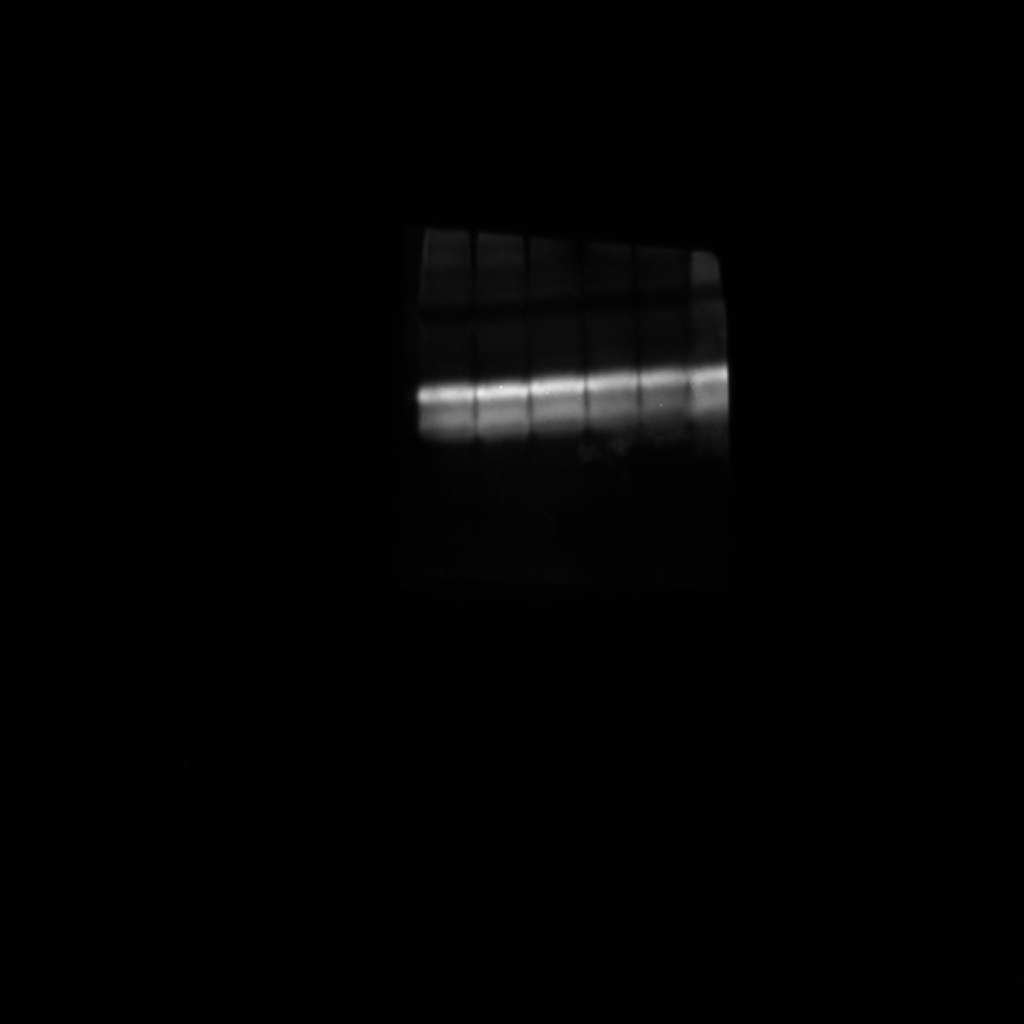

Supplement: Figure 3—source data 2. [file elife-75041-fig3-data2.zip › Fig 3 source data 2/PsbA_with_N.Tif]

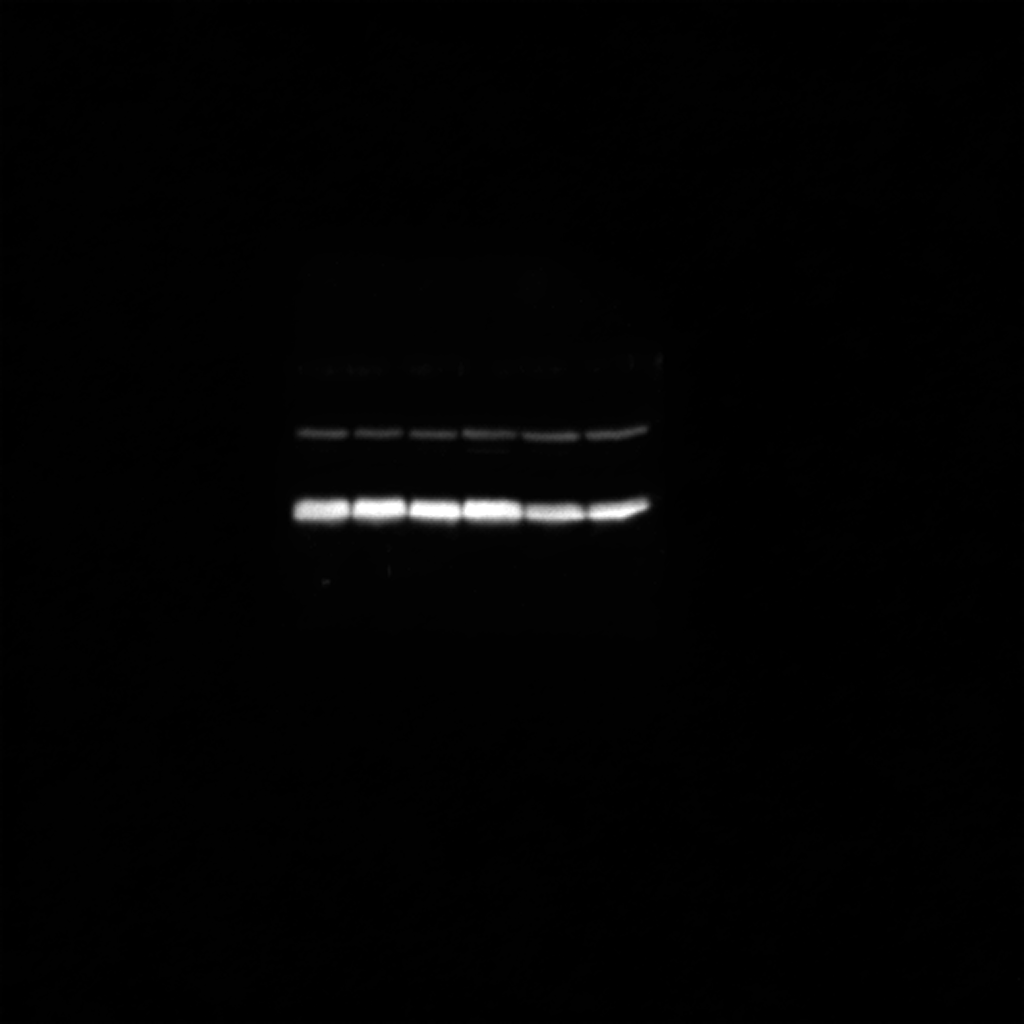

Supplement: Figure 3—source data 2. [file elife-75041-fig3-data2.zip › Fig 3 source data 2/lhcb1_without_N.Tif]

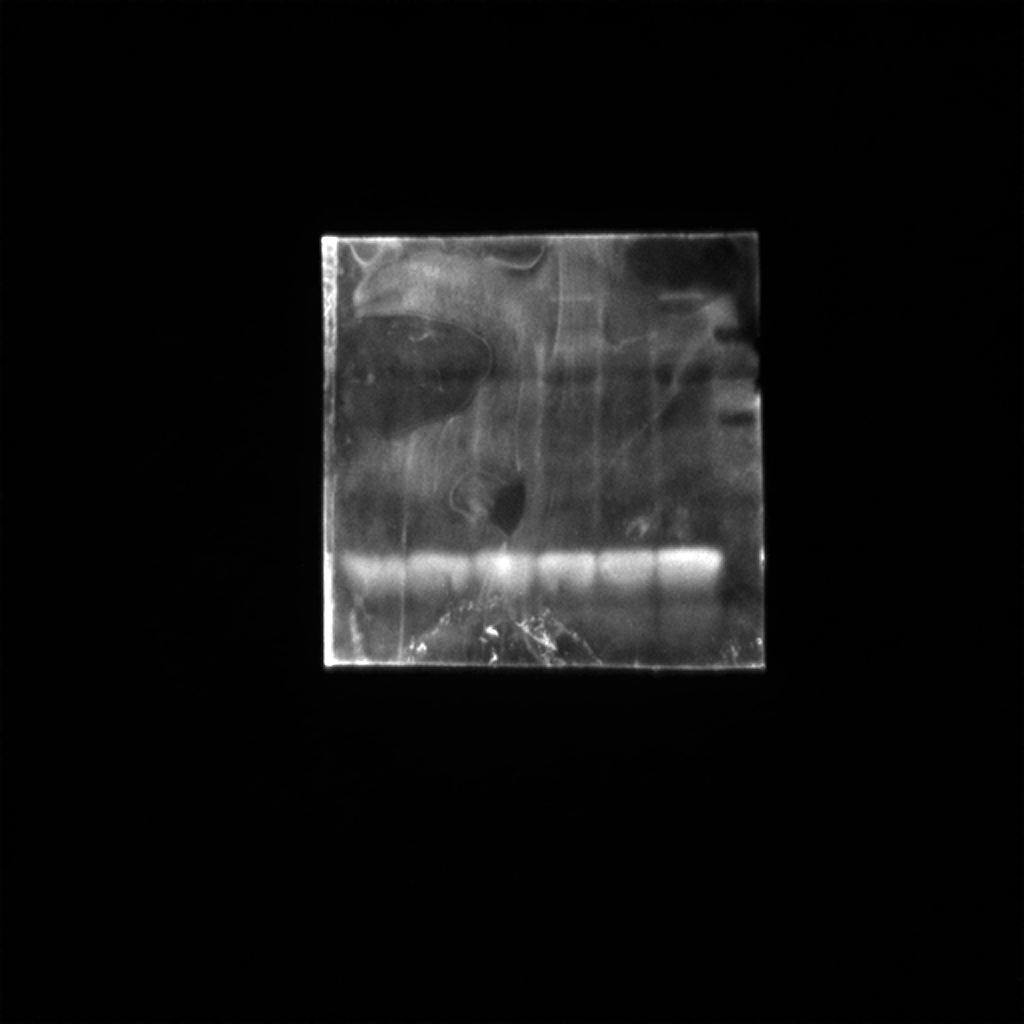

Supplement: Figure 3—source data 2. [file elife-75041-fig3-data2.zip › Fig 3 source data 2/psaD_without_N.Tif]

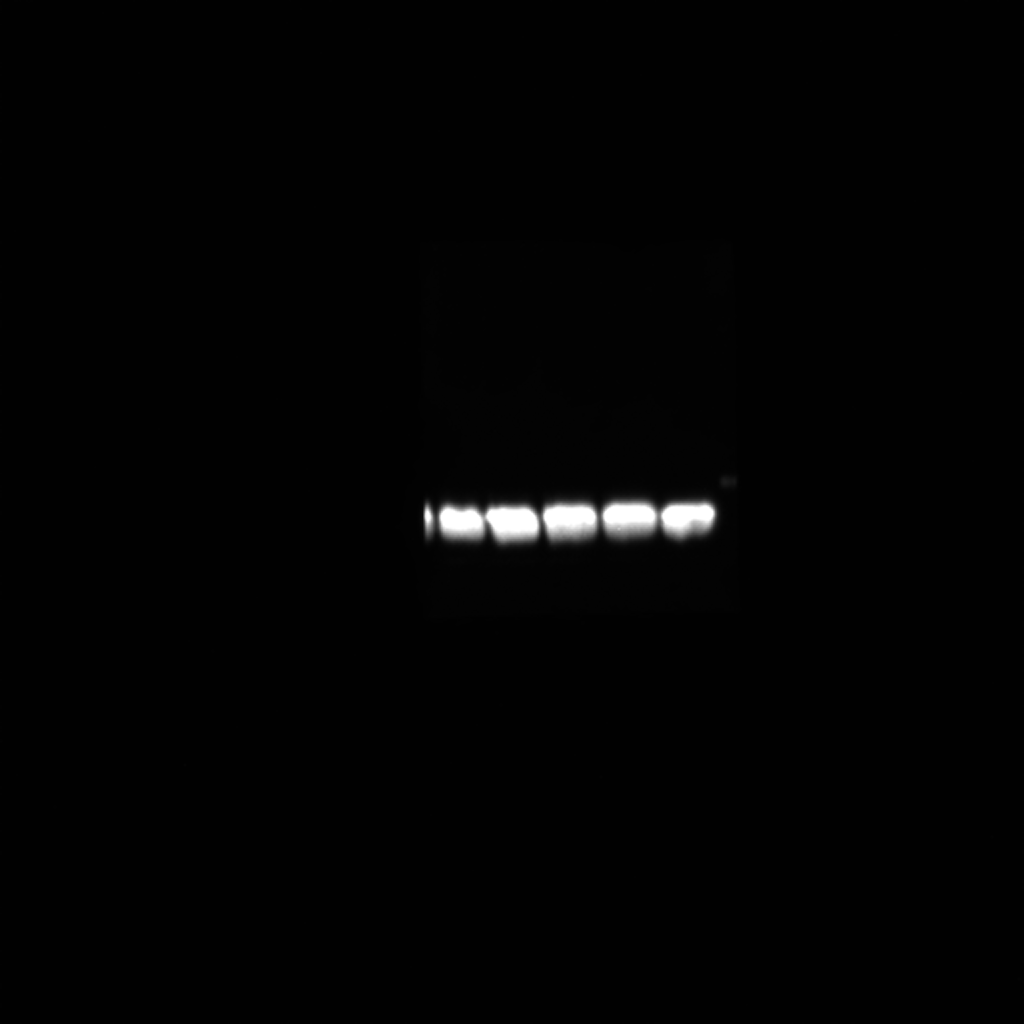

Supplement: Figure 3—source data 2. [file elife-75041-fig3-data2.zip › Fig 3 source data 2/lhca1_with_N.Tif]

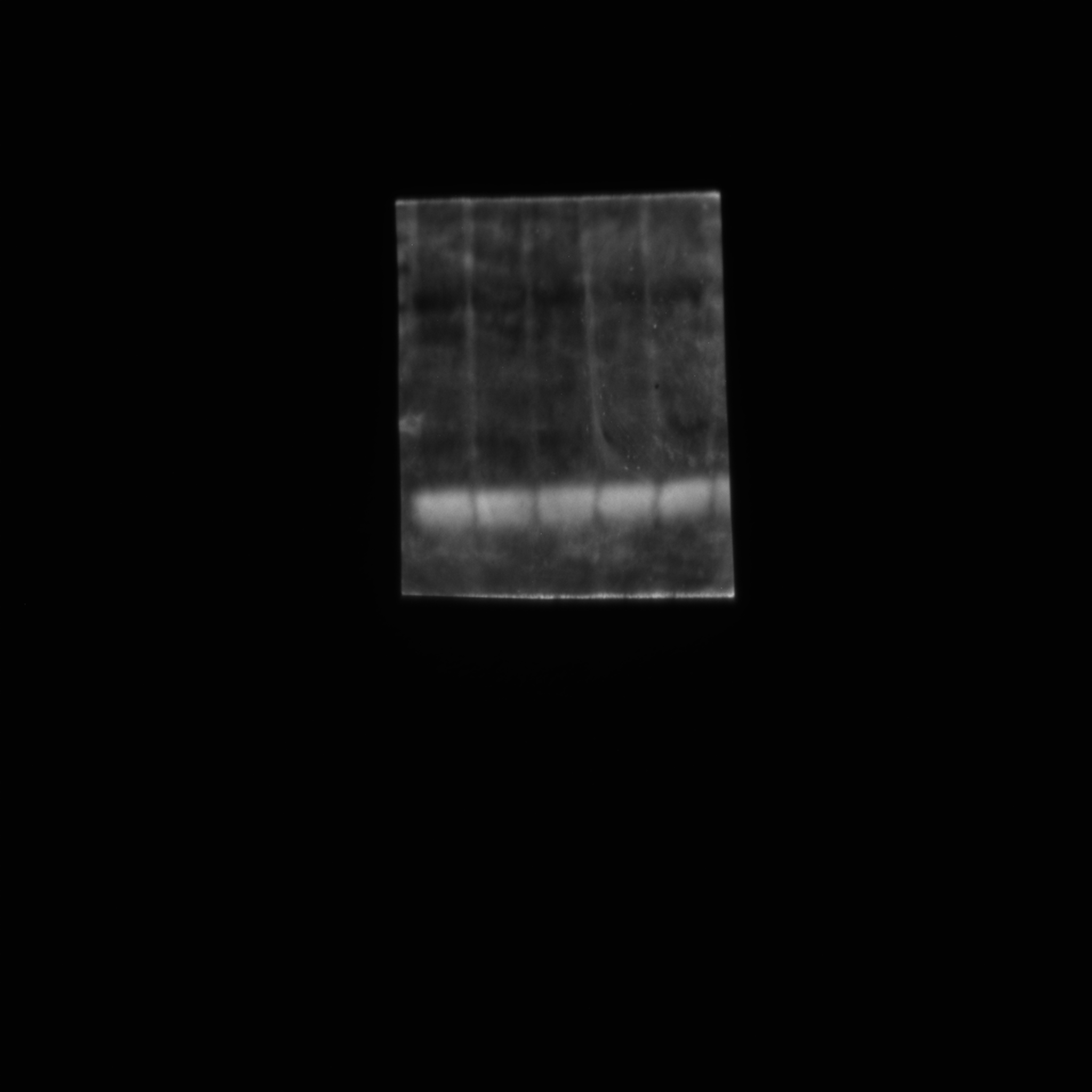

Supplement: Figure 3—source data 2. [file elife-75041-fig3-data2.zip › Fig 3 source data 2/PsaD_with_N.Tif]

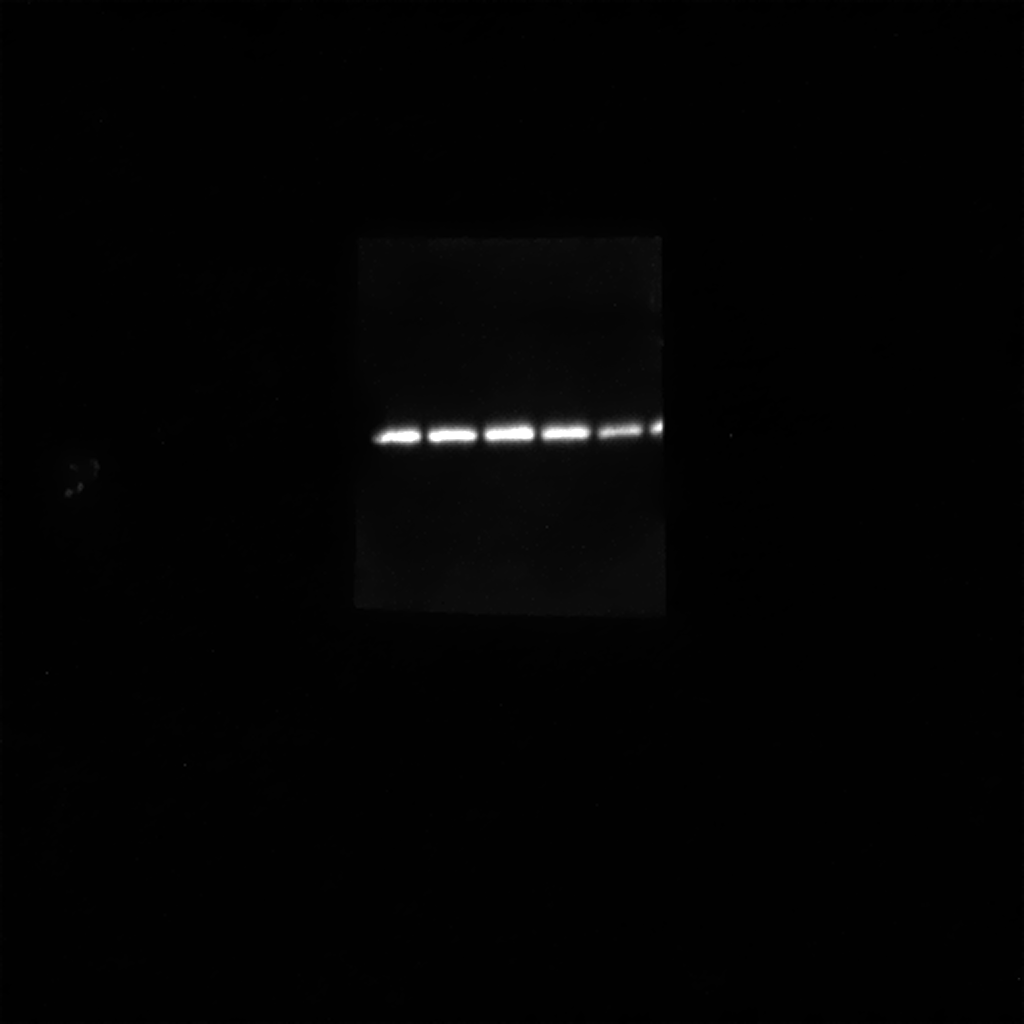

Supplement: Figure 3—source data 2. [file elife-75041-fig3-data2.zip › Fig 3 source data 2/petA_with_N.Tif]

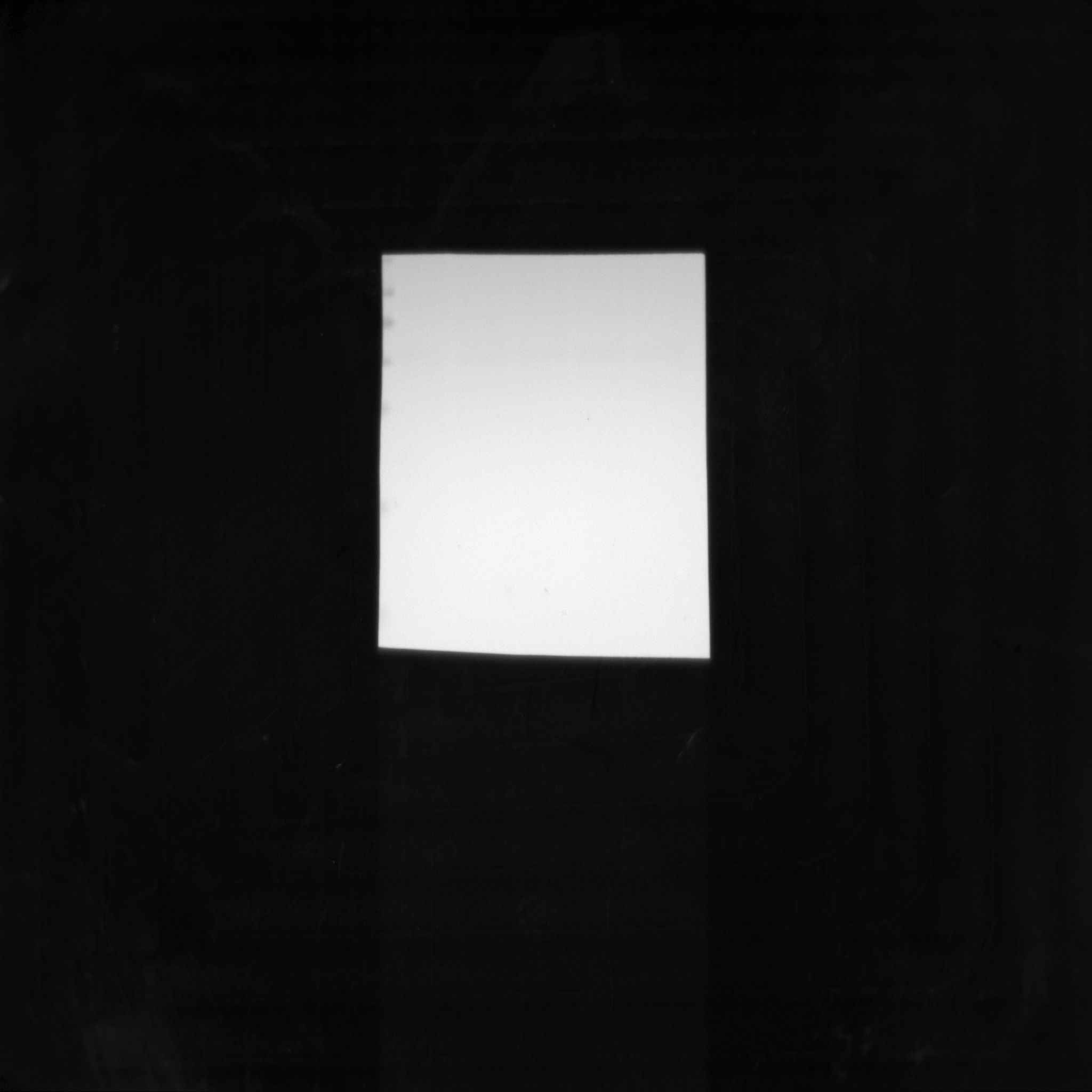

Supplement: Figure 3—source data 2. [file elife-75041-fig3-data2.zip › Fig 3 source data 2/petA_with_N_epi_light.jpg]

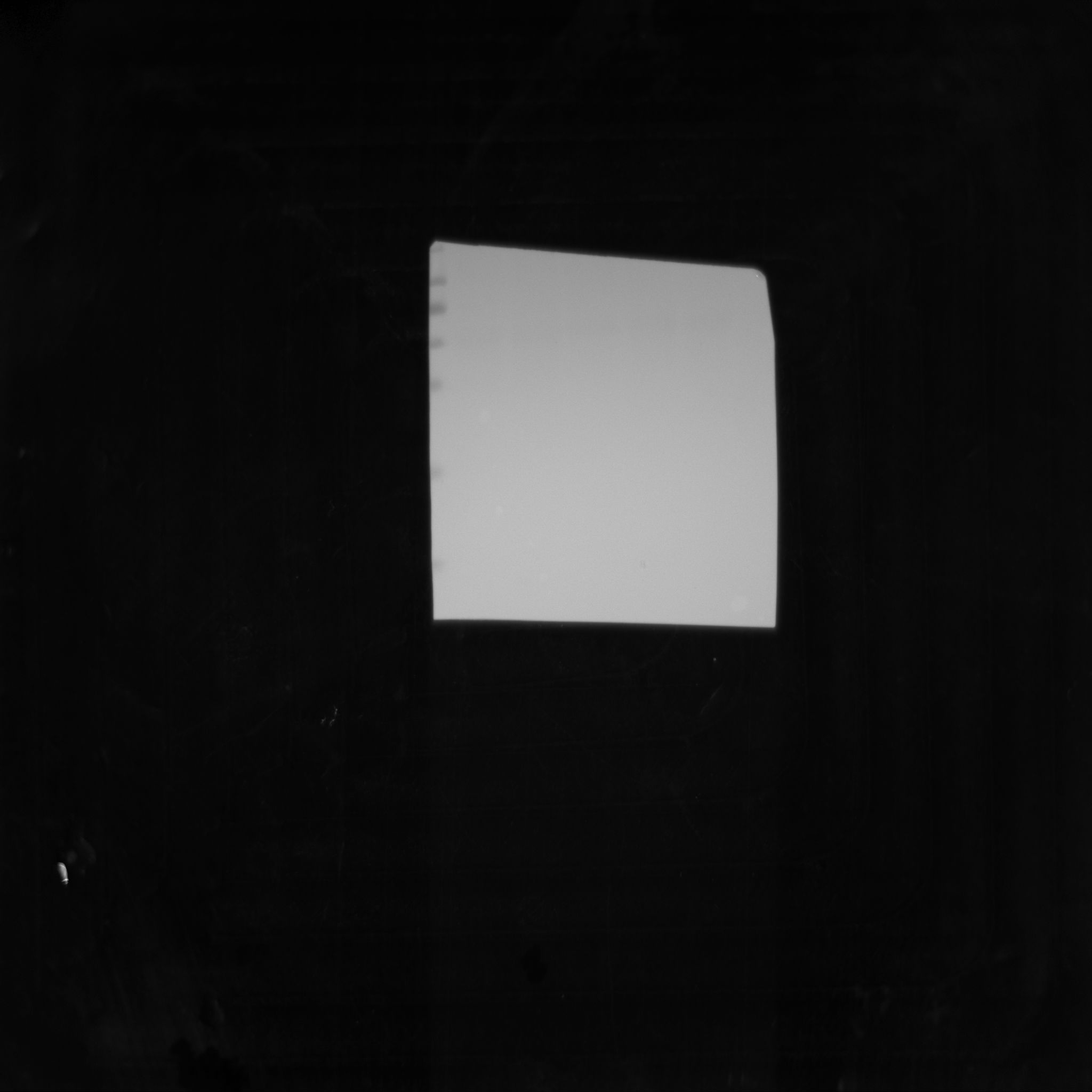

Supplement: Figure 3—source data 2. [file elife-75041-fig3-data2.zip › Fig 3 source data 2/PsbA_with_N_epi_light.jpg]

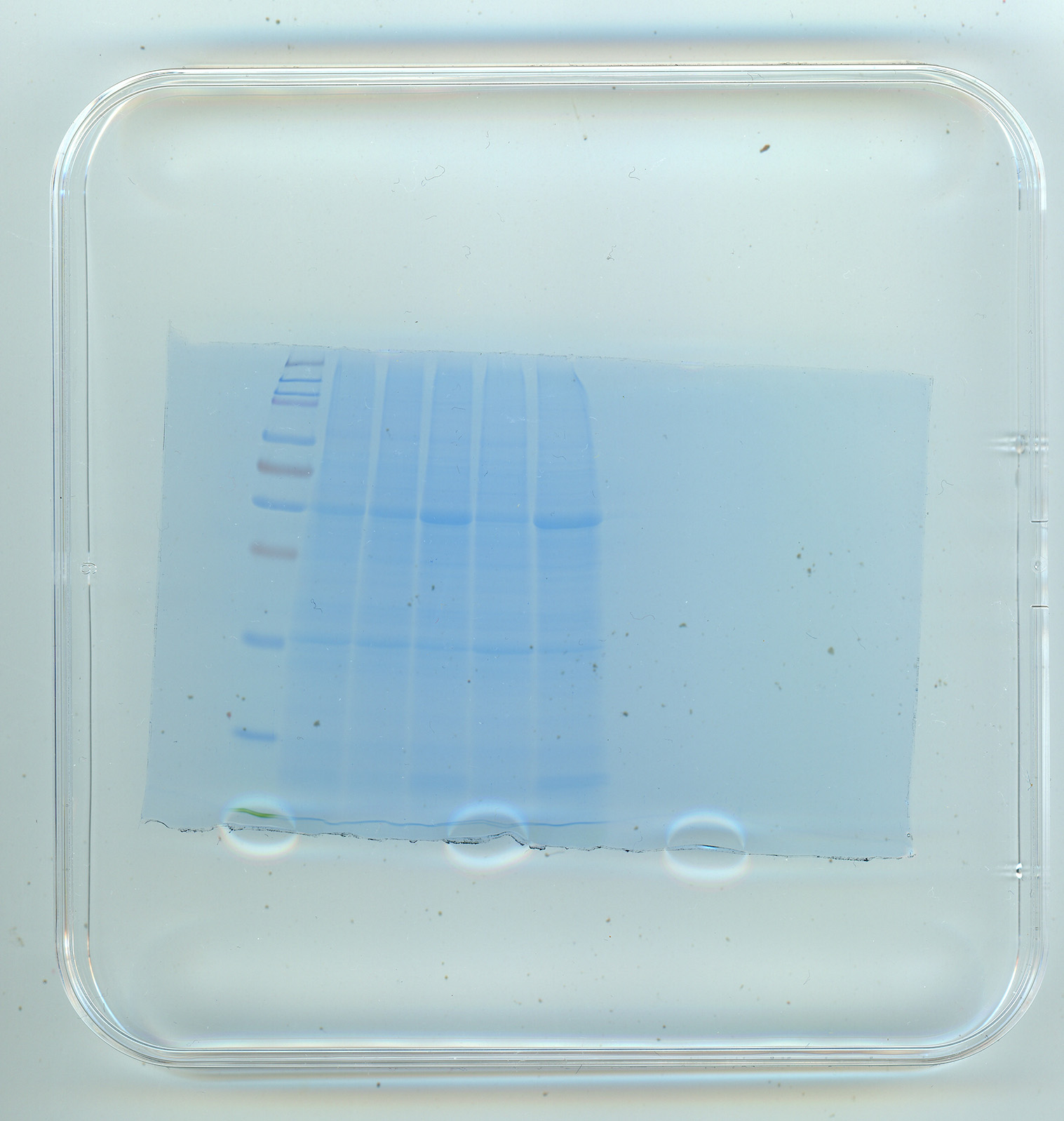

Supplement: Figure 3—source data 2. [file elife-75041-fig3-data2.zip › Fig 3 source data 2/coomassie_blue_without_N.jpg]

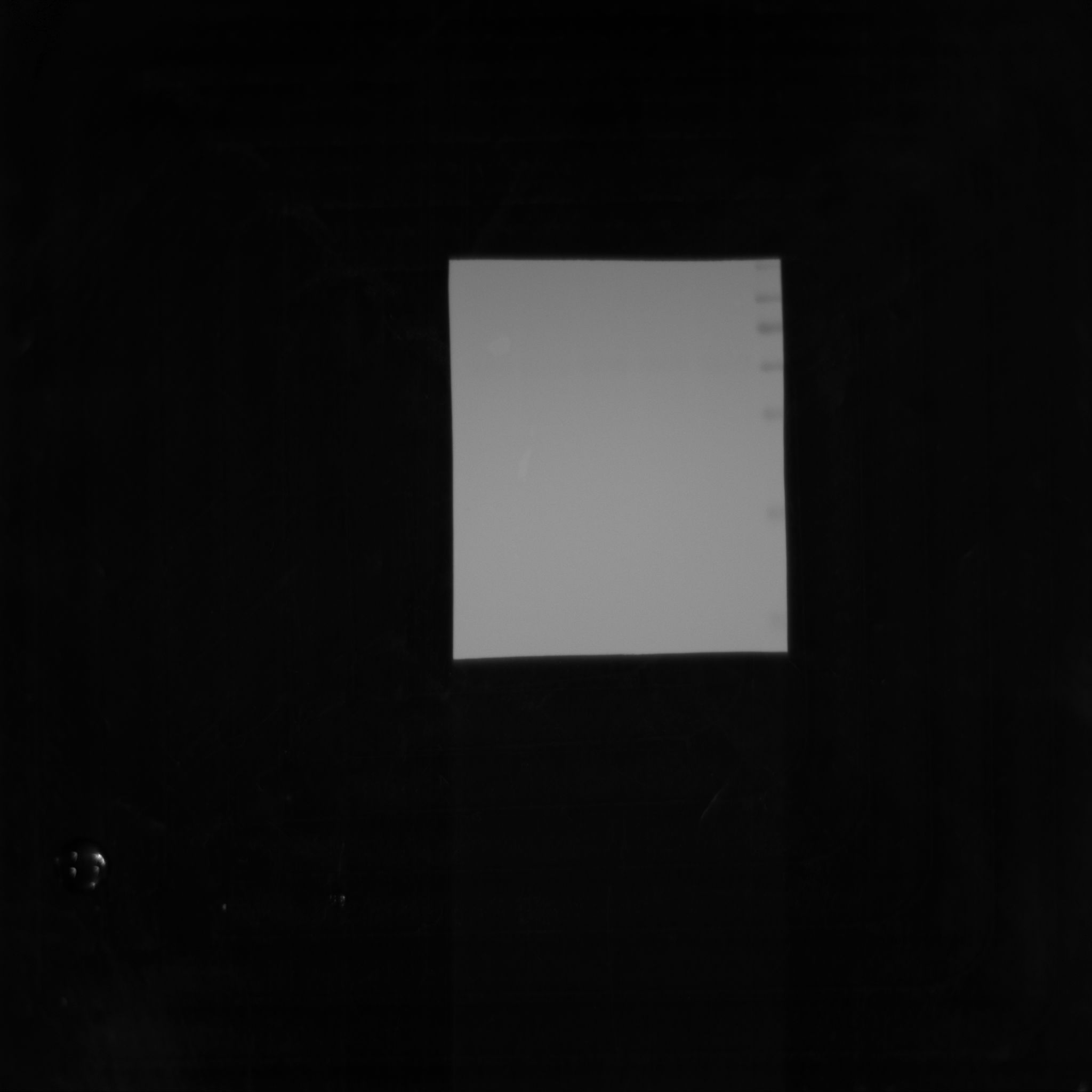

Supplement: Figure 3—source data 2. [file elife-75041-fig3-data2.zip › Fig 3 source data 2/lhca1_with_N_epi_light.jpg]

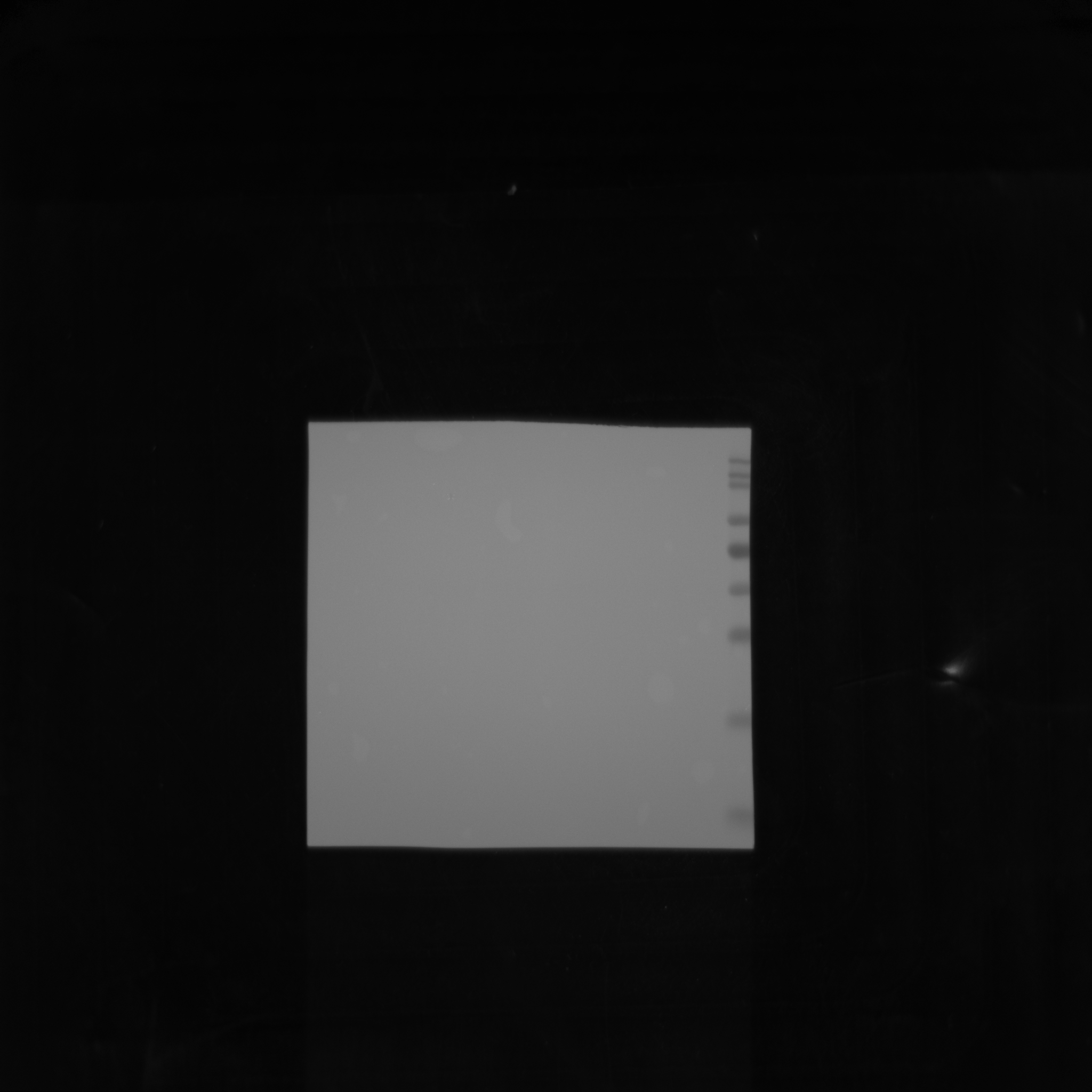

Supplement: Figure 3—source data 2. [file elife-75041-fig3-data2.zip › Fig 3 source data 2/PsbA_without_N_epi_light.Tif]

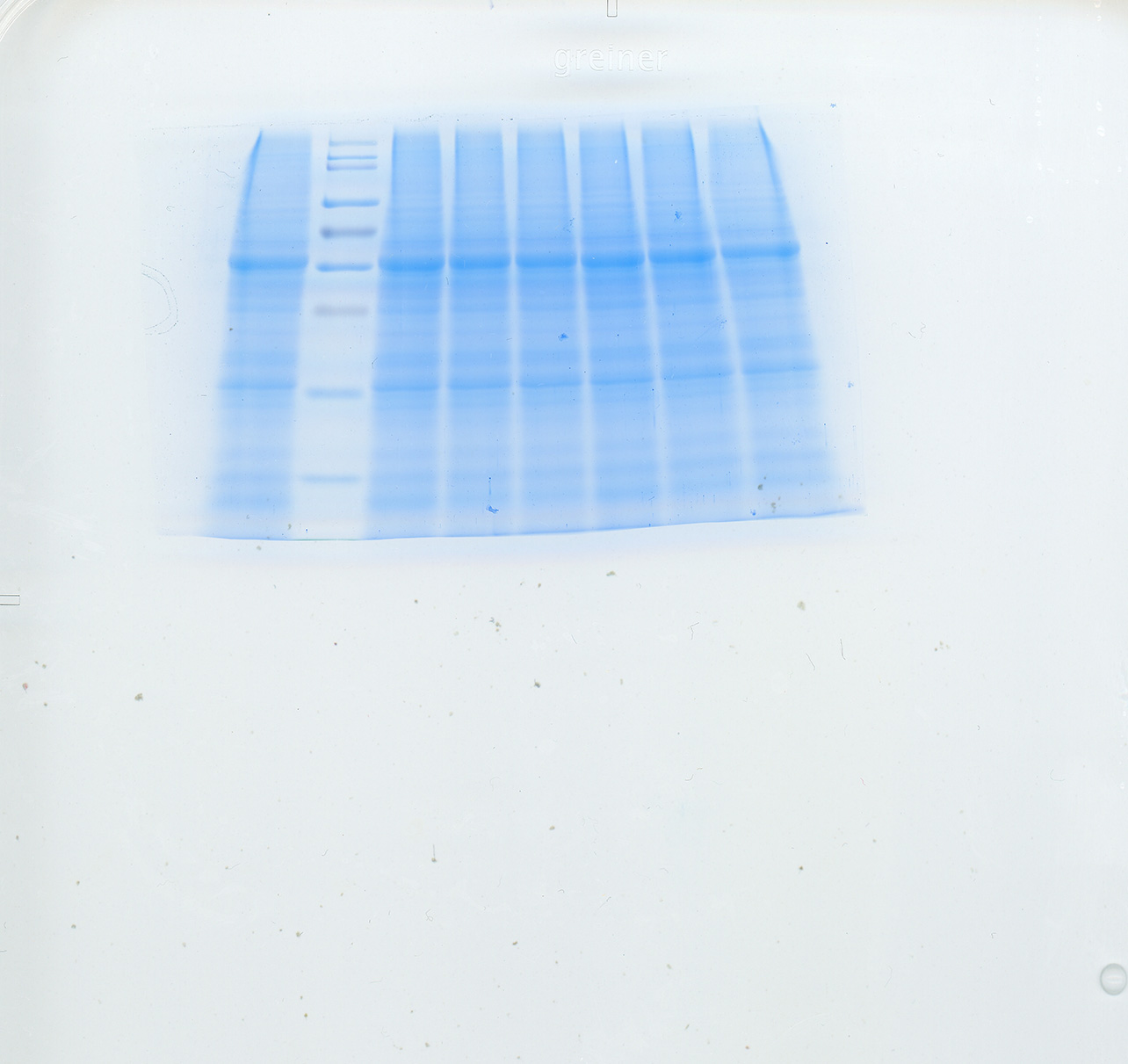

Supplement: Figure 3—source data 2. [file elife-75041-fig3-data2.zip › Fig 3 source data 2/coomssie_blue_with_N.jpg]

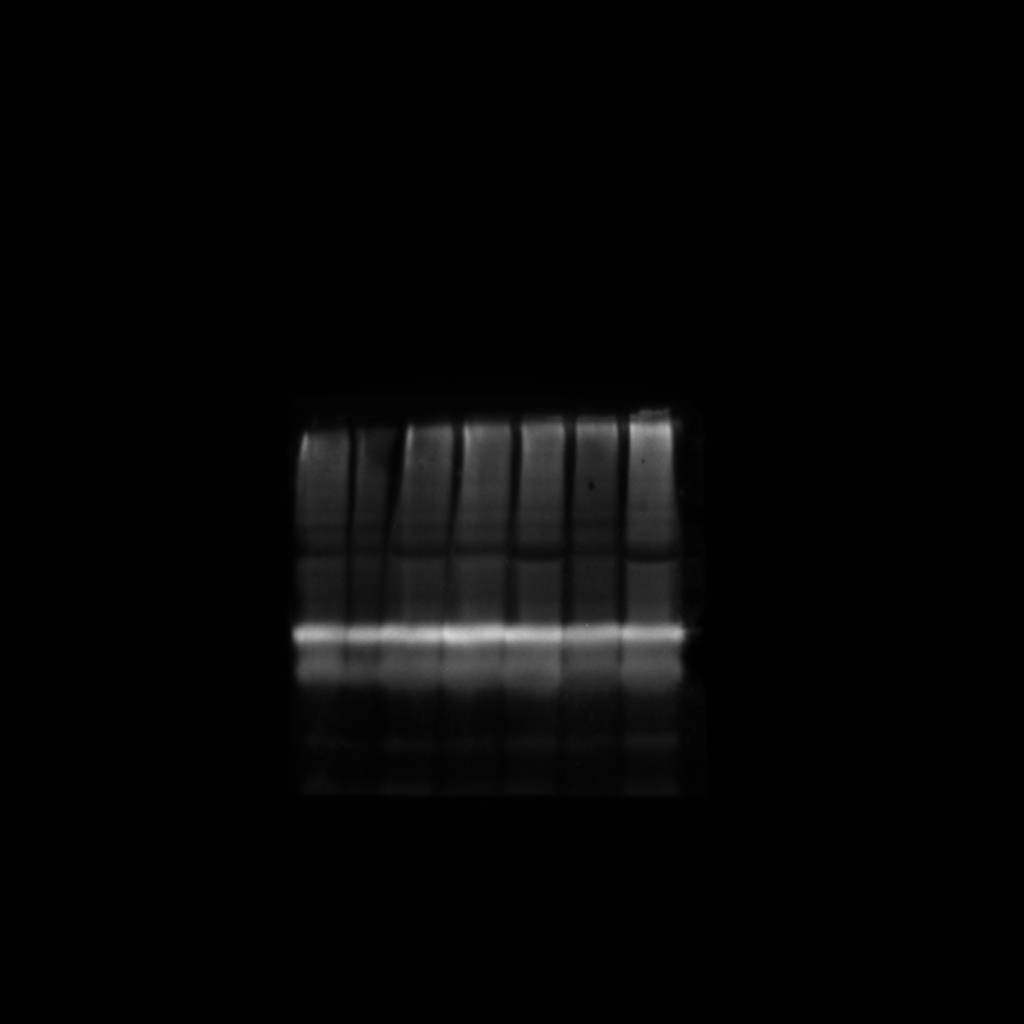

Supplement: Figure 3—source data 2. [file elife-75041-fig3-data2.zip › Fig 3 source data 2/PsbA_without_N.Tif]

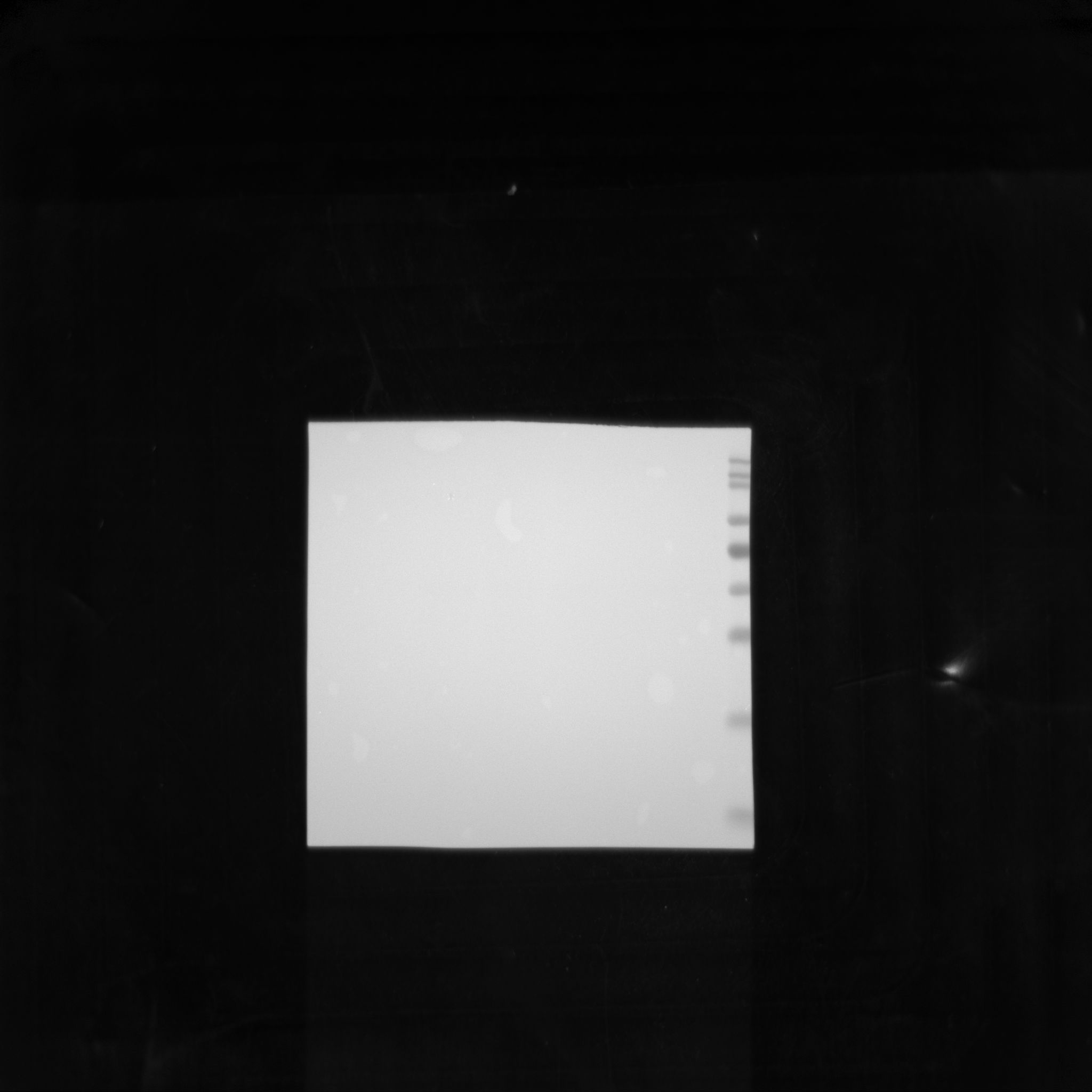

Supplement: Figure 3—source data 2. [file elife-75041-fig3-data2.zip › Fig 3 source data 2/PsbA_without_N_epi_light.jpg]

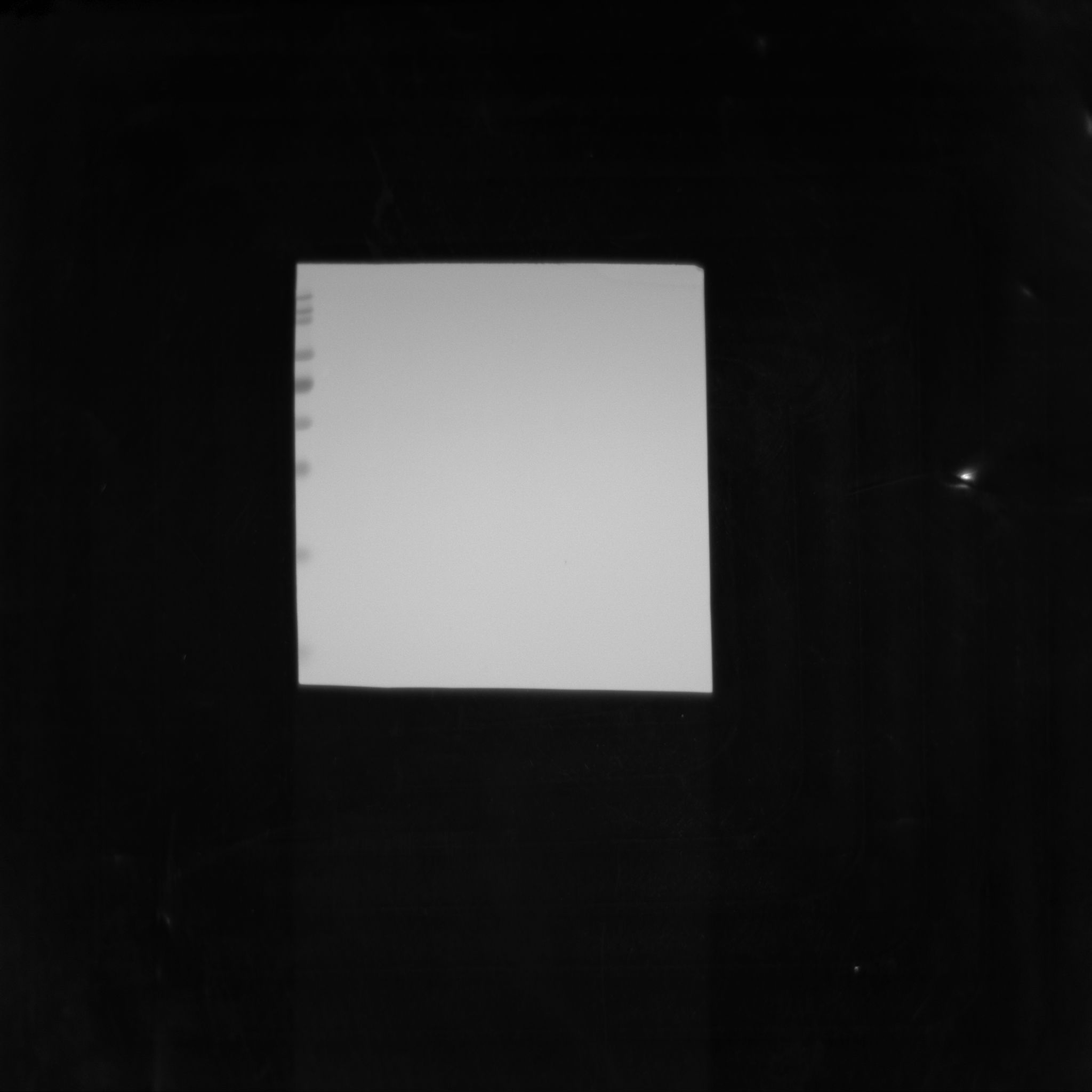

Supplement: Figure 3—source data 2. [file elife-75041-fig3-data2.zip › Fig 3 source data 2/lhcb1_without_N_epi_light.jpg]

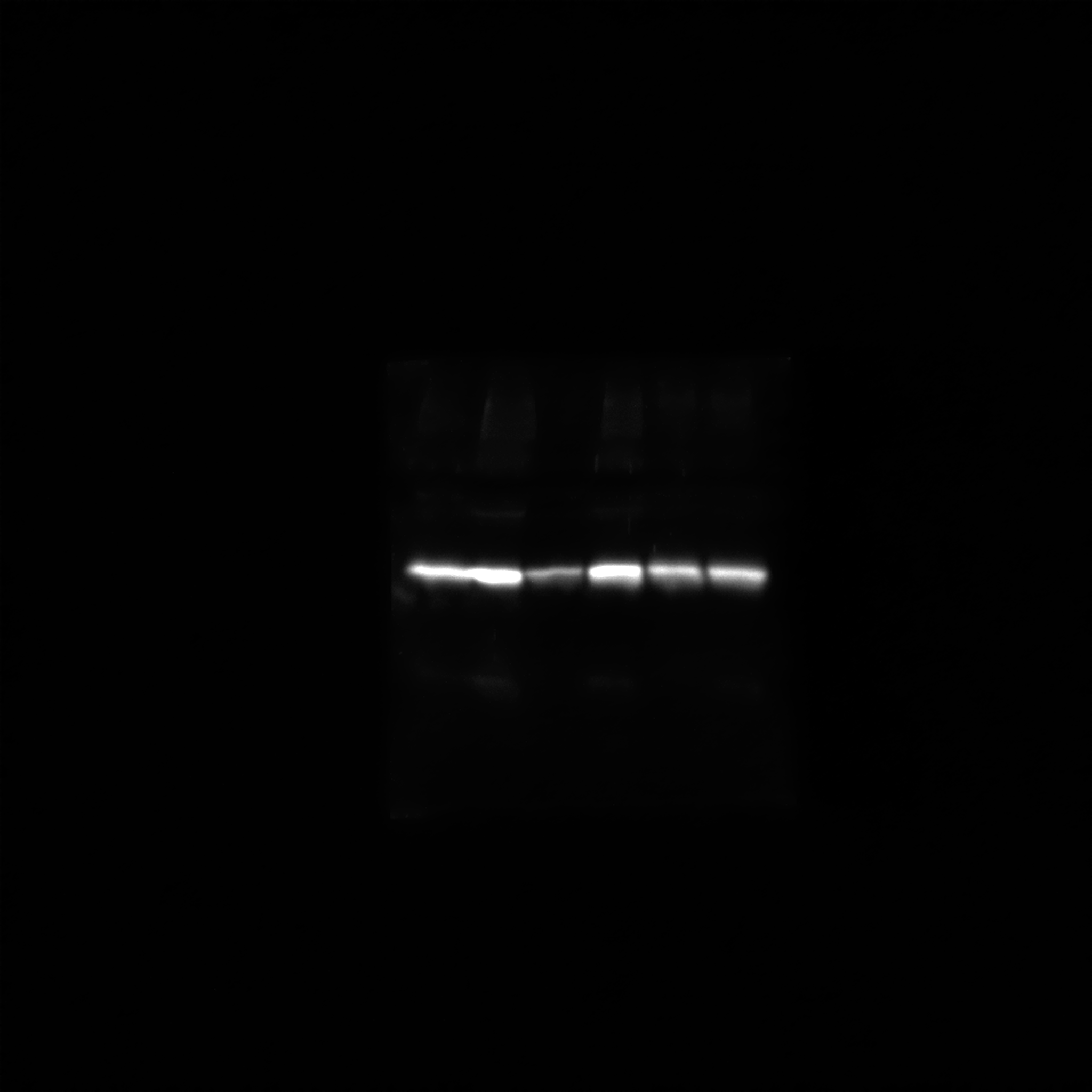

Supplement: Figure 3—source data 2. [file elife-75041-fig3-data2.zip › Fig 3 source data 2/PetA_without_N.Tif]

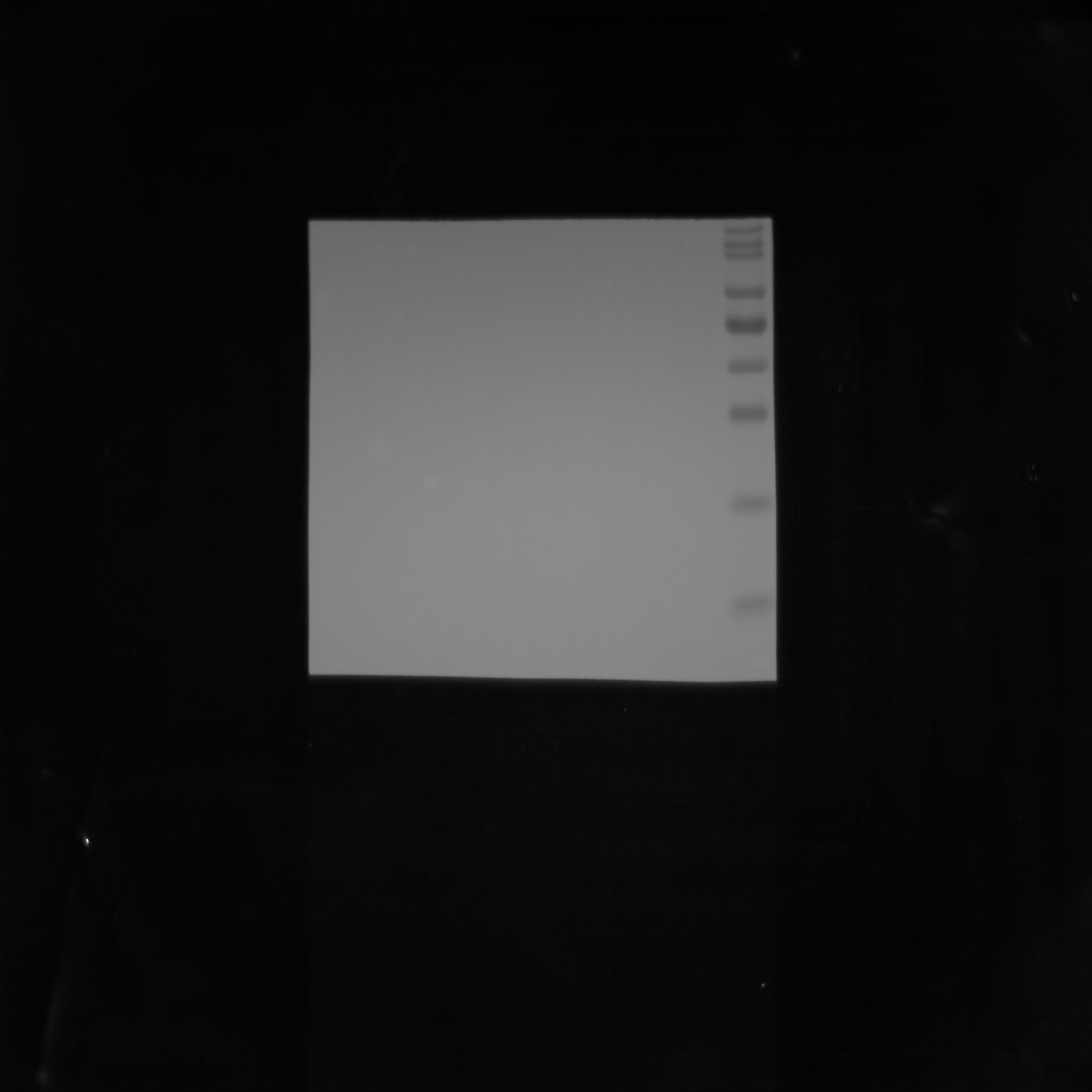

Supplement: Figure 3—source data 2. [file elife-75041-fig3-data2.zip › Fig 3 source data 2/psaD_without_N_epi_light.jpg]

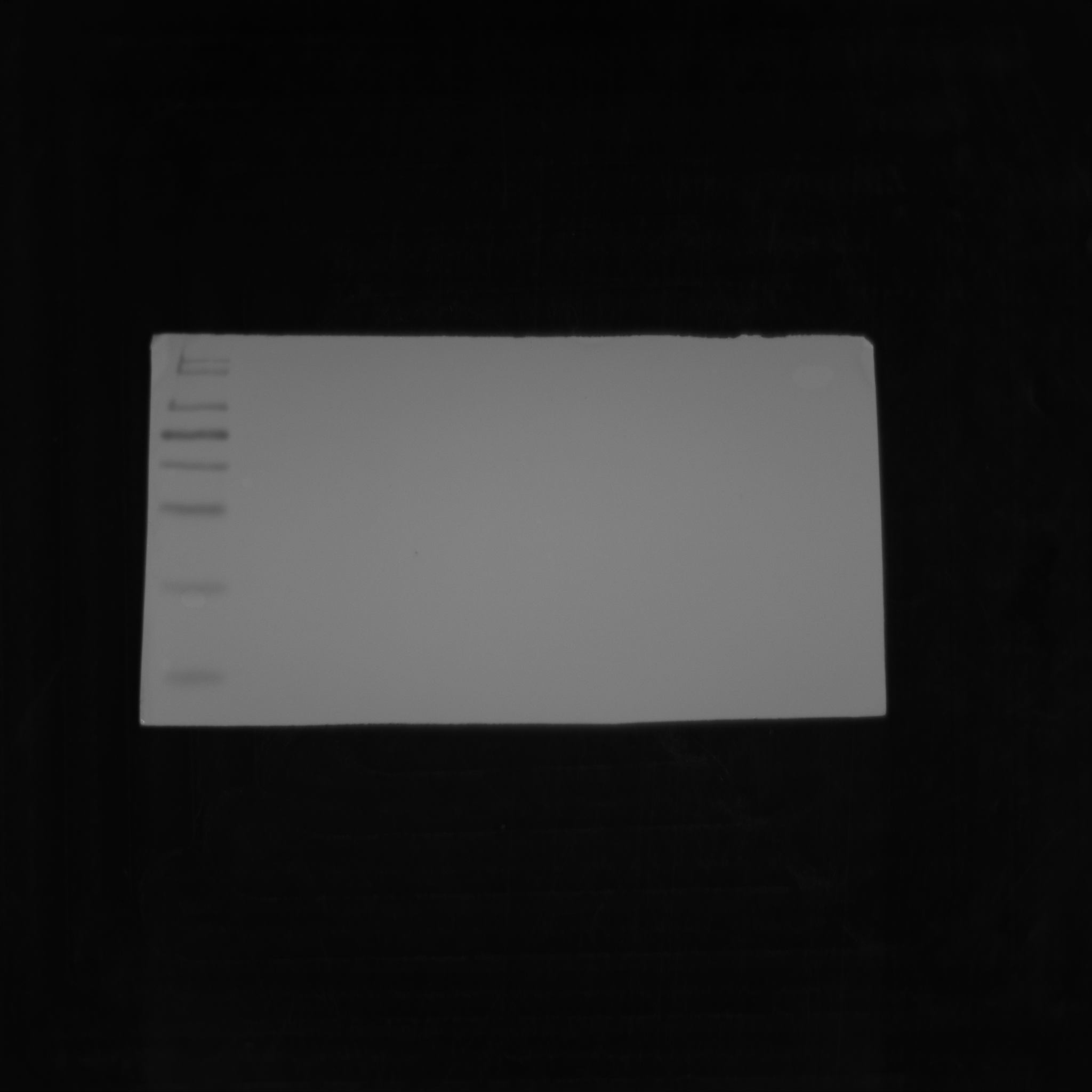

Supplement: Figure 3—source data 3. [file elife-75041-fig3-data3.zip › Fig 3 source data 3/Fig_3_supp1_source_data/petA_epi_light.Tif]

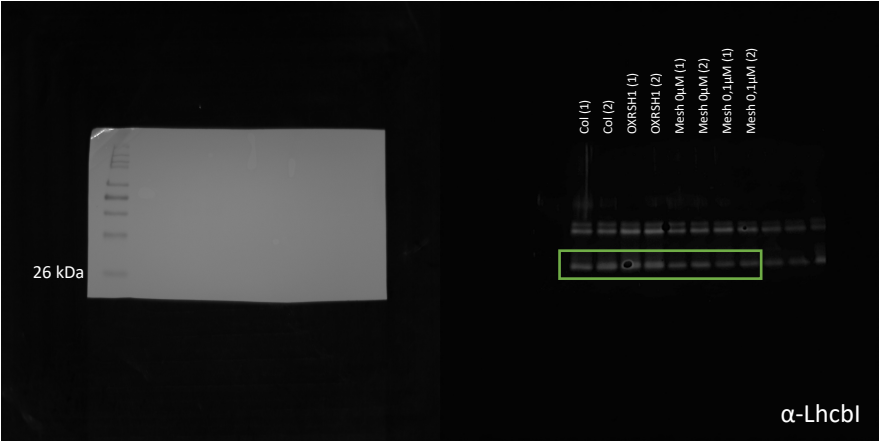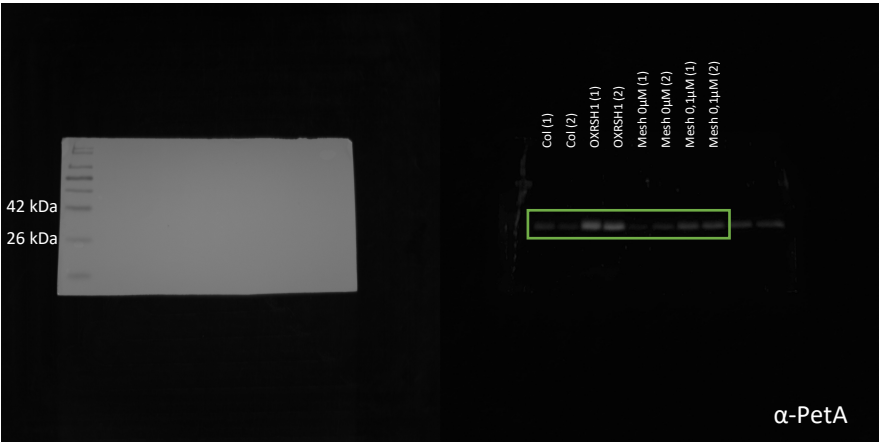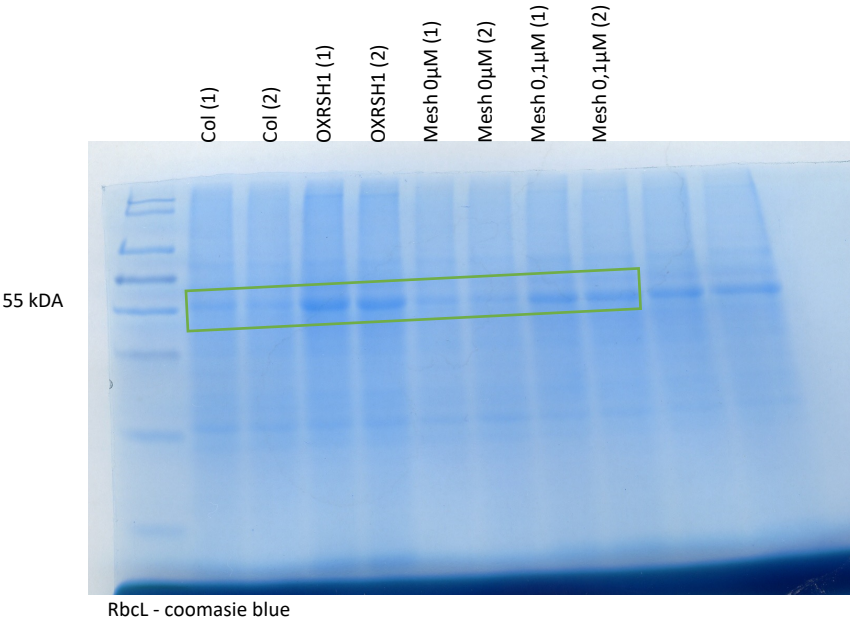

Fig. S5 source data : Full sized immune blots and coomasie blue.

Supplement: Figure 3—source data 3. [file elife-75041-fig3-data3.zip › Fig 3 source data 3/Fig_3_supp1_source_data/Fig3_supp1_source_data_summary.pdf]

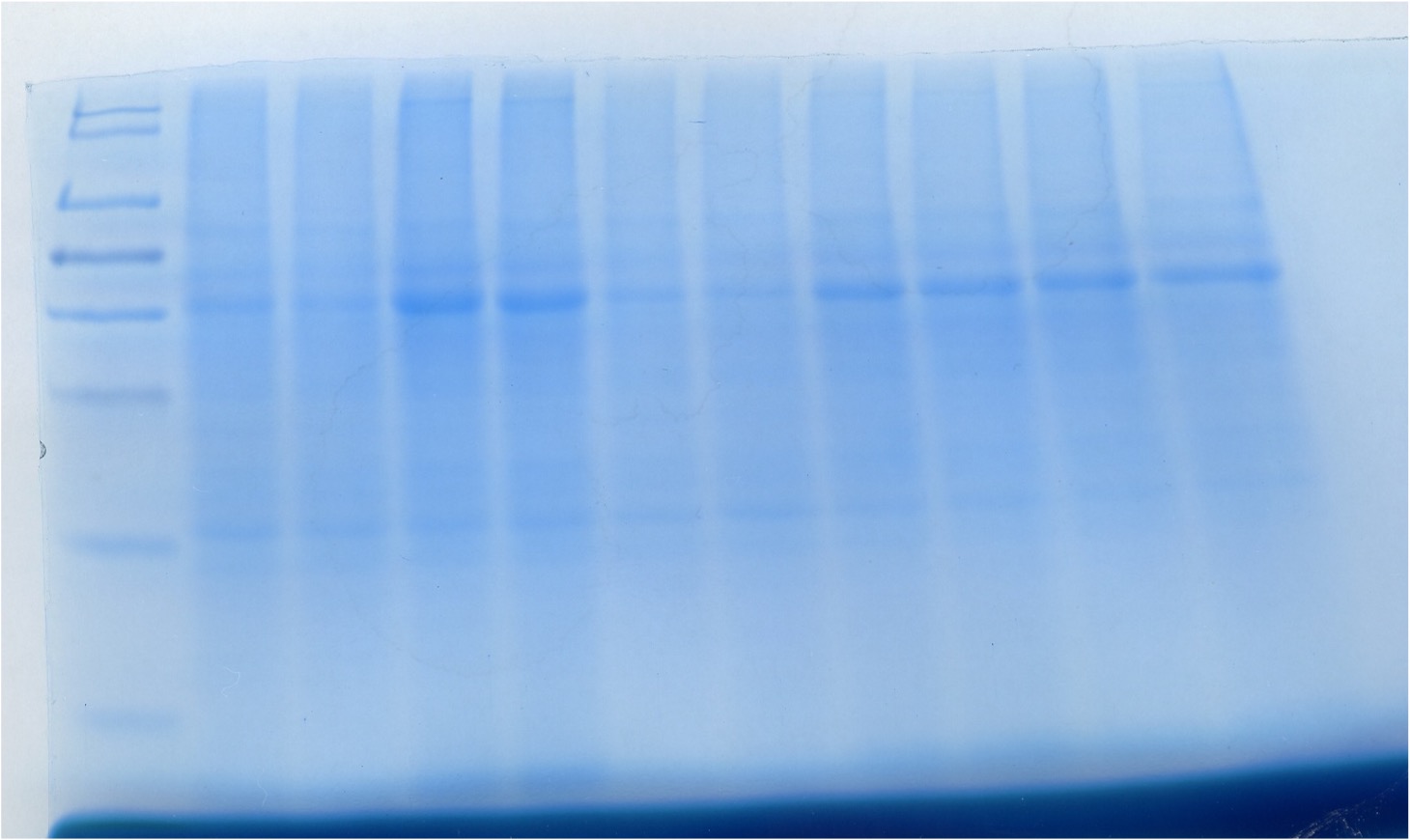

Supplement: Figure 3—source data 3. [file elife-75041-fig3-data3.zip › Fig 3 source data 3/Fig_3_supp1_source_data/coomasie_blue_MESH.jpg]

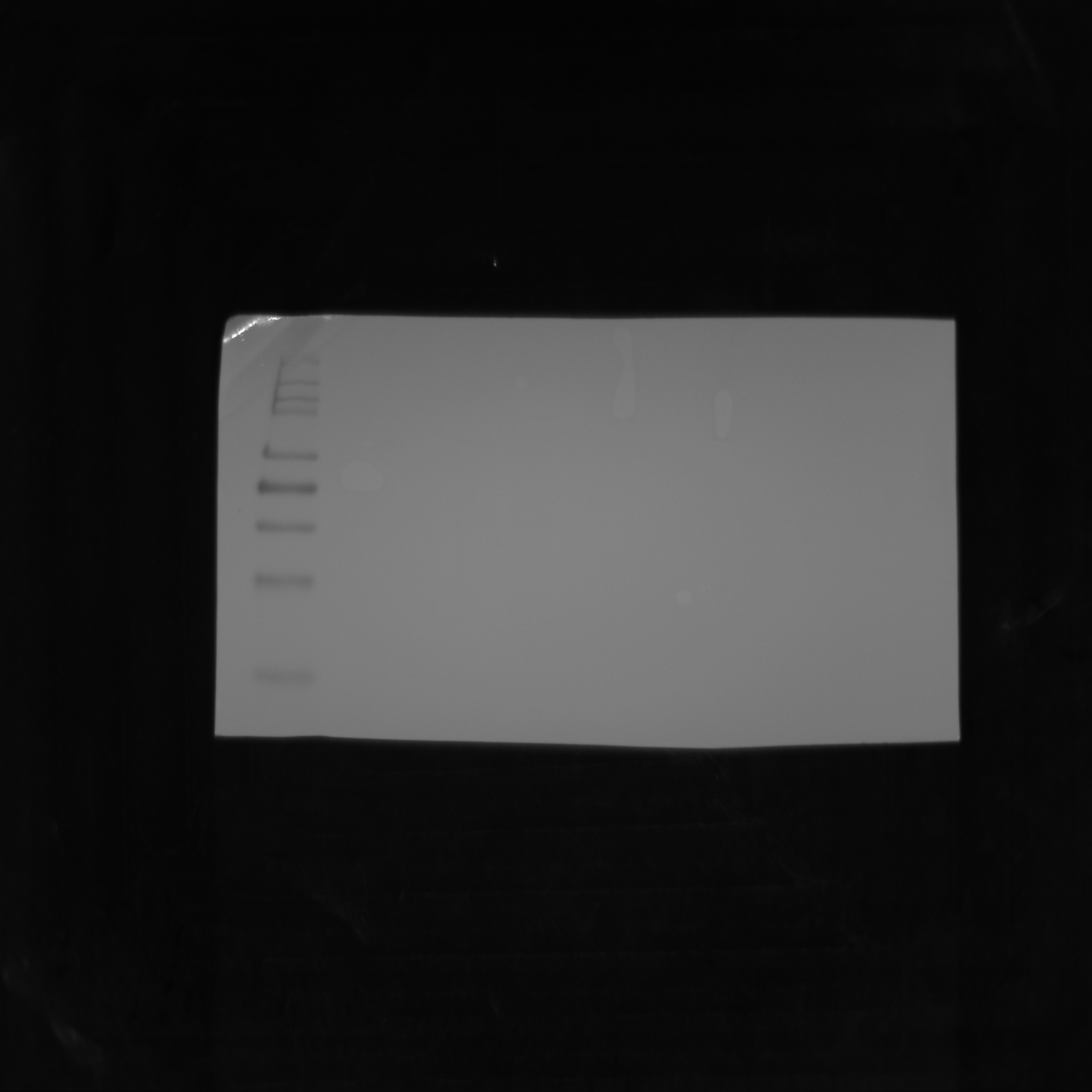

Supplement: Figure 3—source data 3. [file elife-75041-fig3-data3.zip › Fig 3 source data 3/Fig_3_supp1_source_data/LhcbI_epi_light.Tif]

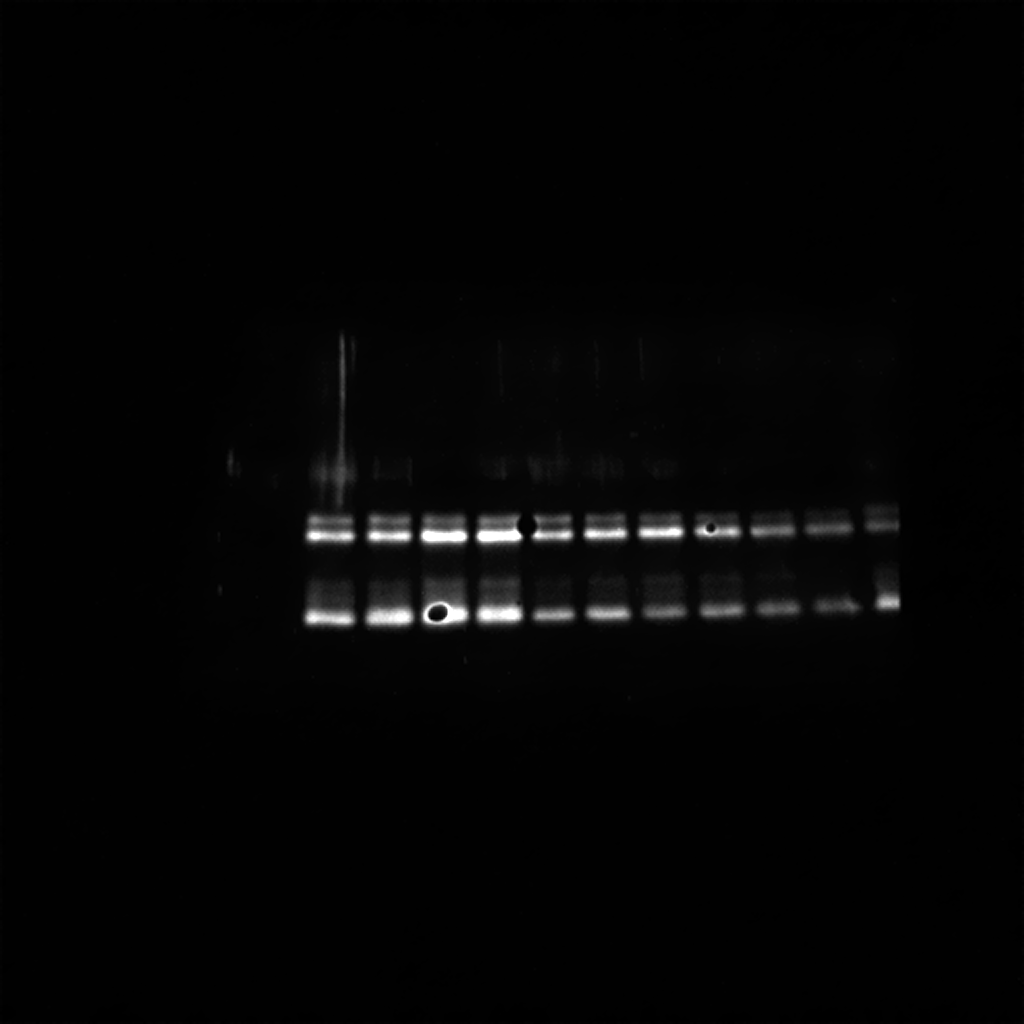

Supplement: Figure 3—source data 3. [file elife-75041-fig3-data3.zip › Fig 3 source data 3/Fig_3_supp1_source_data/LhcbI.Tif]

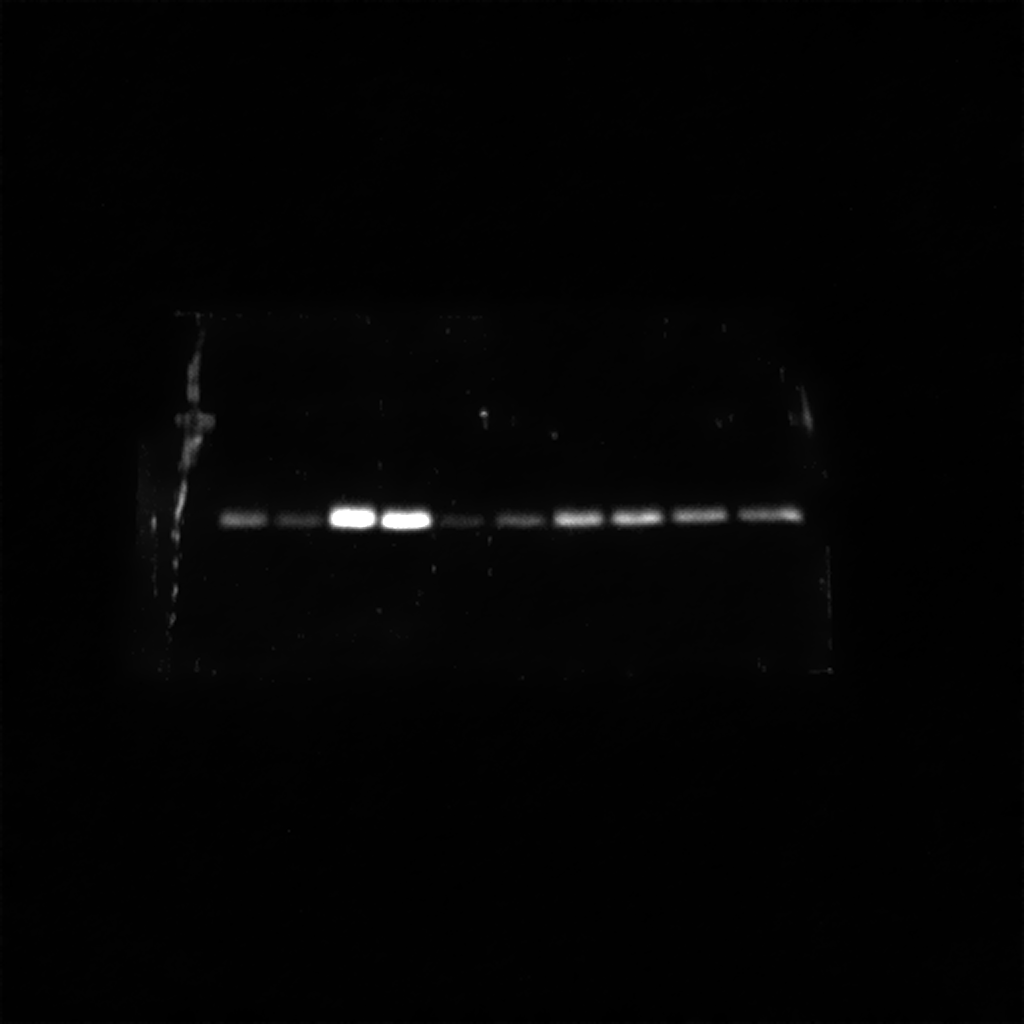

Supplement: Figure 3—source data 3. [file elife-75041-fig3-data3.zip › Fig 3 source data 3/Fig_3_supp1_source_data/petA.Tif]

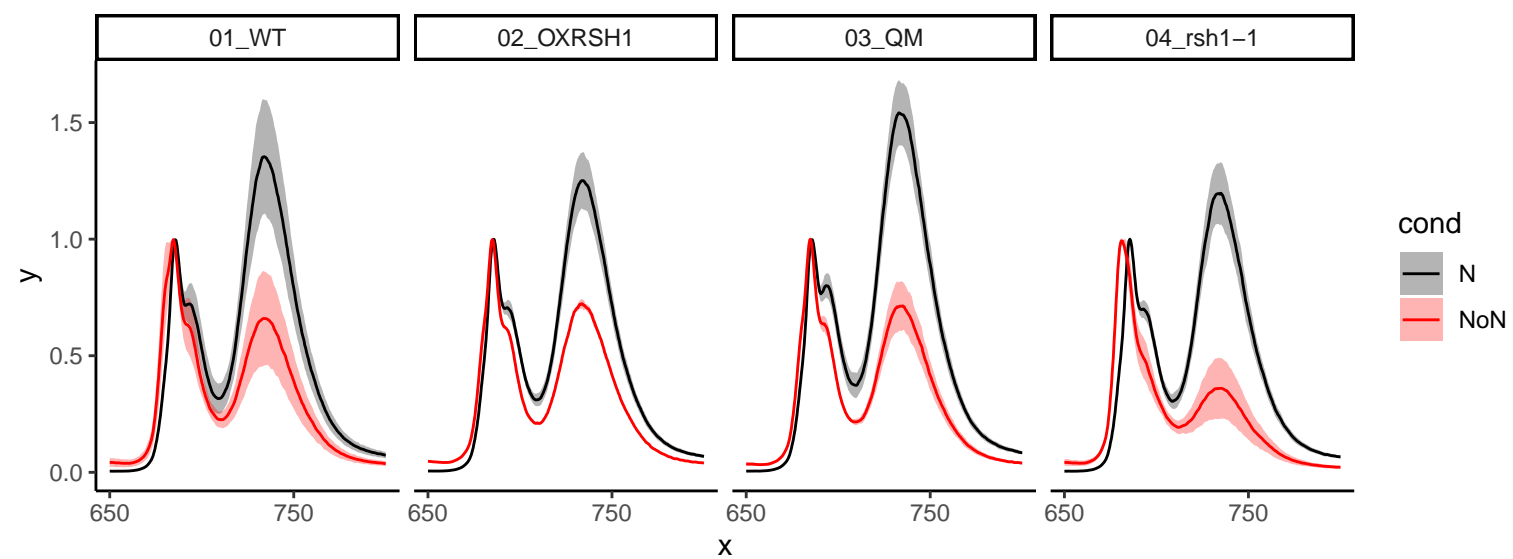

Supplement: Supplementary file 3. — Related to all figures. [file elife-75041-supp3.zip › R scripts/R markdown 77K plot Fig_3/full77K.pdf]

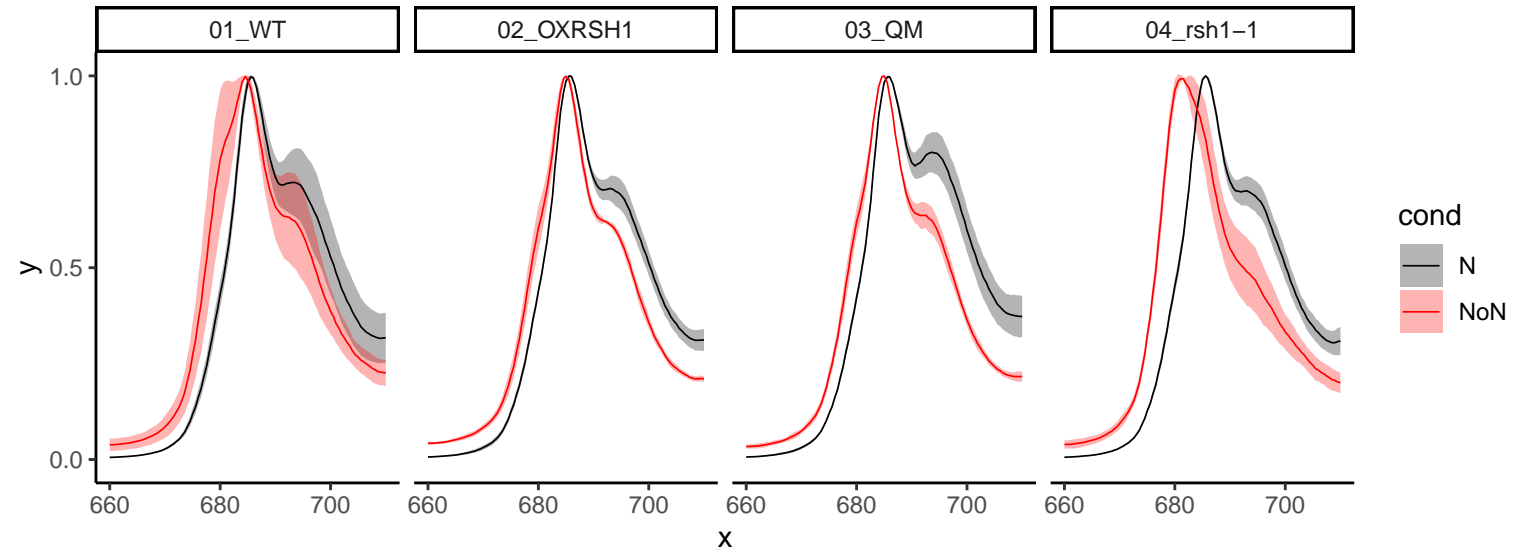

Supplement: Supplementary file 3. — Related to all figures. [file elife-75041-supp3.zip › R scripts/R markdown 77K plot Fig_3/zoom77K.pdf]

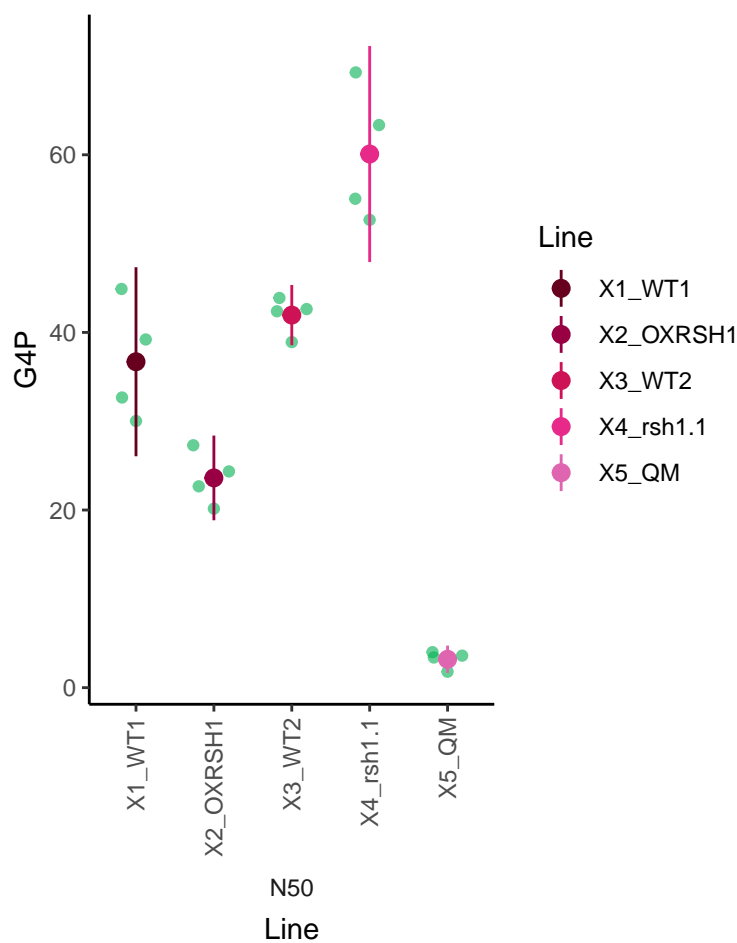

Supplement: Supplementary file 3. — Related to all figures. [file elife-75041-supp3.zip › R scripts/R markdown CI G4P Fig_1/my_ggplot1.pdf]

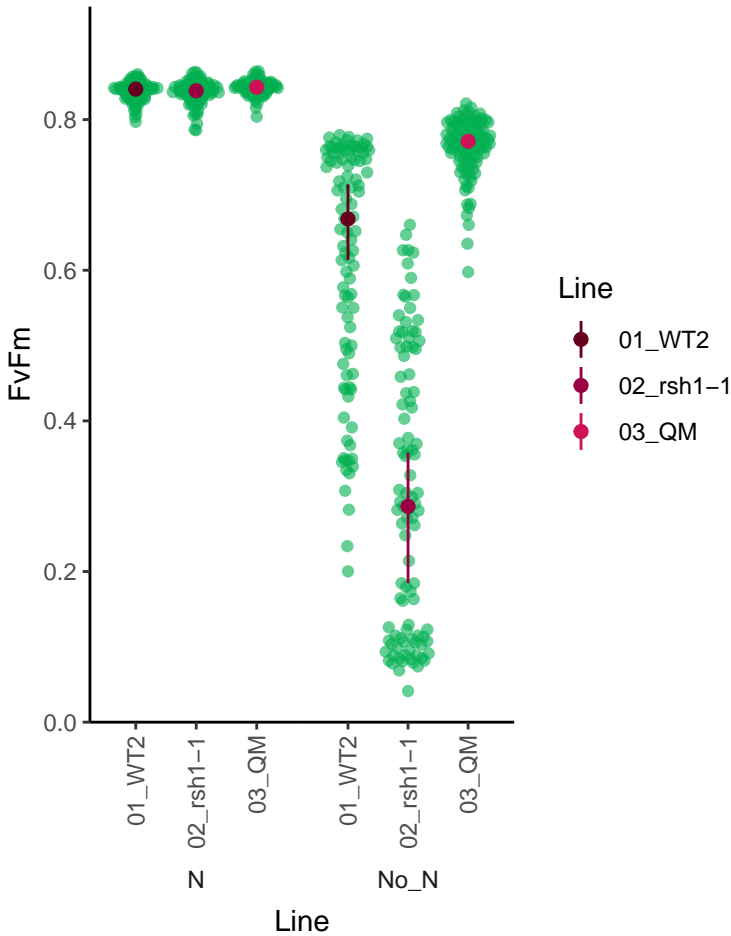

Supplement: Supplementary file 3. — Related to all figures. [file elife-75041-supp3.zip › R scripts/R markdown CI QY Fig_2/my_ggplot1.pdf]

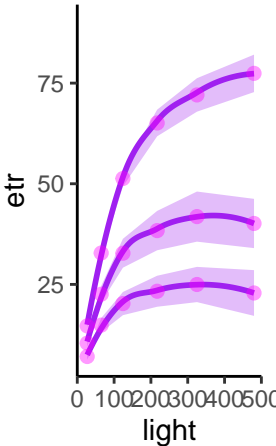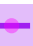

01\_qrt

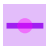

02\_QM

Supplement: Supplementary file 3. — Related to all figures. [file elife-75041-supp3.zip › R scripts/R markdown ETR Fig_2/ETRN50.pdf]

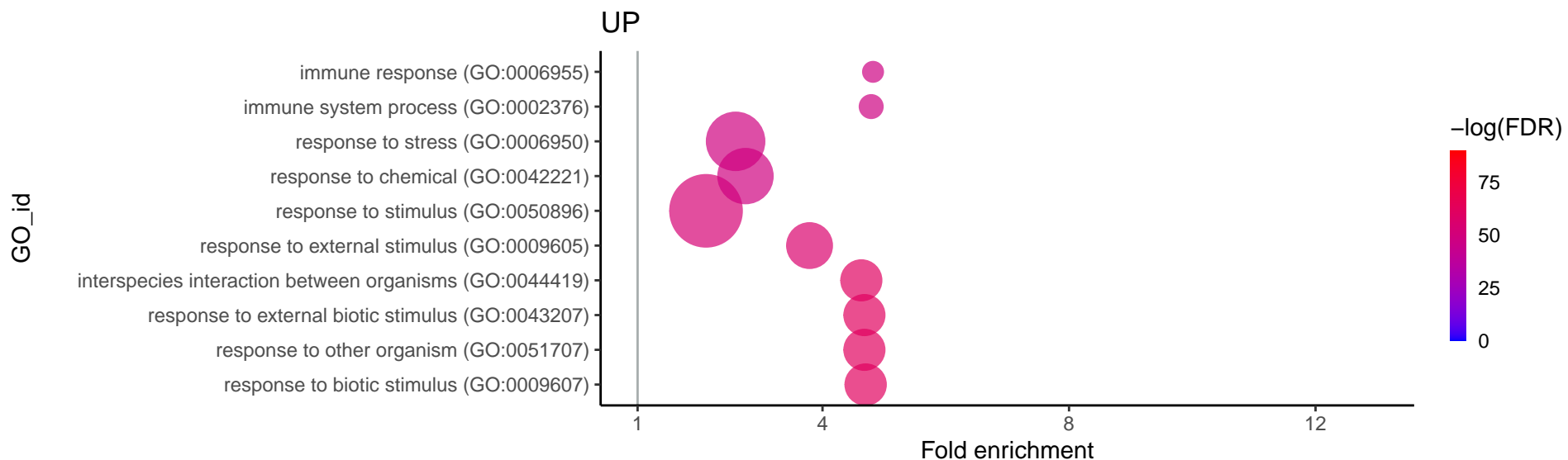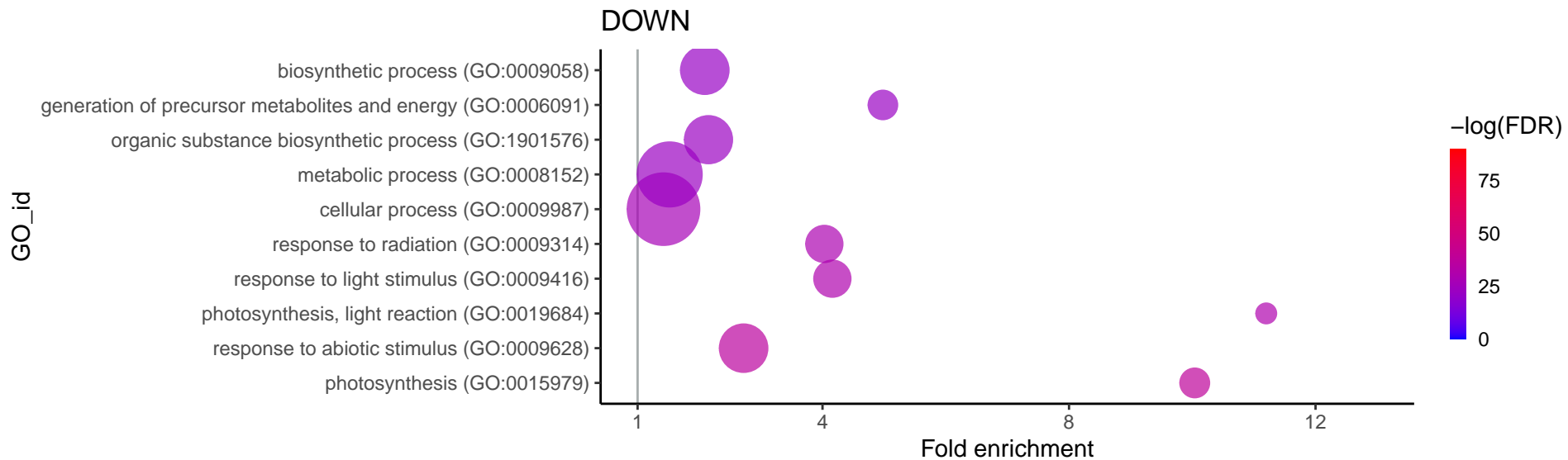

Supplement: Supplementary file 3. — Related to all figures. [file elife-75041-supp3.zip › R scripts/R markdown GO analysis Fig_4/GO.pdf]

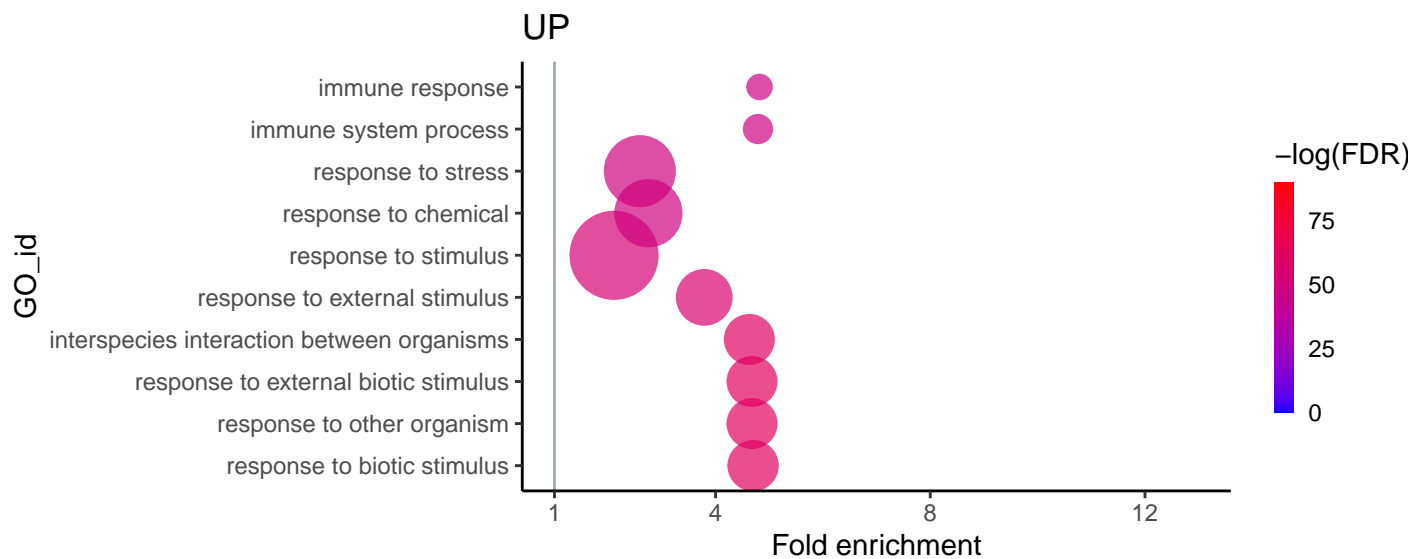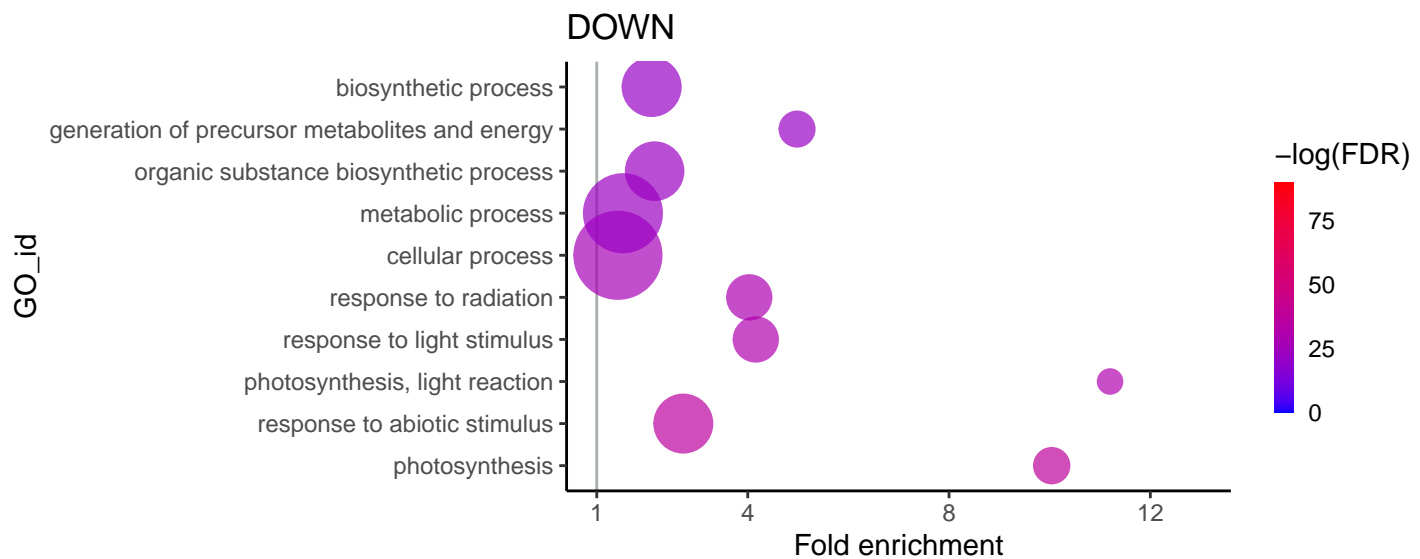

Supplement: Supplementary file 3. — Related to all figures. [file elife-75041-supp3.zip › R scripts/R markdown GO analysis Fig_4/hGO.pdf]
