# Supplementary material for: A guanosine tetraphosphate (ppGpp) mediated brake on photosynthesis is required for acclimation to nitrogen limitation in Arabidopsis
Source: eLife. 2022 Feb 14;11:e75041. doi: 10.7554/eLife.75041 (PMC8887892; doi:10.7554/eLife.75041)
Supplement: Figure 3—source data 2. [file elife-75041-fig3-data2.zip › Fig 3 source data 2/Fig3B_source_data_summary.pdf]

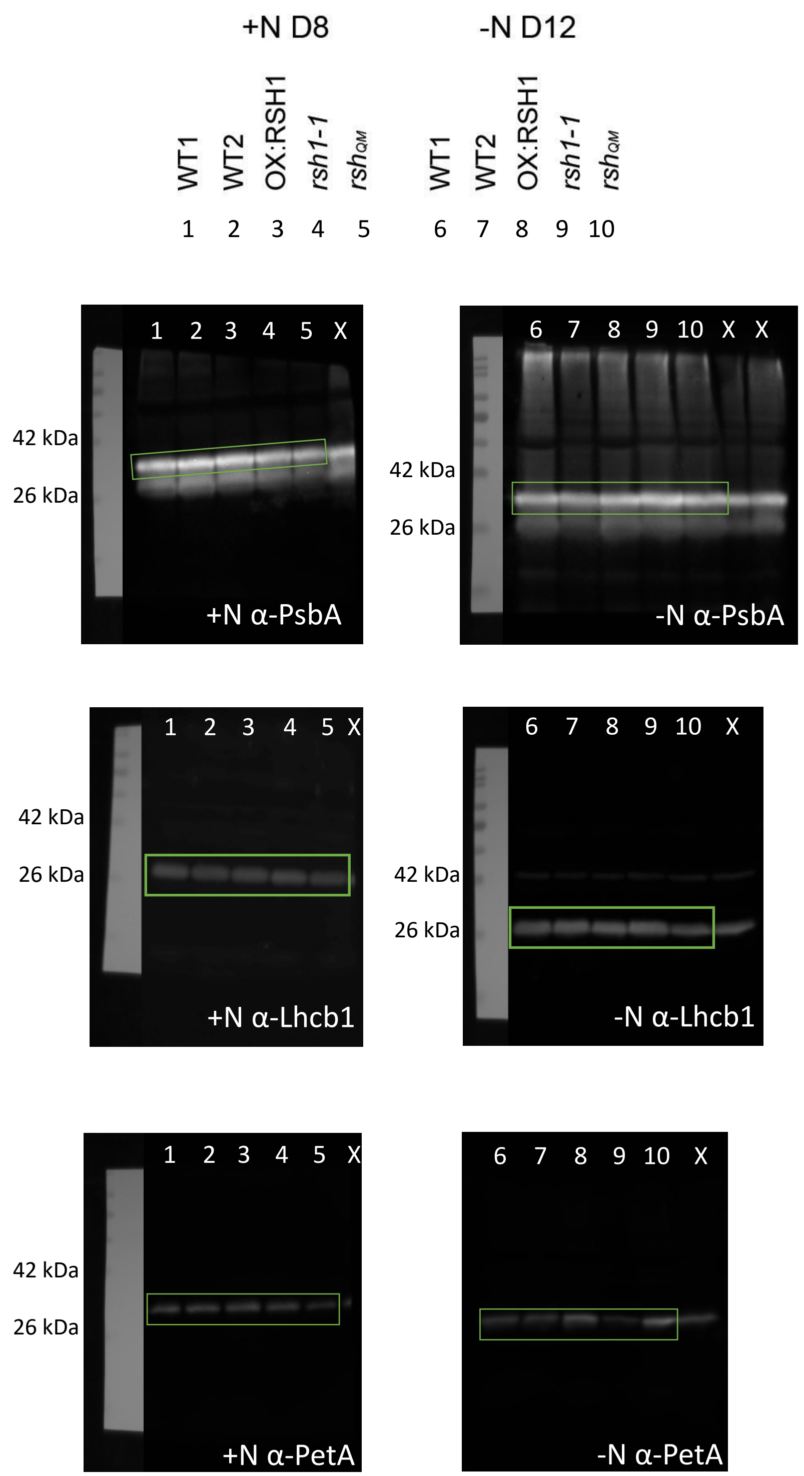

Figure 3B source data. Full sized immune blots.

NB : Immunoblot signal superimposed with photograph of marker lane taken at the same time. In some cases a photograph of the marker lane after transfer is also shown for clarity.

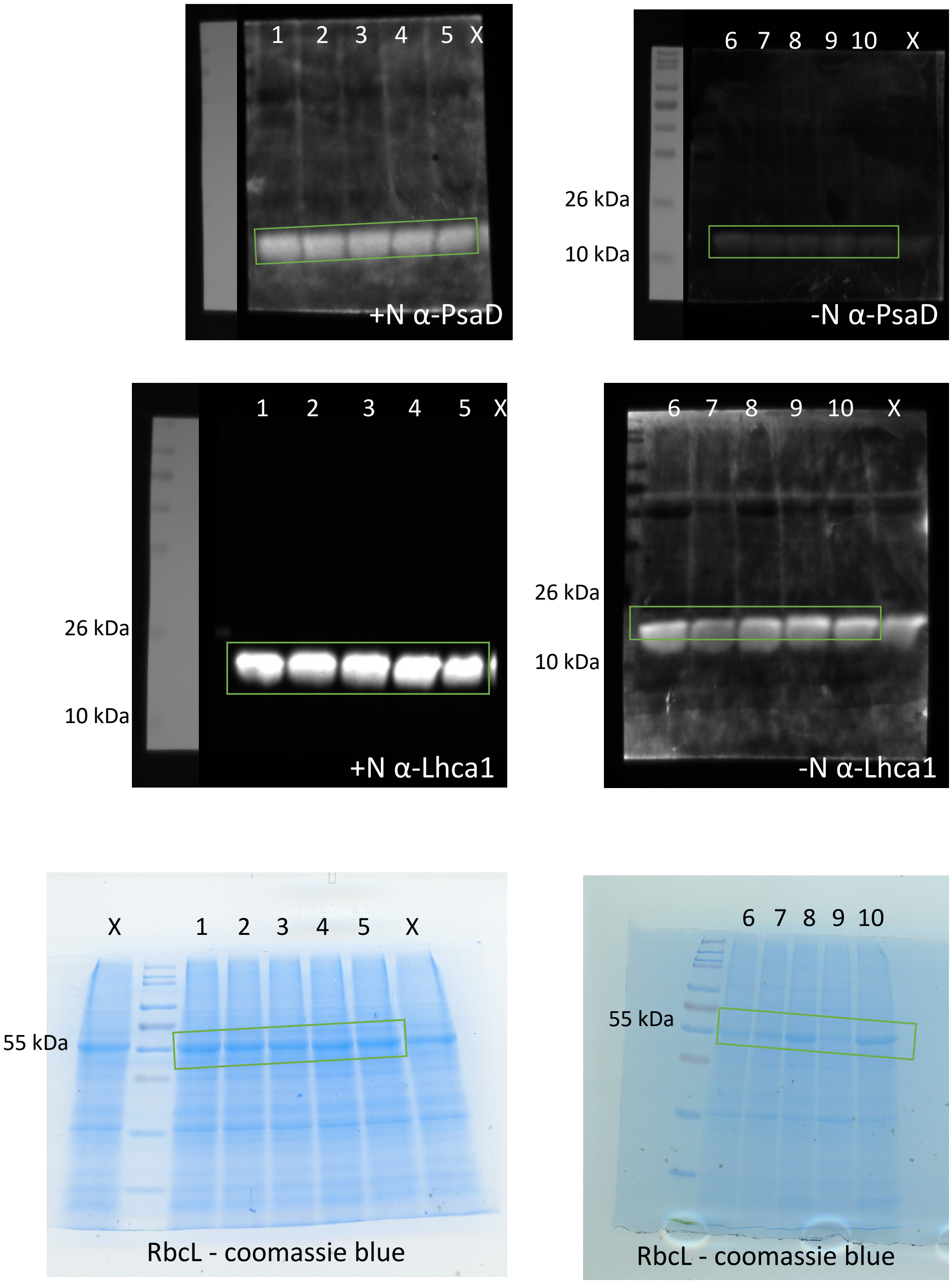

Figure 3B source data. Full sized immune blots.

NB : Immunoblot signal superimposed with photograph of marker lane taken at the same time. In some cases a photograph of the marker lane after transfer is also shown for clarity.
