# Supplementary material for: A guanosine tetraphosphate (ppGpp) mediated brake on photosynthesis is required for acclimation to nitrogen limitation in Arabidopsis
Source: eLife. 2022 Feb 14;11:e75041. doi: 10.7554/eLife.75041 (PMC8887892; doi:10.7554/eLife.75041)
Supplement: Figure 5—source data 3. [file elife-75041-fig5-data3.zip › Fig 5 source data 3/Fig 5 extended figure -N complexes WT vs OXRSH1.pdf]

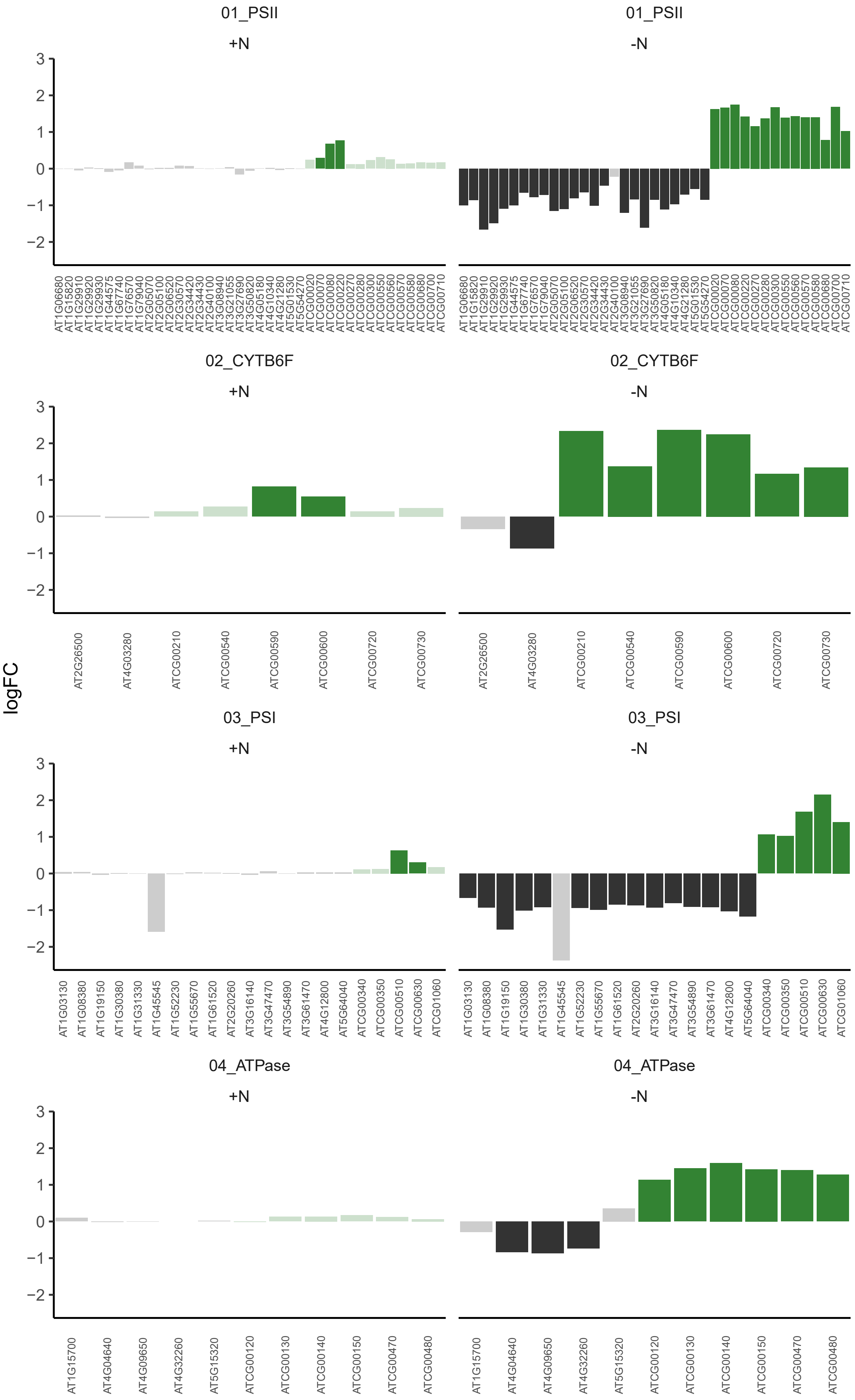
logFC
01_PSII
+N
-N
AT1G06680 AT1G15820 AT1G29910 AT1G29920 AT1G29930 AT1G44575 AT1G67740 AT1G76570 AT1G79040 AT2G05070 AT2G05100 AT2G06520 AT2G30570 AT2G34420 AT2G34430 AT2G40100 AT3G08940 AT3G21055 AT3G27690 AT3G50820 AT4G05180 AT4G10340 AT4G21280 AT5G01530 AT5G54270 ATCG00020 ATCG00070 ATCG00080 ATCG00220 ATCG00270 ATCG00280 ATCG00300 ATCG00550 ATCG00560 ATCG00570 ATCG00580 ATCG00680 ATCG00700 ATCG00710
02_CYTB6F
+N
-N
AT2G26500 AT4G03280 ATCG00210 ATCG00540 ATCG00590 ATCG00600 ATCG00720 ATCG00730
03_PSI
+N
-N
AT1G03130 AT1G08380 AT1G19150 AT1G30380 AT1G31330 AT1G45545 AT1G52230 AT1G55670 AT1G61520 AT2G20260 AT3G16140 AT3G47470 AT3G54890 AT3G61470 AT4G12800 AT5G64040 ATCG00340 ATCG00350 ATCG00510 ATCG00630 ATCG01060
04_ATPase
+N
-N
AT1G15700 AT4G04640 AT4G09650 AT4G32260 AT5G15320 ATCG00120 ATCG00130 ATCG00140 ATCG00150 ATCG00470 ATCG00480
3
2
1
0
−1
−2

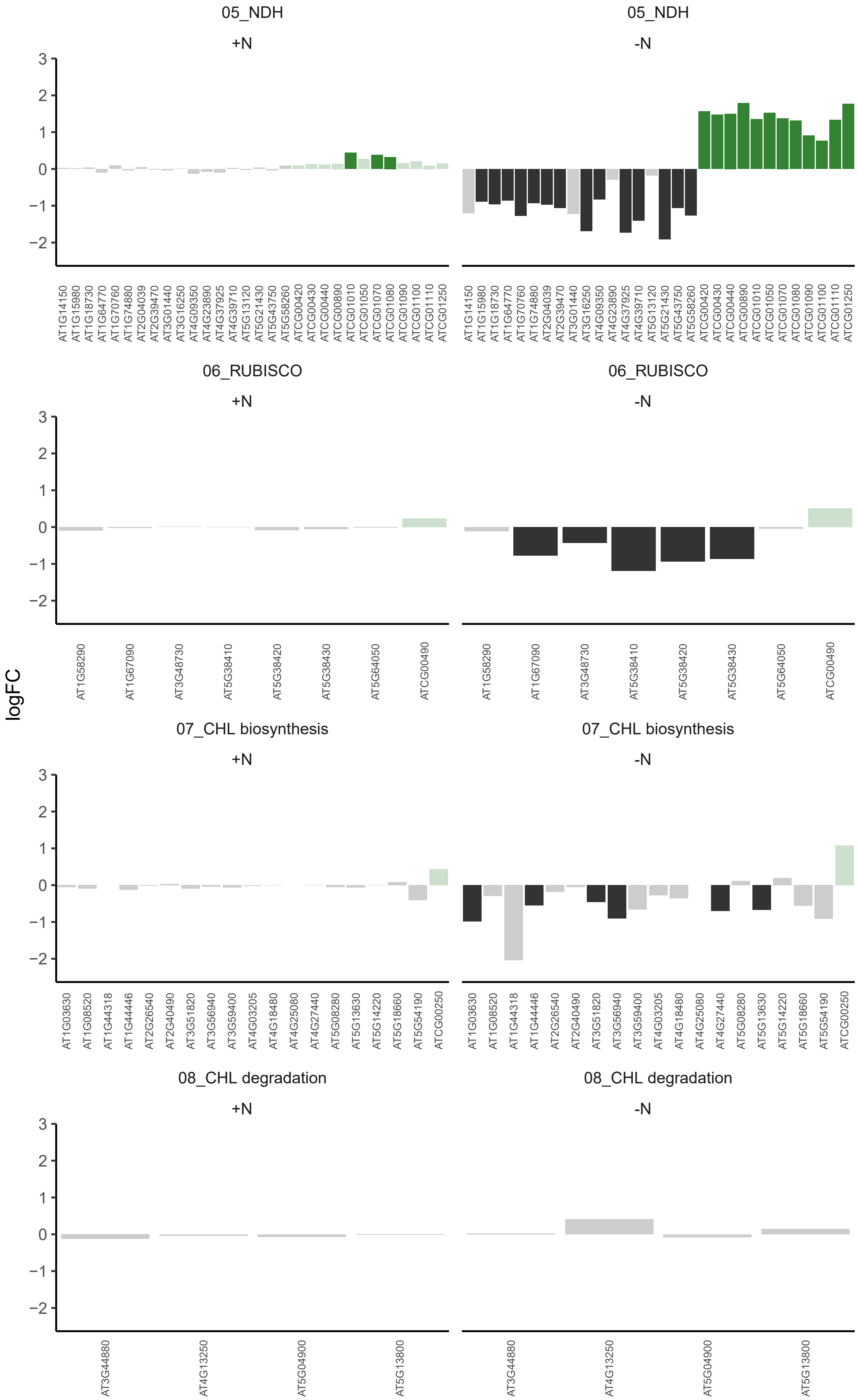
logFC
05_NDH
+N
05_NDH
-N
3
2
1
0
−1
−2
AT1G14150
AT1G15980
AT1G18730
AT1G64770
AT1G70760
AT1G74880
AT2G04039
AT2G39470
AT3G01440
AT3G16250
AT4G09350
AT4G23890
AT4G37925
AT4G39710
AT5G13120
AT5G21430
AT5G43750
AT5G58260
ATCG00420
ATCG00430
ATCG00440
ATCG00890
ATCG01010
ATCG01050
ATCG01070
ATCG01080
ATCG01090
ATCG01100
ATCG01110
ATCG01250
06_RUBISCO
+N
06_RUBISCO
-N
AT1G58290
AT1G67090
AT3G48730
AT5G38410
AT5G38420
AT5G38430
AT5G64050
ATCG00490
07_CHL biosynthesis
+N
07_CHL biosynthesis
-N
AT1G03630
AT1G08520
AT1G44318
AT1G44446
AT2G26540
AT2G40490
AT3G51820
AT3G56940
AT3G59400
AT4G03205
AT4G18480
AT4G25080
AT4G27440
AT5G08280
AT5G13630
AT5G14220
AT5G18660
AT5G54190
ATCG00250
08_CHL degradation
+N
08_CHL degradation
-N
AT3G44880
AT4G13250
AT5G04900
AT5G13800

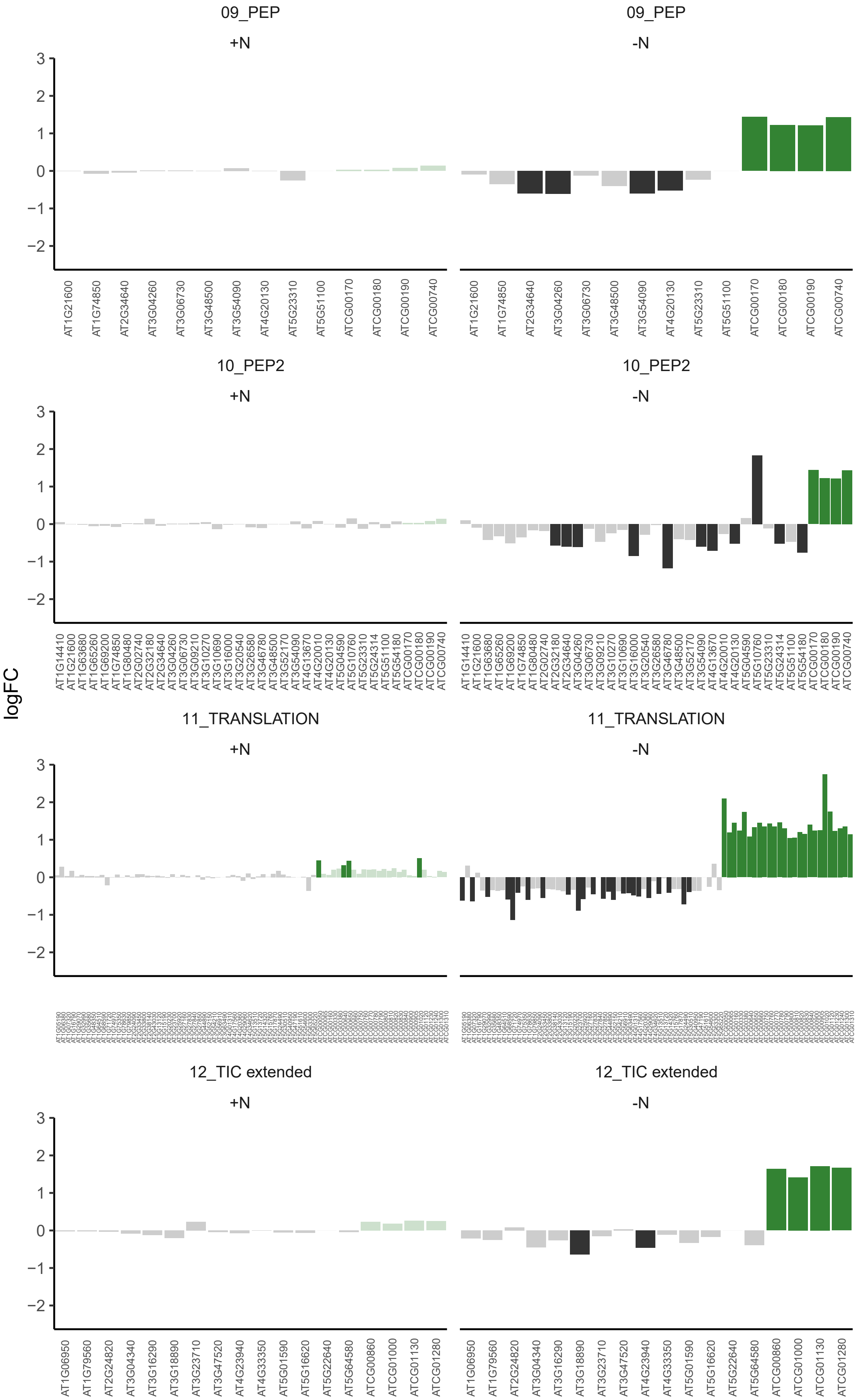

Figure 5- Extended figure. Disrupted regulation of photosynthetic complexes in OX:RSH1 versus wild type. Relative transcript levels under -N for OX:RSH1 versus wild type for nuclear (black) and chloroplast (green) genes encoding subunits of the indicated photosynthetic complexes. Solid colors indicate significantly different changes in expression, transparent colors indicate non-significant changes.
