# Supplementary material for: A guanosine tetraphosphate (ppGpp) mediated brake on photosynthesis is required for acclimation to nitrogen limitation in Arabidopsis
Source: eLife. 2022 Feb 14;11:e75041. doi: 10.7554/eLife.75041 (PMC8887892; doi:10.7554/eLife.75041)
Supplement: Figure 5—source data 3. [file elife-75041-fig5-data3.zip › Fig 5 source data 3/Fig 5 extended figure WT complexes +-N.pdf]

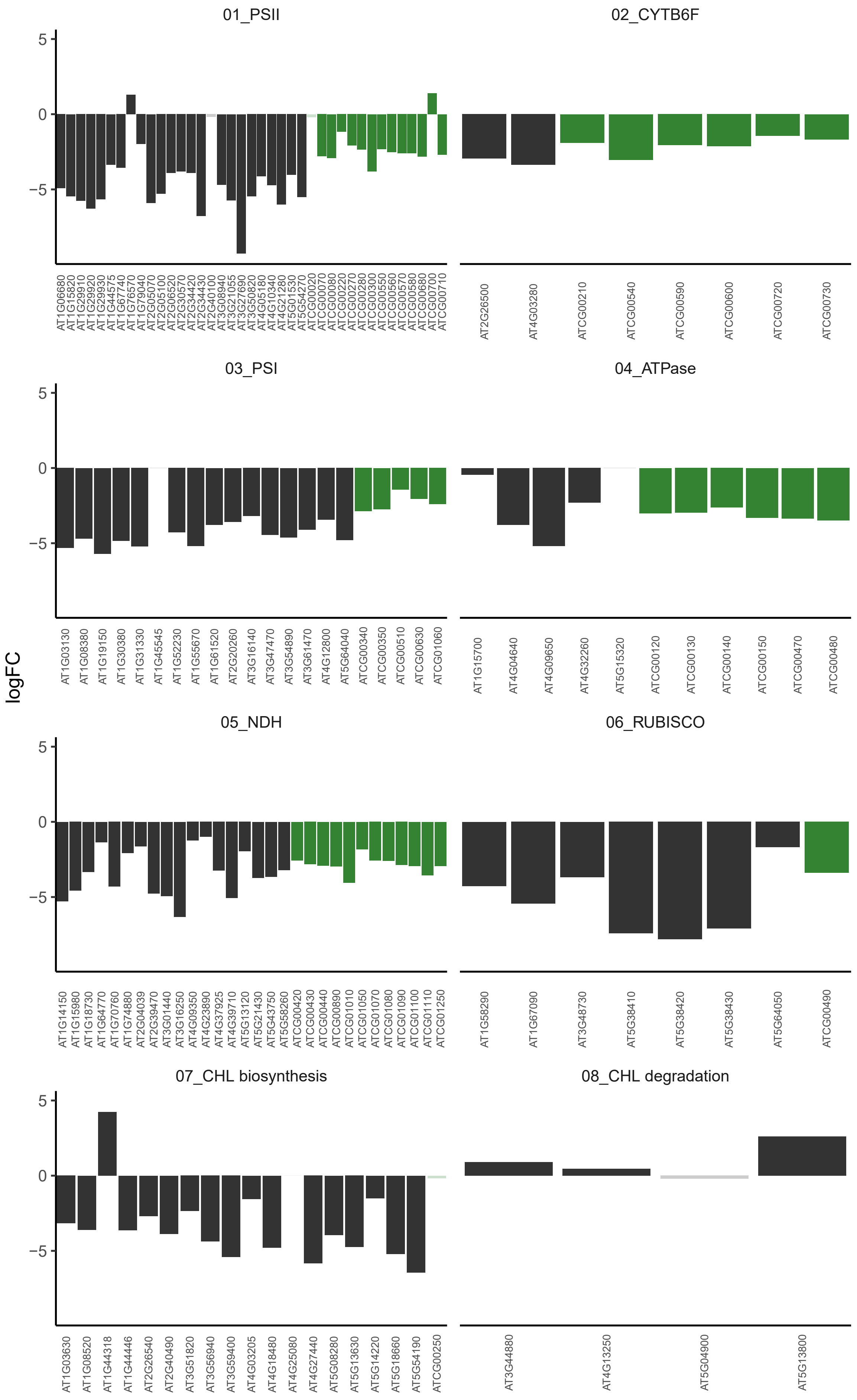
logFC
01_PSII
5
0
−5
AT1G06680
AT1G15820
AT1G29910
AT1G29920
AT1G29930
AT1G44575
AT1G67740
AT1G76570
AT1G79040
AT2G05070
AT2G05100
AT2G06520
AT2G30570
AT2G34420
AT2G34430
AT2G40100
AT3G08940
AT3G21055
AT3G27690
AT3G50820
AT4G05180
AT4G10340
AT4G21280
AT5G01530
AT5G54270
ATCG00020
ATCG00070
ATCG00080
ATCG00220
ATCG00270
ATCG00280
ATCG00300
ATCG00550
ATCG00560
ATCG00570
ATCG00580
ATCG00680
ATCG00700
ATCG00710
02_CYTB6F
AT2G26500
AT4G03280
ATCG00210
ATCG00540
ATCG00590
ATCG00600
ATCG00720
ATCG00730
03_PSI
5
0
−5
AT1G03130
AT1G08380
AT1G19150
AT1G30380
AT1G31330
AT1G45545
AT1G52230
AT1G55670
AT1G61520
AT2G20260
AT3G16140
AT3G47470
AT3G54890
AT3G61470
AT4G12800
AT5G64040
ATCG00340
ATCG00350
ATCG00510
ATCG00630
ATCG01060
04_ATPase
AT1G15700
AT4G04640
AT4G09650
AT4G32260
AT5G15320
ATCG00120
ATCG00130
ATCG00140
ATCG00150
ATCG00470
ATCG00480
05_NDH
5
0
−5
AT1G14150
AT1G15980
AT1G18730
AT1G64770
AT1G70760
AT1G74880
AT2G04039
AT2G39470
AT3G01440
AT3G16250
AT4G09350
AT4G23890
AT4G37925
AT4G39710
AT5G13120
AT5G21430
AT5G43750
AT5G58260
ATCG00420
ATCG00430
ATCG00440
ATCG00890
ATCG01010
ATCG01050
ATCG01070
ATCG01080
ATCG01090
ATCG01100
ATCG01110
ATCG01250
06_RUBISCO
AT1G58290
AT1G67090
AT3G48730
AT5G38410
AT5G38420
AT5G38430
AT5G64050
ATCG00490
07_CHL biosynthesis
5
0
−5
AT1G03630
AT1G08520
AT1G44318
AT1G44446
AT2G26540
AT2G40490
AT3G51820
AT3G56940
AT3G59400
AT4G03205
AT4G18480
AT4G25080
AT4G27440
AT5G08280
AT5G13630
AT5G14220
AT5G18660
AT5G54190
ATCG00250
08_CHL degradation
AT3G44880
AT4G13250
AT5G04900
AT5G13800

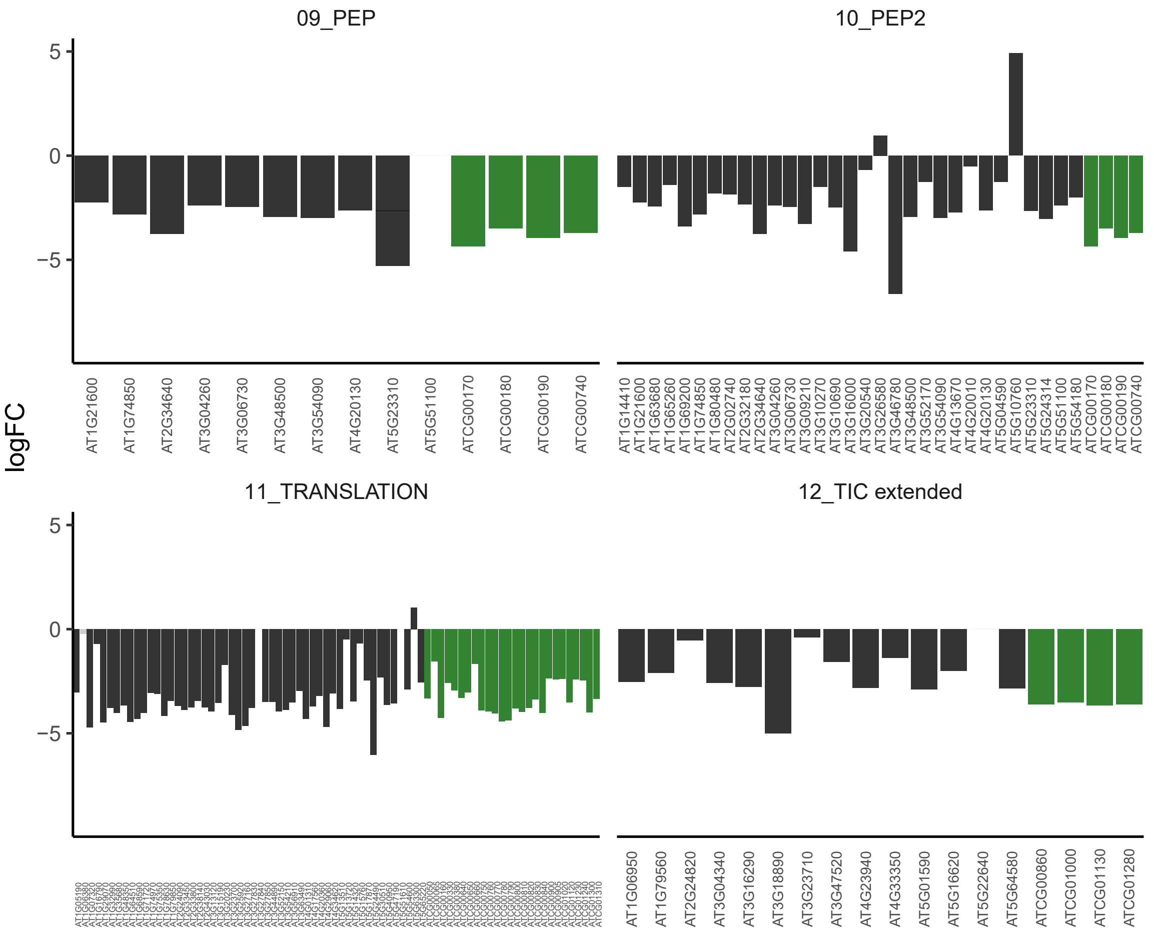

Figure 5- Extended figure. Coordinated regulation of photosynthetic complexes in the wild type, +N versus -N. Relative transcript levels in wild type -N versus +N for nuclear (black) and chloroplast (green) genes encoding subunits of the indicated photosynthetic complexes. Solid colors indicate significantly different changes in expression, transparent colors indicate non-significant changes.
